# Variations in the plasticity of functional traits indicate the differential impacts of abiotic and biotic factors on the structure and growth of trees in tropical dry forest fragments

**DOI:** 10.3389/fpls.2023.1181293

**Published:** 2024-01-25

**Authors:** Ravi Kant Chaturvedi, Santosh Kumar Pandey, Anshuman Tripathi, Laxmi Goparaju, Akhilesh Singh Raghubanshi, J. S. Singh

**Affiliations:** ^1^ Center for Integrative Conservation and Yunnan Key Laboratory for Conservation of Tropical Rainforests and Asian Elephant, Xishuangbanna Tropical Botanical Garden, Chinese Academy of Sciences, Menglun, Yunnan, China; ^2^ Ecosystems Analysis Laboratory, Department of Botany, Banaras Hindu University, Varanasi, Uttar Pradesh, India; ^3^ Training, Safety and Environment, National Mineral Development Corporation Limited, Dantewada, Chhattisgarh, India; ^4^ Forest and Remote Sensing, Vindhyan Ecology and Natural History Foundation, Mirzapur, Uttar Pradesh, India; ^5^ Institute of Environment and Sustainable Development, Banaras Hindu University, Varanasi, Uttar Pradesh, India

**Keywords:** community structure, plasticity in functional traits, resource-use strategy, fragmentation, tropical dry forest

## Abstract

Abiotic and biotic factors have considerable impact on the plasticity of plant functional traits, which influences forest structure and productivity; however, their inter-relationships have not been quantified for fragmented tropical dry forest (TDF) ecosystems. We asked the following questions: (1) what are the variations in the plasticity of functional traits due to soil moisture availability in TDF fragments? (2) what are the roles of soil nutrients and forest disturbances in influencing variations in the plasticity of functional traits in the TDF fragments? and (3) how do the variations in the plasticity of functional traits influence the structure and productivity of TDF fragments? Based on linear mixed-effects results, we observed significant variations among tree species for soil moisture content (SMC) under the canopy and selected functional traits across forest fragments. We categorized tree species across fragments by principal component analysis (PCA) and hierarchical clustering on principal components (HCPC) analyses into three functional types, *viz*., low wood density high deciduous (LWHD), high wood density medium deciduous (HWMD), and high wood density low deciduous (HWLD). Assemblage of functional traits suggested that the LWHD functional type exhibits a drought-avoiding strategy, whereas HWMD and HWLD adopt a drought-tolerant strategy. Our study showed that the variations in functional trait plasticity and the structural attributes of trees in the three functional types exhibit contrasting affinity with SMC, soil nutrients, and disturbances, although the LWHD functional type was comparatively more influenced by soil resources and disturbances compared to HWMD and HWLD along the declining SMC and edge distance gradients. Plasticity in functional traits for the LWHD functional type exhibited greater variations in traits associated with the conservation of water and resources, whereas for HWMD and HWLD, the traits exhibiting greater plasticity were linked with higher productivity and water transport. The cumulative influence of SMC, disturbances, and functional trait variations was also visible in the relative abundance of functional types in large and small sized fragments. Our analysis further revealed the critical differences in the responses of functional trait plasticity of the coexisting tree species in TDF, which suggests that important deciduous endemic species with drought-avoiding strategies might be prone to strategic exclusion under expected rises in anthropogenic disturbances, habitat fragmentation, and resource limitations.

## Introduction

Plant functional traits are a strong alternative compared to taxonomic identity for understanding plant performance and complex plant–plant and plant–environment interactions (Adler et al., 2014; Levine, 2016; Salguero-Gómez et al., 2016; Kunstler et al., 2016; Lourenço et al., 2022; Gómez et al., 2023). Moreover, along with genetic variations and developmental instability, changes in environmental conditions also induce variations in functional traits (i.e., phenotypic plasticity), and these variations enhance the ability to cope with shifting environments, where the species with greater variability adapt to a wide shift in environmental conditions compared to the species with lesser variability (Mitchell et al., 2016; Fox et al., 2019; Hofhansl et al., 2021; Kramp et al., 2022). The global climatic changes are influencing hydrological regime of terrestrial ecosystems, while the intensity and frequency of droughts are predicted to increase in the future (IPCC, 2013). Tropical trees across the globe are expected to experience increased drought stress (Rowland et al., 2021). Moreover, anthropogenic disturbances, land-use changes, and other activities, such as biomass exploitation by harvesting and grazing and soil fertility management, also have a large impact on the structure and function of tree-dominant ecosystems. Therefore, investigation of the variations in functional traits due to changing soil water and nutrient contents along disturbance gradients could help us understand the vulnerability of tropical trees to environmental changes (Anderegg et al., 2013; Zambrano et al., 2019; Gómez et al., 2023).

Important plant mechanisms for minimizing water stress in drought conditions are modification in leaf traits and decreasing transpirational water loss through a reduction in leaf lifespan (LL), leaf size or leaf area (LA), and specific leaf area (SLA) (Poorter et al., 2010; Liu et al., 2010; Pérez-Harguindeguy et al., 2013). Plants exposed to limited soil moisture content (SMC) generally exhibit lower leaf dry matter content (LDMC), while their leaf nitrogen content (LNC), leaf phosphorus content (LPC), and maximum saturated photosynthetic rate (A_max_) are higher (Pérez-Harguindeguy et al., 2013). Subsequently, the plants experiencing sufficient SMC show higher relative water content (RWC), chlorophyll content (Chl), and maximum saturated stomatal conductance (Gs_max_) (Chaturvedi et al., 2011a). Additionally, plants show greater intrinsic water use efficiency (WUEi) in water limited conditions (Chaturvedi et al., 2011a; Chaturvedi et al., 2011b). The leaf water potential at dawn (Ψ_dawn_) and leaf water potential at noon (Ψ_noon_) are important indicators of soil water availability, as is the status of water in leaves, where a more negative value indicates more dehydration of leaves (Pérez-Harguindeguy et al., 2013). Increasing anthropogenic disturbances, such as grazing, fires, habitat degradation, and fragmentation, have been reported to exhibit a decline in plant populations possessing leaf traits associated with resource conservation strategies, for instance, a higher LDMC (McIntyre, 2008; Carreño-Rocabado et al., 2012). However, these habitats show increases in plant populations with resource acquisition strategies linked with greater SLA, LNC, and A_max_ (Rodríguez-Alarcón et al., 2018; Bonilla-Valencia et al., 2020). Among wood traits, saturated stem water content (QWsat) is greater in water limited conditions, whereas wood specific gravity (WSG) is generally higher in habitats with sufficient SMC (Chaturvedi et al., 2021). Studies have shown that WSG partly underlies the growth–survival trade-off, where a low WSG indicates fast growth while a high WSG represents greater survival (Pérez-Harguindeguy et al., 2013). Among whole plant traits, tree foliage cover intensity (CC), tree height to DBH ratio (HTDBH), crown depth to DBH ratio (CDDBH), and crown cover to DBH ratio (CCDBH) are greater in habitats with higher SMC and soil nutrients (Chaturvedi, 2010; Chaturvedi et al., 2021). For the reproductive traits, seed mass (SDWT) is generally greater at low disturbed sites with higher SMC compared to high disturbed sites with low SMC (Khurana and Singh, 2006). For biomass accumulation in trees under drought conditions, there is conflicting evidence, where increases, decreases, or even no change have been observed for tropical trees (e.g., Adams et al., 2017). However, extreme or extended droughts potentially reduce biomass accumulation and lead to the mortality of trees (Mitchell et al., 2013).

Plants exhibit divergent strategies to drought and disturbances which lead to uncertainty in forecasting the structure and productivity of future forests across the globe (McDowell et al., 2018; Anderegg et al., 2020; Silva et al., 2020). Among the drought strategies, plants can be evaluated based on their capacity to avoid or tolerate drought (Levitt, 1972). While the plants under drought-avoiding functional type suspend their physiological functions and remain dormant during drought periods, the drought-tolerant functional type have the capacity to maintain physiological functions at the minimum cell water content (Poorter and Markesteijn, 2008). In tropical dry forests, the drought-avoiding functional type characteristically exhibits leaf deciduousness and low wood density as mechanisms to reduce water loss, whereas the drought-tolerant functional type has a longer leaf lifespan and higher wood density as mechanisms to resist xylem cavitation (Chaturvedi et al., 2021). According to Grime’s model (Grime, 1988), the vascular plants can be classified into three main evolutionary strategies based on the intensity of disturbance and stress. Among these strategies, the competitive strategy (C) is adopted by plants growing in habitats with low stress and low disturbance, and their survival depends on the ability to compete for the available resources. The stress tolerance strategy (S) is adopted by plants growing in habitats with high stress and low disturbance, where the survival of plants depend on their endurance to low resource conditions. The ruderal strategy (R) is adopted by the plants growing in low stress and high disturbance environments, and these plants either exhibit short lifespans and high seed production (annual herbs) or act as pioneers (trees). Based on the Grime’s model, the drought-avoiding functional type may adopt the R strategy and act as pioneers, whereas the drought-tolerant functional type may exhibit a continuum of strategies from C to S according to their capacity to compete for resources and their ability to tolerate drought stress.

Recently, for understanding the resistance of plant species to drought stress and for the maintenance of biodiversity (Violle et al., 2012; Kramp et al., 2022), research interest in inter- and intra-specific drought-resistance trait variations has increased (Choat et al., 2007; Cornwell and Ackerly, 2009; Luo et al., 2023). These studies have mainly evaluated the influence of abiotic (e.g., Valladares et al., 2006; Bongers et al., 2018) and biotic (e.g., Callaway et al., 2003; Díaz et al., 2007; Metlen et al., 2009; Li et al., 2021) factors on plasticity in functional traits separately, whereas a few studies have also reported mixed results of the effects of biotic and abiotic factors for the plastic responses to plant competition (e.g., Semchenko et al., 2007; Cahill et al., 2010). Furthermore, some studies have also reported the interactive impact of abiotic and biotic factors producing a complex plastic response (e.g., Wang et al., 2017; Wang and Callaway, 2021). However, these studies are mostly documenting findings from *ex situ* manipulative experiments, and *in situ* observations on the impact of biotic and abiotic factors on the plasticity of functional traits in forest fragments have rarely been evaluated (see Choat et al., 2007; Li et al., 2022).

Our study aims to observe the variations in plasticity of functional traits of trees in fragmented tropical dry forests across water availability gradients at small spatial scales. Our investigation will provide clues for the identification of species responses to key environmental factors in tropical dry forests, i.e., soil water and nutrient content, and their interactions with other important and wide spread global change threats such as fragmentation. We asked the following questions: (1) what are the variations in the plasticity of functional traits due to soil moisture availability in tropical dry forest fragments? (2) what are the roles of soil nutrients and forest disturbances in influencing variations in the plasticity of functional traits in tropical dry forest fragments? and (3) how does the variation in the plasticity of functional traits influence the structure and productivity of tropical dry forest fragments? We hypothesize that the phenotypic plasticity in response to water and nutrient contents in soils and anthropogenic disturbances play an important role in shaping the niche of tropical dry forest trees. Thus, declining soil resources and increasing disturbances will support the assemblage of less productive trees with increasing plasticity in functional traits, representing defense against drought stress, nutrient deficiency, and disturbances.

## Materials and methods

### Study sites

**Figure 1:**
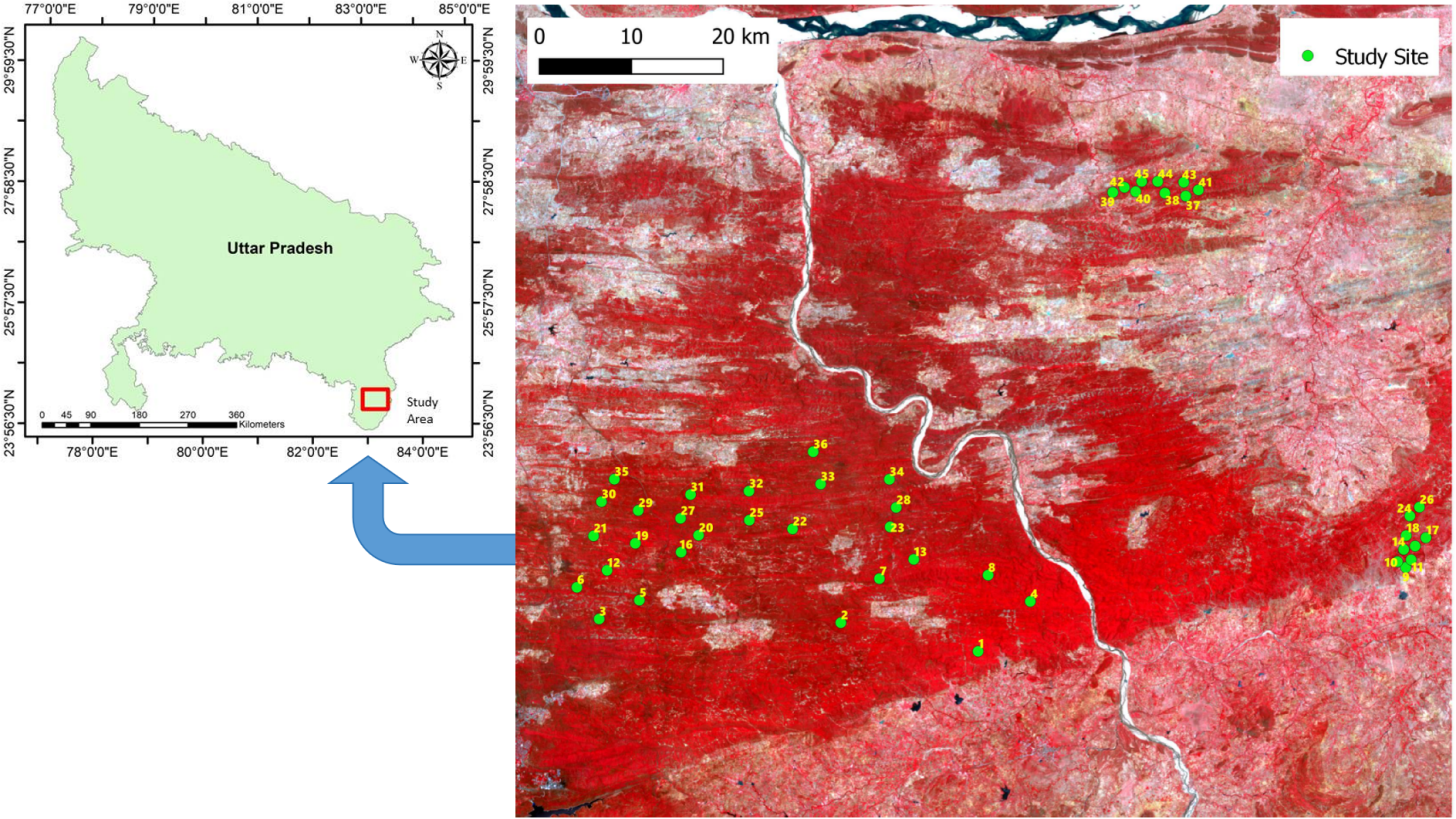
Map showing the locations of the 45 study sites in Uttar Pradesh state, India.

Our investigation was conducted in 45 forest fragments or patches or study sites in the Vindhyan highlands, situated in the Sonebhadra district of Uttar Pradesh, India (**Figure 1**; **Supplementary Table S1**). These fragments cover around a 50 km radius, and the distance between the two nearest fragments is around 2 km. The size of largest fragment is 92.4 ha, while that of the smallest fragment is 1.5 ha. The patch perimeter and edge distance of these fragments range from 0.4 to 4.0 km, and 0.07 to 0.4 km, respectively. The altitude for these sites ranges from 231 to 350 m above sea level. The selected sites contain naturally established old-growth forests. The forest region was exposed to anthropogenic disturbances by local villagers in the form of the extraction of forest resources, illegal harvesting, grazing by cattle, and occasional burning, although in the past two decades government policies have been engaged in controlling disturbances, mainly in large size fragments. The idea behind the site selection was to get greater variations in soil moisture content (SMC), to cover the maximum possible tree species diversity of the region, and to get a gradient of disturbance. The area experiences a tropical monsoon climate. We collected monthly climatic and weather data for a 12-year period starting from January 2008 to December 2019 from the website www.worldweatheronline.com. The data include minimum, average, and maximum temperatures, rainfall, and relative humidity. The average temperature during this period ranged from 10.5 °C in January to 42.5 °C in May. The average annual rainfall was 662.3 mm, with a minimum rainfall of 2.69 mm in November to a maximum of 208.7 mm in July. The study region experienced more than 80% of the total annual rainfall during only three months (July–September), and the remaining 20% in the other nine months. Similar to rainfall, the relative humidity in the study region was also higher (>70%) during July–September period, with the minimum and maximum relative humidity during this period being 22.0% in April and 76.4% in August. More detailed descriptions of the study region is given in Chaturvedi (2010).

### Sampling design

We established three rectangular plots (100 m × 50 m) randomly in the central region of each forest fragment for periodical measurements. The distance from the forest edge for each plot was recorded in four directions, and the average value was considered as the edge distance. For fragments whose edge distance was ≥200m, the three plots inside the fragment were separate from each other by a distance of at least 50m, while for the fragments whose edge distance was<200m, we tried to keep a distance of at least 25m between the plots. We counted the stems and measured their diameter at breast height (DBH) for all tree species inside each plot using a measuring tape and identified all individuals with a ≥10 cm DBH for further measurements. In each plot, growth measurements were recorded for two years, starting from September 2010 to August 2012. Increases in girth for all individuals were measured annually with the help of metal dendrometer bands fitted at a height of 1.37 m for each species (Chaturvedi et al., 2011c; Chaturvedi et al., 2017). We also measured height increments for each selected individual inside each plot with the help of a 15 m graduated measuring pole for individuals up to 15 m height (one person holding the pole and a second acting as a ‘‘spotter’’ to assess the mark on the pole which reached the level of the top of the crown), and for taller individuals, the trigonometric method was applied. For the measurement of functional traits, including whole plant traits, wood traits, leaf traits, and reproductive traits, we marked at least five individuals with a ≥ 10 cm DBH for each tree species inside the three rectangular plots in each fragment. For a few species whose individuals were less than five inside the three plots, we marked the remaining individuals outside the plot boundaries. Sampling for most of these measurements was done in September 2010; however, a few rare species which were not sampled in the year 2010 were covered in 2011.

Composite surface (0–30 cm) soil samples were collected at five random locations from each plot but only once in September 2010 for the physico-chemical analysis. The soil samples collected from the three plots in each forest fragment were pooled together for further analysis. These soil samples were analyzed for texture (Sheldrick and Wang, 1993), organic carbon (Walkley and Black, 1934), total nitrogen (Bremner and Mulvaney, 1982), and total phosphorus (Olsen and Sommers, 1982) contents. To get the actual organic carbon content, values obtained using the Walkley and Black method were multiplied by a correction factor (1.95) given by Krishan et al. (2009) for similar soils of central India. The correction factor is based on the relationship between the Walkley and Black estimate and that from the oxidative combustion-infrared analysis method using a total organic carbon analyzer. Soil bulk density at each site was determined using the core method (Krzic et al., 2000). Soil moisture content (SMC) at a depth of 10 cm was measured every month for two years, starting from September 2010 to August 2012, using a theta probe instrument (type ML 1, Delta-T Devices, Cambridge, UK), as percentage by volume under the canopy, on four sides of the main trunk, at a distance of one meter from each marked individual tree species.

### Tree mortality and recruitment data

We recorded tree mortality and recruitment in each plot annually and averaged the data for the three plots for each site. Under mortality, we recorded the number of trees that died due to drought, fire, and harvesting by humans each year, while recruitment was considered as the number of trees attaining 10 cm DBH within the year. The annual mortality index (MI, %) was calculated as:


$$MI\;=(\frac{T_{D}}{T_{F}+T_{N}})\times \;100$$

where T_D_ is the number of newly died trees within a year, T_F_ is the number of trees during first measurement of the year, and T_N_ is the number of newly recruited trees in a year.

### Functional trait data

We selected 20 functional traits considered important for tropical dry forest trees (Chaturvedi et al., 2012) (**Table 1**), including four whole plant traits, *viz.*, tree foliage cover intensity/canopy cover intensity (CC), tree height to DBH ratio (HTDBH), crown depth to DBH ratio (CDDBH), and crown cover to DBH ratio (CCDBH), two wood traits, *viz.*, wood specific gravity (WSG) and saturated stem water content (QWsat), six morphological leaf traits, *viz.*, leaf size or leaf area (LA), specific leaf area (SLA), relative water content (RWC), leaf dry matter content (LDMC), leaf water potential at dawn (Ψ_dawn_), and leaf water potential at noon (Ψ_noon_), six physiological leaf traits, *viz.*, leaf nitrogen content (LNC), leaf phosphorus content (LPC), maximum saturated stomatal conductance (Gs_max_), maximum saturated photosynthetic rate (A_max_), intrinsic water use efficiency (WUEi), and chlorophyll content (Chl), one phenological leaf trait, *viz.*, leaf lifespan (LL), and one reproductive trait, *viz.*, seed mass (SDWT).

**Table 1:** List of functional traits investigated in this study with their abbreviations, units, and functional dimension.

	Functional traits	Abbreviation	Unit	Functional dimension
Whole plant traits	Canopy cover intensity	CC	%	Light interception, Plant economics
	Tree height	HT	m	Light interception
	Crown depth	CD	m	Light interception
	Crown cover	CC	m^2^	Light interception
Wood traits	Wood specific gravity	WSG	g cm^-3^	Water acquisition, support, and strength
	Saturated stem water potential	QWsat	%	Water acquisition
Morphological leaf traits	Leaf area	LA	cm^2^	Light interception
	Specific leaf area	SLA	cm^2^g^-1^	Plant economics
	Relative water content	RWC	%	Water acquisition
	Leaf dry matter content	LDMC	%	Plant economics
	Leaf water potential at dawn	Ψ_dawn_	MPa	Water acquisition
	Leaf water potential at noon	Ψ_noon_	MPa	Water acquisition
Physiological leaf traits	Leaf nitrogen content	LNC	%	Plant economics
	Leaf phosphorus content	LPC	%	Plant economics
	Saturated stomatal conductance	Gs_max_	mol m^-2^s^-1^	Plant productivity
	Saturated photosynthesis rate	A_max_	μmol m^-2^s^-1^	Plant productivity
	Intrinsic water use efficiency	WUEi	μmol mol^-1^	Plant economics
	Chlorophyll content	Chl	mg g^-1^	Light interception
Phenological leaf trait	Leaf life-span	LL	days	Plant economics
Reproductive trait	Seed mass	SDWT	g	Reproductive capacity

The tree CC was recorded for all marked individuals of each species monthly for two years, starting from September 2010 to August 2012. For this observation, we tagged 20 to 50 terminal twigs on the four sides of each marked individual. We recorded the date of leaf budding or leaf flushing and leaf shedding (including the time of yellowing, browning, and partial or total leaf shedding) for each marked individual at each plot in each forest fragment. During these observations we also recorded the leaf lifespan (LL) and proportion of leaves in the canopy under a particular phenophase or phenological event. For estimation of the intensity of phenological events (*viz.*, CC), we followed Fournier (1974) and quantified the phenophases for each individual from zero to four (Fournier intensity index). Among these five indices, zero represents absence of a phenophase, one indicates the intensity of a phenophase between 1-25%, two between 26-50%, three between 51-75%, and four between 76-100%. We calculated the intensity of phenophases for foliage cover for each species in each forest fragment using the formula:


$$Phenophase\;intensity=\left(\frac{\sum^{\;}Fournier\;intensity}{4N}\right)\times 100$$

where, ∑Fournier intensity is the sum of the Fournier intensity for all individuals of a species and N is the number of individuals of the species.

Crown depth for each individual tree was measured on four sides of the tree as the length along the main axis from the top of the tree to the base of the crown. Similarly, crown cover for each tree was measured as the area covered by the vertical projection of the perimeter of the crown. WSG was measured for the five individuals of each tree species inside the three plots at each of the 45 forest fragments following the protocol given in Chaturvedi et al. (2010). The individuals selected for the measurement of WSG were different from those which were marked for the measurement of tree growth, and samples were collected from the trees outside the plots. We took wood samples from the main trunk at the height of 1.4 m, up to the radial depth, with the help of stem borer. The wood samples were sealed in plastic bags separately and brought to the laboratory. The volumes of fresh wood samples, after removing bark, were estimated using the water displacement method, and the wood samples were dried in oven at 80 °C until constant weight. The values of WSG are expressed as g cm^-3^. For determining the amount of saturated stem water (QWsat, %), we collected stem samples > 3 cm diameter from the same five individuals which were selected for WSG measurement for each species. The volume and dry weight of stem samples were estimated after removing the outer bark by following the same method that was applied during the measurement of WSG; however, before measuring the volume, the stem samples were soaked in water overnight. The formula used for obtaining QWsat, according to Borchert (1994), was:


$$QWsat=(\frac{Stem\;weight_{\left(water\;saturated\right)}\;-\;Stem\;weight_{\left(dry\right)}}{Stem\;weight_{\left(dry\right)}})\times 100$$

Leaf traits were measured on the same trees which were marked for the study of phenology. While we measured LA, SLA, LDMC, LNC, LPC, A_max_, Gs_max_, WUEi, Ψ_dawn_, and Ψ_noon_ according to Pérez-Harguindeguy et al. (2013), RWC was measured according to Tanentzap et al. (2015). For the measurement of LA, SLA, LDMC, RWC, and Chl, 10 to 20 fully expanded, mature, and sun-facing leaves were collected from each marked individuals of each tree species. Fresh leaf weights for all leaves were recorded just after collection, at the field site, using a portable electronic weighing balance. After weighing, the leaves were wrapped separately in moist paper for rehydration, sealed in separate plastic bags, and brought to the laboratory. All measurements were done within 24 hours of bringing the samples to the laboratory. Chlorophyll was analyzed by crushing 0.1 g of the leaf in 10 ml 80% acetone (Aron, 1949). The absorbance (D) of the extract was then measured at 645 and 663 nm using 80% acetone as a blank control. The concentrations of Chl_a_ and Chl_b_ were calculated from the following expressions:


$$Chl_{a}\;(mg\;g^{\mathrm{-1}})=\left(\left[12.7\times D_{663}\right]-\left[2.60\times D_{645}\right]\right)\times \frac{volume\;of\;acetone\;(10ml)}{weight\;of\;leaf\;tissue\;(0.1g)}$$

$$Chl_{b}\;(mg\;g^{\mathrm{-1}})=\left(\left[22.9\times D_{645}\right]-\left[4.68\times D_{663}\right]\right)\times \frac{volume\;of\;acetone\;(10ml)}{weight\;of\;leaf\;tissue\;(0.1g)}$$

$$Chl\;(mg\;g^{\mathrm{-1}})=Chl_{a}(mg\;g^{\mathrm{-1}})+Chl_{b}\;(mg\;g^{\mathrm{-1}})$$

After Chl measurement, the remaining fresh leaves were rehydrated, weighed on an electronic balance, scanned on a table scanner, and their dimensions were determined with the help of the Image-J program (Abramoff et al., 2004) for the measurement of LA. After LA measurements, all leaf samples were dried in separate paper bags in an oven at 70°C until constant weight. After recording the leaf fresh weight, leaf rehydrated fresh weight, and leaf dry weight, we calculated SLA, LDMC, and RWC by using the following equations:


$$SLA=(\frac{Leaf\;area\;(cm^{2})}{Leaf\;dry\;weight\;(g)})$$

$$LDMC=(\frac{Leaf\;dry\;weight\;(g)}{Leaf\;rehydrated\;fresh\;weight\;(g)})\times 100$$

$$RWC=(\frac{Leaf\;fresh\;weight\;(g)\;-\;Leaf\;dry\;weight\;(g)}{Leaf\;rehydrated\;fresh\;weight\;(g)\;-\;Leaf\;dry\;weight\;(g)})\times 100$$

LNC and LPC were measured using micro-Kjeldahl (acidic) digestion followed by colorimetric (flow-injection) analysis.

For the measurement of A_max_ (µmol m^-2^ s^-1^), Gs_max_ (mol m^-2^ s^-1^), Ψ_dawn_ (MPa), and Ψ_noon_ (MPa), we sampled twigs from each marked tree species at mid-canopy height having full sun exposure for at least part of the day and with healthy and fully expanded leaves. Measurements for A_max_ and Gs_max_ were done immediately after collecting the samples using an LC Pro Console Photosynthesis meter (model EN11 ODB, ADC Bioscientific Ltd., UK) between 09:30 h and 12:30 h (solar noon). The WUEi was determined as the ratio of A_max_ and Gs_max_ and expressed as µmol mol^-1^. For the measurement of leaf water potential (Ψ), we used a pressure chamber (Model 1000, PMS Instrument Co., Corvallis, Ore.). Measurements of Ψ_dawn_ for each species in each fragment started at 04.30 h and finished before sunrise, while Ψ_noon_ was generally measured between 12.30 h to 13.30 h. We measured seed mass, also following Pérez-Harguindeguy et al. (2013). For this, we collected 10 to 20 seeds from the selected trees, dried them in an oven at 80°C for 48 hours, and weighed them to record seed mass.

### Statistical analysis

Species composition or the abundance or relative ecological importance of the tree species in each forest fragment was expressed by the importance value index (IVI) (Curtis and McIntosh, 1951). Relative IVI for each species was calculated as the average of the values for relative basal area, relative density, and relative frequency. Stem biomass was obtained by using the equation given by King et al. (2006) as:


$$Stem\;biomass\;=\;0.5\;\times \left(\pi /4\right)\;\times \;WSG\;\times \;{\left(DBH\right)}^{2}\times \;H$$

where, 0.5 is the form factor, defined as the ratio of stem volume to the volume of a cylinder with the height (H, m) and diameter at breast height (DBH, cm) of the tree. We validated the estimates from this equation against those obtained by using species-specific as well as multi-specific allometric equations relating to destructively measured tree biomass and the CBH, for TDF tree species, as well as the actually measured biomass of harvested trees (Chaturvedi et al., 2010). We also compared the estimates of the stem biomass of two dominant species obtained using a CBH-based equation and observed that the estimate from the WSG-based equation was closer to the directly measured biomass (R^2 ^= 0.97, P< 0.001) compared to the CBH-based equation (R^2 ^= 0.83, P< 0.001). We calculated biomass per unit stem basal area, as well as per unit stand area. The biomass accumulation capacity for each tree species was calculated as the rate of change in biomass per unit basal area.

The statistical analyses were done in R version 3.6.3 (R Core Team, 2023). We used likelihood ratio tests (LRTs) for testing interactions and main effects. We also used Wald tests for evaluating the parameter estimates. The plasticity in functional traits was calculated for each species in each forest fragment by using the formula:


$$Trait\;plasticity=(\frac{Highest\;trait\;value-Lowest\;trait\;value}{Highest\;trait\;value})\times 100$$

The data normality was checked using the Shapiro–Wilk test and the data exhibiting non-normal distribution (*viz.*, CC, LDMC, LNC, Gs_max_, Ψ_dawn_, Ψ_noon_, Chl, HTDBH, CDDBH, and CCDBH) were log-transformed before statistical analysis. We performed one-way ANOVA for observing differences in soil properties and other habitat features across the 45 forest fragments. We compared SMC, mean traits (*viz.*, WSG, QWsat, CC, SLA, RWC, LDMC, LNC, LPC, Gs_max_, A_max_, WUEi, Ψ_dawn_, Ψ_noon_, Chl, LL, LA, SDWT, HTDBH, CDDBH, and CCDBH), and the plasticity of traits (*viz.*, ΔWSG, ΔQWsat, ΔCC, ΔSLA, ΔRWC, ΔLDMC, ΔLNC, ΔLPC, ΔGs_max_, ΔA_max_, ΔWUEi, ΔΨ_dawn_, ΔΨ_noon_, ΔChl, ΔLL, ΔLA, ΔSDWT, ΔHTDBH, ΔCDDBH, and ΔCCDBH) in the form of response variables with a linear mixed-effects model (nlme::lme, Pinheiro et al., 2016), where we defined site as a fixed effect and species as a random effect. We used an autoregressive moving average model for accounting for temporal autocorrelation. This model structure was required to appropriately account for the temporal autocorrelation caused by repeated measurements of SMC, biomass, and biomass accumulation capacity for the same individuals of each tree species. Pearson’s correlation coefficient was calculated on the average values of the functional traits for each species across the study sites by using the “Hmisc” (Harrell, 2017) and “xtable” (Dahl, 2016) packages.

We observed species groupings as functional types by using the 20 traits data for each species through PCA, hierarchical clustering, and partitioning clustering particularly through the k-means method using HCPC (hierarchical clustering on principal components). According to Kassambara (2017), HCPC is a robust tool for multivariate data analysis, where it allows three techniques (*viz*., hierarchical clustering, k-means partitioning, and PCA) in combination for extracting information from the data and summarizing results in the best possible format. The HCPC basically uses Euclidean distances for defining distances between individuals, while the hierarchical tree is constructed using Ward’s agglomeration method (Husson et al., 2010; Husson et al., 2011). According to Husson et al. (2010), the categories of cluster variables are represented by the categories of categorical variables. The HCPC generates a list of species groups in ascending order of P-value, which shows the order of impact of the categorical variables. HCPC also develops V-test values which are reported to be associated to the P-values (Husson et al., 2011). Moreover, the order of overrepresented positive V-test values exhibit categories of categorical variables according to their influence on the cluster variables (Husson et al., 2011). Our analysis was based on the average value of functional traits for all individuals of each species across the 45 forest fragments. We show the clusters of tree species in the form of a PCA biplot. The clustering of tree species was based on the shared set of functional traits. Based on the identity of functional traits in each cluster, we classified the total tree species into three functional types [*viz.*, low wood density high deciduous (LWHD), high wood density medium deciduous (HWMD), and high wood density low deciduous (HWLD)]. For this analysis, we used the R packages “FactoMineR” and “factoextra” (Kassambara and Mundt, 2016). We used functions prcomp() and PCA() in the “FactoMineR” package for PCA analysis.

The differences among functional types for the 20 functional traits were computed by Tukey HSD tests. The Tukey HSD tests were done by using the “multcomp” package (Hothorn et al., 2008). For plotting boxplots, we used the “ggplot2” package (Wickham, 2016).

We used random regression mixed models (RRMMs) with non-linear functions as both fixed (SMC) and random effects (functional types) for the analysis of trait plasticity data (Arnold et al., 2019) in order to analyze the average response of plasticity in functional traits across the functional types in the forest fragments. RRMM is a newly adapted approach in ecological data analysis which efficiently describes the response at the population level and also shows variations in the response at the individual level by using linear as well as non-linear functions as required (Morrissey and Liefting, 2016; Arnold et al., 2019). We selected the most appropriate model by comparing the Akaike information criterion (AIC) and likelihood-ratio test values.

We calculated community weighted means for the plasticity of 20 functional traits following Lavorel et al. (2008) for each forest fragment and, through step-wise regression, we identified the best environmental predictors of functional trait plasticity for each of the classified functional types in the forest region. For predicting plasticity in functional traits (response variables), the environmental predictors (explanatory variables) across the 45 forest fragments included soil physico-chemical properties, patch size, patch perimeter, edge distance, and mortality index. Further, we treated plasticity in functional traits as explanatory variables for identifying the best predictors of stem density, species richness, and biomass accumulation capacity across the 45 forest fragments for the three functional types and also for the tree species of whole forest region. For step-wise regression, we used the R packages “tidyverse” (Wickham et al., 2019), “caret” (Kuhn et al., 2016), and “leaps” (Lumley and Miller, 2009). We used the stepAIC() function in the “MASS” package (Venables and Ripley, 2002) for choosing the best model by AIC (Burnham and Anderson, 2002). The multicollinearity between predictor variables in the regression models was checked with generalized variation inflation factor (GVIF) by using vif() in the “car” package (Fox and Weisberg, 2019).

We performed redundancy analysis (RDA) to assess the response of plasticity in functional traits to environmental factors and to check whether such responses were specific to SMC. To account for relatedness to SMC, we included SMC across the forest fragments as a covariate (i.e., in RDA, the plasticity in functional traits are the standardized response variables, the environmental factors including soil physico-chemical properties, fragment properties, and mortality index are explanatory variables, while the SMC is the covariate). We conducted a Monte Carlo permutation test based on 999 random permutations for testing the significance of the eigenvalues of the canonical axes and the marginal and conditional significance of explanatory variables. For RDA, we used the functions rda() and anova.cca(), respectively, both from the package “vegan” (Oksanen et al., 2019). We also checked multicollinearity and dropped the traits with vif > 10 (Borcard et al., 2018) from the final RDA. For plotting RDA projections, we used the “ggvegan” package (Simpson, 2019).

For understanding the influence of edge distance on the plasticity of functional traits, we performed multiple regression analysis and predicted the community weighted means of the plasticity of functional traits (response variable) along the edge distance gradient across the forest fragments for the three functional types (explanatory variables). For this analysis, we used the functions lm() from the “stats” package (Field et al., 2012) for fitting linear models, stat_cor() from the “ggpubr” package (Kassambara, 2020) for extracting P-values and R-values, and “ggplot2” (Wickham, 2016) for plotting.

## Results

### Soil and vegetation properties in forest fragments

**Supplementary Table S2** shows the average value of soil properties, and **Supplementary Table S3** summarizes the average value of SMC under the tree canopy and the mean value of the 20 functional traits for the 47 tree species across the 45 forest fragments. Based on ANOVA results, the differences across fragments for soil and vegetation properties were statistically significant (**Table 2**). Across the 45 forest fragments, we recorded 47 tree species, where 19 fragments were dominated by *Shorea robusta*, 6 fragments by *Acacia catechu*, 6 fragments by *Lagerstroemia parviflora*, 5 fragments by *Tectona grandis*, 5 fragments by *Buchanania cochinchinensis*, and 3 fragments by *Terminalia tomentosa* (**Supplementary Table S1**).

**Table 2:** Summary of analysis of variance (ANOVA) on habitat properties across the 45 forest fragments.

Habitat properties	Sum Sq	F value	Pr(>F)
Soil moisture content	406.590	114.09	1.124e-13 ***
Organic carbon	3.429	15.857	2.589e-04 ***
Total nitrogen	0.002	4.831	3.339e-02 *
Total phosphorus	0.005	5.413	2.156e-02 *
Clay	517.570	301.86	2.200e-16 ***
Silt	8.722	4.704	4.060e-02 *
Sand	391.910	42.991	5.661e-08 ***
Bulk density	0.004	5.242	2.713e-02 *
Patch size	12,456.3	46.254	2.500e-08 ***
Patch perimeter	27.592	99.935	8.718e-13 ***
Edge distance	377,983	211.26	2.200e-16 ***
Tree density	6,913.8	134.00	8.473e-15 ***
Species richness	635.94	111.14	1.697e-13 ***
Tree recruitment	9.393	13.234	7.316e-04 ***
Tree mortality	2.125	5.406	2.422e-02 *
Tree mortality index	220.76	13.539	6.463e-04 ***

Residual df = 43. nsP > 0.05, *P< 0.05, ***P< 0.001.

Based on the linear mixed-effects result, we observed significant variations across the fragments for mean values of SMC under the tree canopy and for all functional traits, except for QWsat and CCDBH (**Table 3**). The results of Pearson’s correlation showed significant positive relationships of WSG with CC, SLA, and LL; QWsat with WUEi and Ψ_dawn_; CC with RWC and LL; SLA with RWC, and both SLA and RWC with LNC, LPC, Gs_max_, A_max_, and Chl; LDMC with WUEi; LNC with LPC, and both LNC and LPC with Gs_max_, A_max_, Ψ_dawn_, Chl, and CDDBH; Gs_max_ with A_max_, and both Gs_max_ and A_max_ with Chl and CDDBH; Ψ_dawn_ with Chl, HTDBH, and CCDBH; Ψ_noon_ with SDWT; Chl with CDDBH; and CDDBH with CCDBH (**Supplementary Table S4**). However, the correlation was significantly negative for QWsat with WSG, CC, Ψ_noon_, and LL; CC with Ψ_dawn_ and LA; RWC with LDMC and LA; LDMC with LNC, LPC, Gs_max_, A_max_, and Chl; LNC with LA; LPC and Gs_max_ with WUEi; WUEi with Chl and CDDBH; and Ψ_dawn_ with LL (**Supplementary Table S4**). We also analyzed site-wise differences in the plasticity of functional traits based on linear mixed-effects models and observed that among the 20 functional traits, significant differences were detected for plasticity in only seven functional traits (*viz.*, ΔQWsat, ΔLPC, ΔΨ_dawn_, ΔΨ_noon_, ΔChl, ΔLA, and ΔHTDBH) (**Table 3**). Therefore, to get better information about the impact of site conditions on the plasticity of functional traits, we grouped the tree species into functional types based on the selected functional traits.

**Table 3:** Summary of linear mixed-effect models showing variations of soil moisture content below tree canopy, functional traits, and plasticity of functional traits for 47 tree species across the 45 forest fragments, where we defined the site as a fixed effect and species as a random effect.

Variable	Functional traits (mean)			Functional traits (plasticity)		
	Std. Error	*t*-value	*P*-value	Std. Error	*t*-value	*P*-value
Soil moisture content	0.120	-10.059	0.000 ***	NA	NA	NA
Wood specific gravity	0.002	3.734	0.000 ***	106.745	-0.431	0.667ns
Saturated stem water potential	5.069	-0.746	0.4562ns	110.266	2.399	0.036 *
Canopy cover intensity	0.803	2.216	0.0271 *	57.954	0.906	0.365ns
Specific leaf area	0.638	4.059	0.000 ***	135.033	0.436	0.663ns
Relative water content	0.926	-2.142	0.0456 *	390.870	0.354	0.724ns
Leaf dry matter content	0.172	-3.819	0.000 ***	75.577	-0.274	0.784ns
Leaf nitrogen content	0.014	-12.314	0.000 ***	201.672	0.538	0.591ns
Leaf phosphorus content	0.002	-14.220	0.000 ***	158.886	2.323	0.039 *
Maximum stomatal conductance	0.002	2.251	0.02114 *	55.867	0.134	0.894ns
Maximum photosynthetic rate	0.075	-7.648	0.000 ***	230.303	-0.708	0.480ns
Intrinsic water use efficiency	0.381	-6.361	0.000 ***	77.855	0.405	0.686ns
Leaf water potential at dawn	0.005	14.430	0.000 ***	16.477	-4.009	0.000 ***
Leaf water potential at noon	0.010	10.086	0.000 ***	25.919	-2.186	0.046 *
Chlorophyll content	0.011	8.178	0.000 ***	40.149	-2.095	0.043 *
Leaf life-span	1.016	9.486	0.000 ***	3.790	-0.863	0.389ns
Leaf area	1.236	4.501	0.000 ***	43.854	-2.142	0.047 *
Seed mass	0.004	-5.084	0.000 ***	367.694	0.229	0.819ns
Height : DBH ratio	0.002	2.382	0.0176 *	265.011	-2.060	0.049 *
Canopy depth:DBH ratio	0.002	2.794	0.0054 **	114.492	-0.039	0.969ns
Canopy cover:DBH ratio	0.005	0.452	0.6516ns	35.79878	0.731	0.465ns

Residual df = 525. nsP > 0.05, *P< 0.05, **P< 0.01, ***P< 0.001, NA = data not available.

### Species clustering based on functional traits

**Figure 2:**
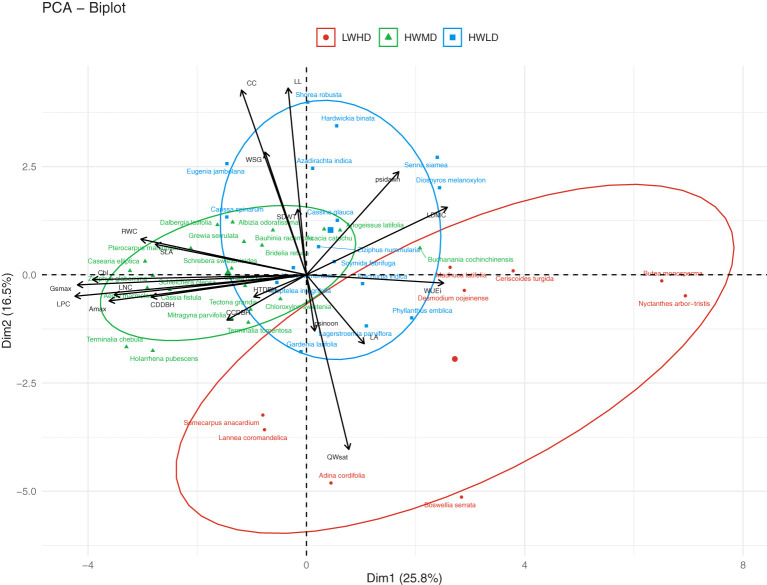
Ordination of the 47 tree species of tropical dry forests along the first and second PCA axes into three functional types (viz., LWHD, low wood density and high deciduous; HWMD, high wood density and medium deciduous; and HWLD, high wood density and low deciduous), resulting from PCA of their wood specific gravity (WSG), stem water storage capacity (QWsat), canopy cover intensity (CC), specific leaf area (SLA), relative water content (RWC), leaf dry matter content (LDMC), leaf nitrogen content (LNC), leaf phosphorus content (LPC), maximum saturated stomatal conductance (Gs_max_), maximum saturated photosynthesis (A_max_), intrinsic water use efficiency (WUEi), leaf water potential at dawn (Ψ_dawn_), leaf water potential at noon (Ψ_noon_), chlorophyll content (Chl), leaf life-span (LL), leaf area (LA), seed mass (SDWT), ratio of total height and diameter at breast height (HTDBH), ratio of crown depth and diameter at breast height (CDDBH), and ratio of crown cover and diameter at breast height (CCDBH).

**Figure 3:**
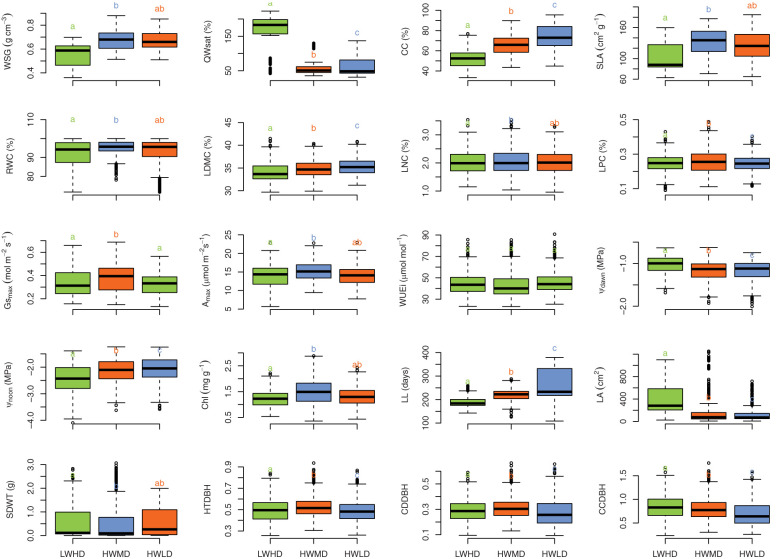
Boxplot showing the distribution of wood specific gravity (WSG), stem water storage capacity (QWsat), canopy cover intensity (CC), specific leaf area (SLA), relative water content (RWC), leaf dry matter content (LDMC), leaf nitrogen content (LNC), leaf phosphorus content (LPC), maximum saturated stomatal conductance (Gs_max_), maximum saturated photosynthesis (A_max_), intrinsic water use efficiency (WUEi), leaf water potential at dawn (Ψ_dawn_), leaf water potential at noon (Ψ_noon_), chlorophyll content (Chl), leaf life-span (LL), leaf area (LA), seed mass (SDWT), ratio of total height and diameter at breast height (HTDBH), ratio of crown depth and diameter at breast height (CDDBH), and ratio of crown cover and diameter at breast height (CCDBH) across three functional types (viz., LWHD, low wood density and high deciduous; HWMD, high wood density and medium deciduous; and HWLD, high wood density and low deciduous). Different letters and colors indicate significant differences (Tukey HSD test, *P<* 0.01) between functional types.

PCA and HCPC analysis generated the PCA biplot (**Figure 2**) which clearly explained the variability among 47 tree species on the basis of 20 functional traits. The eigenvalues of the first two PCA axes were 5.52 and 3.12, respectively. The individual variance explained by the first two PCA axes were 28.6% and 15.2%, respectively, while the two axes together accounted for 43.8% of the total multivariate variation during PCA. The first PCA axis exhibited stronger associations with LPC (R = 0.733), Gs_max_ (R = 0.700), and A_max_ (R = 0.625), while the second PCA axis indicated stronger correlations with CC (R = 0.764), LL (R = 0.753), and QWsat (R = 0.633). The HCPC separated the total 47 tree species into three separate clusters, as shown in **Figure 2**. Description of the quantitative variables of HCPC, including V-test, indicated that the species group in cluster 1 exhibited significant positive association with only QWsat (**Supplementary Table S5**). However, in cluster 2, the species group was observed to have significant positive influence of Gs_max_, A_max_, Chl, LPC, LNC, and RWC. For cluster 3, the species group exhibited significant positive influence for WUEi and LDMC (**Supplementary Table S5**). The functional traits describing cluster 1 are important features of species exhibiting low wood density and high deciduousness, while those traits describing species in cluster 2 are important features of high productivity, medium deciduousness, and high wood density. Additionally, the traits in cluster 3 are important features of species exhibiting long-lived leaves with dense wood. Therefore, the tree species in clusters 1, 2, and 3 were categorized into the three functional types as, low wood density high deciduous (LWHD), high wood density medium deciduous (HWMD), and high wood density low deciduous (HWLD) functional types, respectively. Based on the Tukey HSD test results, we observed significant differences among functional types for all functional traits except WUEi (**Figure 3**).

Across the 45 forest fragments, relative species richness among the three functional types indicated that 19.1% of tree species were represented by LWHD functional type, while 46.8% and 34.1% of tree species were associated with HWMD and HWLD functional types, respectively. Based on the IVI data, 8.7%, 47.9%, and 43.4% of tree cover across the forest fragments was represented by the LWHD, HWMD, and HWLD functional types, respectively. Further, we categorized the total 45 forest fragments into three groups based on fragment size, where group 1 represented the large forest fragments (> 40 ha area, 10 fragments, covering 644 ha forest area), group 2 represented the medium forest fragments (40 – 20 ha area, 14 fragments, covering 363 ha forest area), and group 3 represented the small forest fragments (< 20 ha area, 21 fragments, covering 172 ha forest area), and we observed the composition of tree cover by the classified functional types in these three categories of fragments. For the large fragments, we found 11.6%, 46.5%, and 41.9% of tree cover represented by LWHD, HWMD, and HWLD functional types, respectively. For the medium fragments, we found 9.1%, 47.0%, and 43.9% of tree cover represented by the LWHD, HWMD, and HWLD functional types, respectively, and for the small fragments, we recorded 5.4%, 50.1%, and 44.5% of tree cover represented by the LWHD, HWMD, and HWLD functional types, respectively. Additionally, regardless of functional type, along the declining edge distance gradient, we observed a 55.6% decline in species richness and a 71.1% decline in tree density.

### Influence of SMC on variations in functional traits

**Figure 4:**
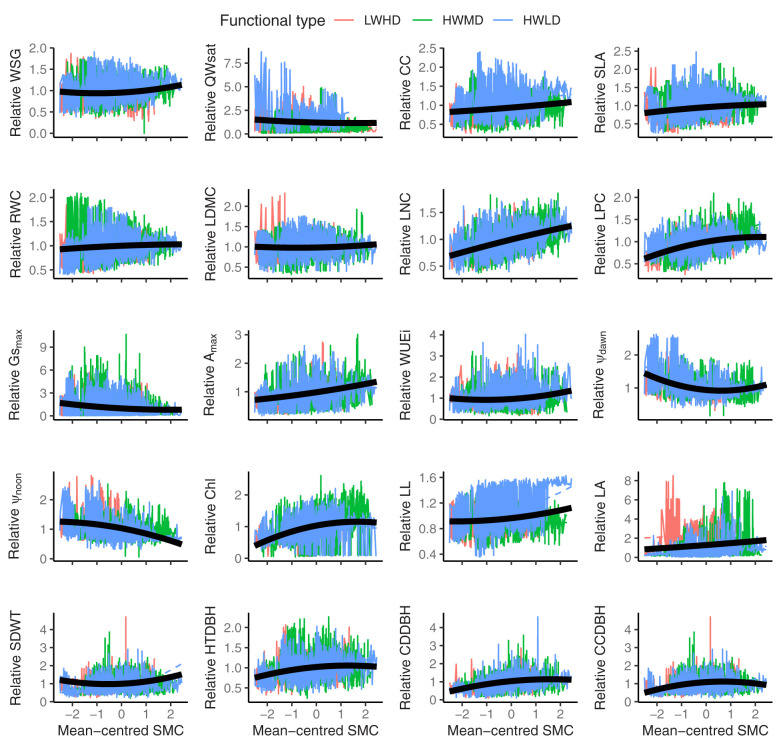
Relative variations in the 20 functional traits along mean-centered soil moisture content (SMC) across the three functional types [viz., 1 (LWHD, low wood density and high deciduous); 2 (HWMD, high wood density and medium deciduous); and 3 (HWLD, high wood density and low deciduous)]. Solid colored lines represent the raw data; the thick black line represents the quadratic regression model fit of the overall effect of mean-centered soil moisture content (the predicted average population-level reaction norm); and the dashed colored lines represent each functional type’s modeled reaction norms from the random regression mixed-effects model. WSG, wood specific gravity; QWsat, stem water storage capacity; CC, canopy cover intensity; SLA, specific leaf area; RWC, relative water content; LDMC, leaf dry matter content; LNC, leaf nitrogen content; LPC, leaf phosphorus content; Gs_max_, maximum saturated stomatal conductance; A_max_, maximum saturated photosynthesis; WUEi, intrinsic water use efficiency; Ψ_dawn_, leaf water potential at dawn; Ψ_noon_, leaf water potential at noon; Chl, chlorophyll content; LL, leaf life-span; LA, leaf area; SDWT, seed mass; HTDBH, ratio of total height and diameter at breast height; CDDBH, ratio of crown depth and diameter at breast height; CCDBH, ratio of crown cover and diameter at breast height. The best fitting model for SLA, Chl, LA, HTDBH, and CDDBH is model_4 (see **Table 4**), while for the other traits, the best fitting model is model_5 (see **Table 4**).

**Table 4** shows the results of mixed-effect models exhibiting the relationships between functional trait variations and soil moisture content (SMC), and **Figure 4** shows the trend of relative values of functional traits across populations of tree species in the three functional types due to changes in SMC. Based on AIC and maximum likelihood, the quadratic models explained the variations in functional traits more precisely compared to the linear models (**Table 4**). We observed that the plasticity response of the 16 functional traits were strongly described by the quadratic model which allows the random effect of functional type to vary in not only intercept and slope but also in curvature by fitting an additional quadratic random effect term (**Table 4**). However, for the remaining four traits (*viz*., RWC, LDMC, WUEi, and Ψ_noon_), we selected the model which specifies that the random component for the functional type can vary both in intercept and in slope (**Table 4**). We also found R^2^_C_ (conditional R^2^ that explains the proportion of variance accounted for by the random- and fixed-effects combined) was generally higher for quadratic models compared to the linear models, except for the variations in Gs_max_, Ψ_dawn_, Chl, HTDBH, and CCDBH, where the R^2^_C_ for linear models were higher compared to quadratic models (**Table 4**). Moreover, based on P-values, we observed significant associations of variations in functional traits with SMC for both linear as well as quadratic models. Additionally, we observed a few differences between linear and quadratic model trends along the SMC gradient. For instance, variations in WSG, LDMC, WUEi, Ψ_dawn_, and Ψ_noon_ for quadratic models exhibited significant positive associations with SMC, whereas their relationships were significantly negative based on linear models. Similarly, the relationships of variations in CC, RWC, Gs_max_, Chl, LL, HTDBH, CDDBH, and CCDBH were significantly negative for quadratic models, while the associations were significantly positive for linear models. For the variations in Qwsat, SLA, LNC, LPC, A_max_, LA, and SDWT, both quadratic as well as linear models exhibited similar trends, where all traits except Qwsat showed a significant positive trend along the SMC gradient (**Table 4**).

**Table 4:** Mixed effect modelling of the relationships between functional traits and soil moisture content (SMC), i.e., model_1<- lmer(relative_functionaltrait_plasticity ~ cSMC + (1|loc), REML = FALSE, data = data); model_2<- lmer(relative_functionaltrait_plasticity ~ poly(cSMC, 2, raw = T) + (1|loc), REML = FALSE, data = data); model_3<- lmer(relative_functionaltrait_plasticity ~ poly(cSMC, 2, raw = T) + (1|loc) + (1|functionaltype), REML = FALSE, data = data); model_4<- lmer(relative_functionaltrait_plasticity ~ poly(cSMC, 2, raw = T) + (1|loc) + (1+cSMC|functionaltype), REML = FALSE, data = data); and model_5<- lmer(relative_functionaltrait_plasticity ~ poly(cSMC, 2, raw = T) + (1loc) + (1 + cSMC + I(cSMC^2)|functionaltype), REML = FALSE, data = data), where cSMC is the mean centered SMC and loc denotes location based on SMC.

S. No.	Trait	Fixed effects (Estimate)													Random effects (Variance)				
		Intercept	Slope (linear)	Slope (quad)	AIC (linear)	AIC (quad)	logLik (linear)	logLik (quad)	R^2^_M_ (linear)	R^2^_M_ (quad)	R^2^_C_ (linear)	R^2^_C_ (quad)	P (linear)	P (quad)	loc	FT	cSMC	cSMC^2	RES
1.	WSG	1.014	-0.001	0.030	-688.4	-720.5	348.2	371.2	0.001	0.032	0.559	0.577	**<0.05**	**<0.001**	2.89e-02	2.80e-05	5.39e-04	8.28e-05	2.30e-02
2.	QWsat	0.988	-0.016	-0.051	5605.6	5310.6	-2798.8	-2644.3	0.002	0.028	0.071	0.164	**<0.05**	**<0.01**	1.13e-02	6.11e-02	6.01e-04	1.24e-03	3.59e-01
3.	CC	1.000	0.031	-0.016	-52.40	-384.9	30.20	203.4	0.016	0.047	0.324	0.422	**<0.001**	**<0.05**	1.61e-02	3.81e-03	3.18e-04	4.78e-04	3.70e-02
4.	SLA	0.999	0.081	0.031	550.3	498.0	-271.2	-238.0	0.064	0.034	0.131	0.154	**<0.001**	**<0.05**	4.69e-03	8.75e-04	1.22e-03	4.08e-04	6.45e-02
5.	RWC	1.004	0.018	-0.007	-2289.8	-2293.9	1148.9	1152.0	0.012	0.108	0.619	0.619	**<0.001**	**<0.05**	1.91e-02	NA	NA	NA	1.20e-02
6.	LDMC	1.003	-0.030	0.006	-2127.8	-2129.2	1067.9	1069.6	0.024	0.024	0.747	0.746	**<0.001**	**<0.05**	2.68e-02	NA	NA	NA	5.89e-03
7.	LNC	0.993	0.117	0.012	-598.0	-640.3	303.0	331.1	0.219	0.156	0.418	0.419	**<0.001**	**<0.05**	1.29e-02	5.70e-04	2.17e-04	2.48e-04	3.53e-02
8.	LPC	0.989	0.121	0.007	-396.3	-411.7	202.1	216.9	0.217	0.218	0.417	0.430	**<0.001**	**<0.05**	1.36e-02	7.83e-06	2.43e-04	1.65e-04	3.85e-02
9.	Gs_max_	0.990	0.192	-0.030	196.3	121.8	-94.10	-49.90	0.370	0.363	0.442	0.428	**<0.001**	**<0.05**	5.02e-03	2.10e-03	2.16e-05	1.87e-04	5.58e-02
10.	A_max_	0.998	0.102	0.003	-1349.3	-1474.8	678.6	748.4	0.217	0.133	0.365	0.393	**<0.001**	**<0.05**	8.70e-03	2.60e-03	7.60e-04	2.28e-05	2.71e-02
11.	WUEi	1.014	-0.118	0.041	269.9	195.6	-131.0	-89.80	0.164	0.207	0.560	0.566	**<0.001**	**<0.05**	3.08e-02	1.14e-04	2.14e-04	NA	3.76e-02
12.	Ψ_dawn_	1.008	-0.082	0.009	72.00	-93.50	-32.0	57.70	0.091	0.028	0.565	0.545	**<0.001**	**<0.05**	3.07e-02	3.08e-04	6.42e-04	1.27e-03	3.19e-02
13.	Ψ_noon_	1.001	-0.100	0.002	268.3	207.9	-130.2	-97.9	0.124	0.150	0.500	0.531	**<0.001**	**<0.05**	2.97e-02	1.79e-03	NA	NA	3.87e-02
14.	Chl	0.993	0.176	-0.001	1237.7	1124.8	-614.9	-551.4	0.255	0.201	0.339	0.306	**<0.001**	**<0.001**	6.80e-03	1.03e-03	5.55e-06	9.17e-04	7.94e-02
15.	LL	1.008	0.040	-0.014	574.2	257.4	-283.1	-117.7	0.020	0.052	0.454	0.525	**<0.001**	**<0.05**	2.98e-02	4.72e-03	2.46e-04	5.04e-04	3.93e-02
16.	LA	0.988	0.166	0.013	9187.1	9047.9	-4589.6	-4512.9	0.019	0.031	0.074	0.136	**<0.001**	**<0.01**	7.57e-02	9.79e-03	5.36e-02	3.90e-03	1.29e-00
17.	SDWT	1.070	0.125	0.336	9567.4	9404.4	-4779.7	-4691.2	0.009	0.090	0.273	0.422	**<0.001**	**<0.01**	4.07e-01	1.55e-03	3.59e-02	8.15e-02	1.19e-00
18.	HTDBH	0.998	0.088	-0.007	-827.5	-830.7	417.8	426.3	0.151	0.141	0.214	0.200	**<0.001**	**<0.01**	2.83e-03	1.41e-05	1.43e-06	4.60e-05	4.08e-02
19.	CDDBH	1.067	0.132	-0.062	1262.4	1068.0	-627.2	-523.0	0.176	0.221	0.267	0.268	**<0.001**	**<0.01**	2.54e-04	7.85e-03	6.00e-05	1.49e-03	8.38e-02
20.	CCDBH	0.992	0.124	-0.075	1627.9	1425.2	-809.9	-701.6	0.125	0.175	0.369	0.315	**<0.001**	**<0.001**	1.41e-02	4.36e-03	1.27e-07	4.75e-04	8.25e-02

model_1 is a linear model with the random effect (intercept) of (1|loc) to account for the spatial differences among the individual trees across which each functional type is represented; model_2 represents model_1 fitted with a quadratic fixed-effect of SMC; model_3 is a random intercepts only-linear mixed-effects model with an additional term of (1|functionaltype) in model_2; model_4 represents a replacement in the random component of model_3 by (1+cSMC|functionaltype) which specifies that the random component that the functional type can vary both in intercept and in slope; model_5 allows the random effect of functionaltype to vary in not only intercept and slope but also in curvature by fitting an additional quadratic random effect term in model_4. WSG, wood specific gravity (g cm^-3^); QWsat, stem water storage capacity (%); CC, canopy cover intensity (%); SLA, specific leaf area (cm^2^ g^-1^); RWC, relative water content (%); LDMC, leaf dry matter content (%); LNC, leaf nitrogen content (% dry weight); LPC, leaf phosphorus content (% dry weight); Gs_max_, maximum saturated stomatal conductance (mol m^-2^ s^-1^); A_max_, maximum saturated photosynthesis (µmol m^-2^ s^-1^); WUEi, intrinsic water use efficiency (µmol mol^-1^); Ψ_dawn_, leaf water potential at dawn (MPa); Ψ_noon_, leaf water potential at noon (MPa); Chl, chlorophyll content (mg g^-1^ fresh weight); LL, leaf life-span (days); LA, leaf area (cm^2^); SDWT, seed mass (g); HTDBH, ratio of total height and diameter at breast height; CDDBH, ratio of crown depth and diameter at breast height; CCDBH, ratio of crown cover and diameter at breast height; Interc (intercept); Slope coeff (slope coefficient); Quad coeff (quadratic coefficient); R^2^_M_ (marginal R^2^ that is the fit of the fixed-effects only); R^2^_C_ (conditional R^2^ that explains the proportion of variance accounted for by the random- and fixed-effects combined); FT (functional type); RES (residuals). Number of obs = 2,865; groups: LOC = 2133; FT = 3; df = 4 (linear model); df = 11 (quadratic model); df.resid = 2854. Significant P-values are in bold font. The models listed in the table are based on AIC and logLik, where the best fitting model for RWC and LDMC is model_2, for Ψ_noon_ is model_3, for WUEi is model_4, while for the other traits, the best fitting model is model_5. NA = not available.

### Environmental impact on plasticity of functional traits

**Figure 5:**
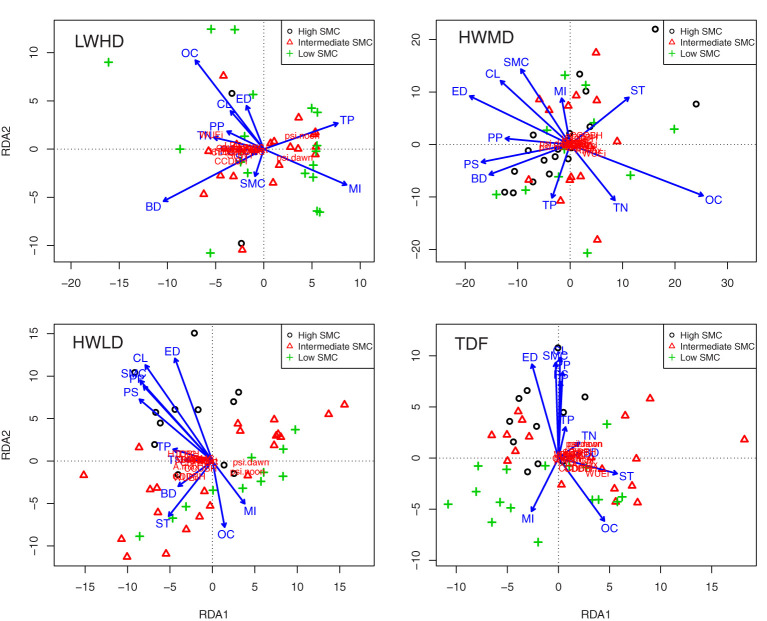
Projection of the community weighted means of the plasticity in functional traits and environmental parameters across the 45 forest fragments in RDA space, with soil moisture content (SMC) as covariate for low wood density and high deciduous species (LWHD), high wood density and medium deciduous species (HWMD), high wood density and low deciduous species (HWLD), and total tree species (TDF). WSG, wood specific gravity; QWsat, stem water storage capacity; CC, canopy cover intensity; SLA, specific leaf area; RWC, relative water content; LDMC, leaf dry matter content; LNC, leaf nitrogen content; LPC, leaf phosphorus content; Gs_max_, maximum saturated stomatal conductance; A_max_, maximum saturated photosynthesis; WUEi, intrinsic water use efficiency; Ψ_dawn_, leaf water potential at dawn; Ψ_noon_, leaf water potential at noon; Chl, chlorophyll content; LL, leaf life-span; LA, leaf area; SDWT, seed mass; HTDBH, ratio of total height and diameter at breast height; CDDBH, ratio of crown depth and diameter at breast height; CCDBH, ratio of crown cover and diameter at breast height; OC, soil organic carbon; TN, total nitrogen; TP, total phosphorus; CL, clay content; ST, silt content; SD, sand content; BD, bulk density; PS, patch size; PP, patch perimeter; ED, edge distance; HI, mortality index.

**Figure 5** shows projections of the community weighted means of plasticity in functional traits and environmental parameters for the three functional types, and for the TDF ecosystem, across the 45 forest fragments in RDA space, with soil moisture content (SMC) as a co-variate. Below, we describe the results of this analysis for the three functional types and for the TDF ecosystem.

(a) Low wood density high deciduous functional type (LWHD).

For the LWHD functional type, eigenvalue for the first and second constrained axes were 1,894.0 and 77.7, respectively, while the first and second constrained axes explained 89.0% and 3.7% of the total variation, respectively (pseudo-F = 2.86, P =< 0.01). The results of Pearson’s correlations between environmental factors and linear combinations of constraining variables along the RDA axes showed that the first axis was strongly correlated with bulk density (R = -0.477, P< 0.01) and total P (R = 0.352, P< 0.05), while the second axis showed a strong relationship with bulk density (R = -0.412, P< 0.01) and mortality index (R = -0.369, P< 0.05). The step-wise regression results relating to the community weighted means of plasticity in functional traits with environmental parameters, including soil properties and disturbances, indicated soil organic C, total P, clay, bulk density, patch size, and patch perimeter as the important variables, significantly explaining variances in the regression models, and all regression models exhibited the significant influence of environmental variables (**Supplementary Table S6**). The Pearson’s correlations of the plasticity in functional traits with the weighted sums of species scores along the RDA axes exhibited strong correlations of the first axis with ΔSLA (R = -0.983, P< 0.001), ΔQWsat (R = -0.972, P< 0.001), and ΔLDMC (R = -0.961, P< 0.001), whereas the second axis showed significant associations only with ΔCCDBH (R = -0.449, P< 0.01) and ΔWUEi (R = 406, P< 0.05).

(b) High wood density medium deciduous functional type (HWMD).

For the HWMD functional type, eigenvalue for the first and second constrained axes were 1,180.6 and 131.5, respectively, while the first and second constrained axes explained 74.7% and 8.3% of the total variation, respectively (pseudo-F = 2.79, P =< 0.01). The results of Pearson’s correlations between environmental factors and linear combinations of constraining variables along the RDA axes showed that the first axis was strongly correlated with organic C (R = 0.662, P< 0.001) and edge distance (R = -0.501, P< 0.001), while the second axis showed a strong relationship with silt content (R = 0.338, P< 0.05) and total phosphorus (R = -0.302, P< 0.05). The step-wise regression results relating to the community weighted means of plasticity in functional traits with environmental parameters, including soil properties and disturbances, indicated soil organic C and edge distance as the important variables significantly explaining variances in the regression models (**Supplementary Table S6**). The Pearson’s correlations of the plasticity in functional traits with the weighted sums of species scores along the RDA axes exhibited strong correlations of the first axis with ΔSLA (R = 970, P< 0.001), ΔQWsat (R = 964, P< 0.001), and ΔLDMC (R = 960, P< 0.001), whereas the second axis exhibited strong relationships with ΔCCDBH (R = 810, P< 0.001), ΔHTDBH (R = 758, P< 0.001), and ΔCDDBH (R = 730, P< 0.001).

(c) High wood density low deciduous functional type (HWLD).

For the HWLD functional type, eigenvalue for the first and second constrained axes were 1,719.9 and 159.1, respectively, while the first and second constrained axes explained 83.8% and 7.8% of the total variation, respectively (pseudo-F = 2.41, P =< 0.01). The results of Pearson’s correlations between environmental factors and linear combinations of constraining variables along the RDA axes showed that the first axis was strongly correlated with patch size (R = -0.602, P< 0.001) and patch perimeter (R = -0.568, P< 0.001), while the second axis showed a strong relationship with edge distance (R = 0.807, P< 0.001) and clay content (R = 0.751, P< 0.001). The step-wise regression results relating to the community weighted means of plasticity in functional traits with environmental parameters, including soil properties and disturbances, indicated clay, silt, patch size, and edge distance as the important variables significantly explaining variances in the regression models (**Supplementary Table S6**). The Pearson’s correlations of the plasticity in functional traits with the weighted sums of species scores along the RDA axes exhibited strong correlations of the first axis with ΔSLA (R = -0.983, P< 0.001), ΔGs_max_ (R = -0.975, P< 0.001), and ΔLDMC (R = -0.967, P< 0.001), whereas the second axis exhibited strong relationships with ΔWUEi (R = -0.726, P< 0.001), ΔCCDBH (R = -0.641, P< 0.001), and ΔCDDBH (R = -0.596, P< 0.001).

(d) Tropical dry forest (TDF).

For the total tree species combined (i.e., for all TDF species), eigenvalue for the first and second constrained axes was 635.1 and 84.5, respectively, while the first and second constrained axes explained 77.5% and 10.3% of the total variation, respectively (pseudo-F = 2.12, P = 0.056). The results of Pearson’s correlations between environmental factors and linear combinations of constraining variables along the RDA axes showed that the first axis was strongly correlated with silt content (R = 0.546, P< 0.001) and organic C (R = 0.428, P< 0.01), while the second axis showed stronger relationships with clay content (R = 0.942, P< 0.001) and edge distance (R = 0.871, P< 0.001). The step-wise regression results relating to the community weighted means of plasticity in functional traits with environmental parameters, including soil properties and disturbances, also indicated soil organic C, clay, silt, and edge distance as the important variables significantly explaining variances in the regression models for TDF species (**Supplementary Table S6**). The Pearson’s correlations of the plasticity in functional traits with the weighted sums of species scores along the RDA axes exhibited strong correlations of the first axis with ΔSLA (R = 976, P< 0.001), ΔQWsat (R = 954, P< 0.001), and ΔCC (R = 953, P< 0.001), whereas the second axis exhibited strong relationships with ΔΨ_dawn_ (R = 363, P< 0.05), ΔΨ_noon_ (R = 363, P< 0.05), and ΔCCDBH (R = -0.313, P< 0.05).

**Figure 6:**
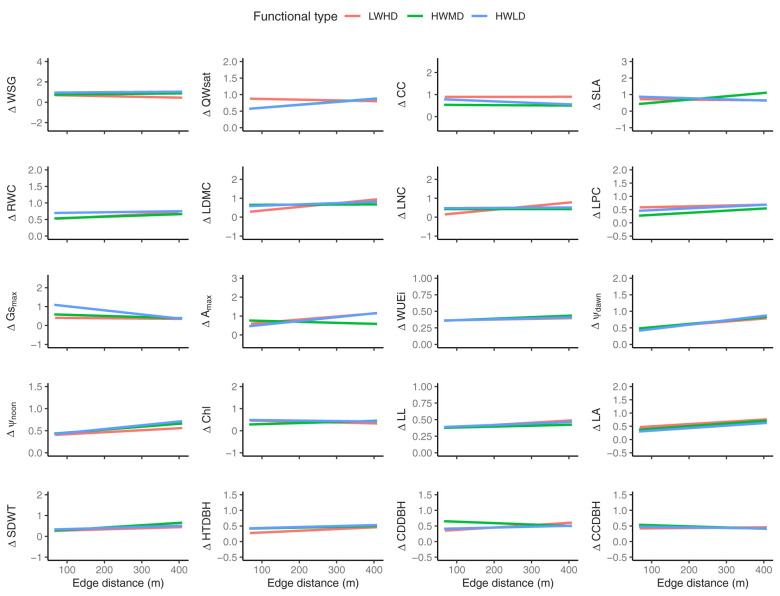
Multiple regression plots showing the trends of the three functional types for predicting community weighted means of the plasticity in functional traits along the edge distances across the 45 forest fragments. LWHD, low wood density and high deciduous; HWMD, high wood density and medium deciduous; HWLD, high wood density and low deciduous; WSG, wood specific gravity; QWsat, stem water storage capacity; CC, canopy cover intensity; SLA, specific leaf area; RWC, relative water content; LDMC, leaf dry matter content; LNC, leaf nitrogen content; LPC, leaf phosphorus content; Gs_max_, maximum saturated stomatal conductance; A_max_, maximum saturated photosynthesis; WUEi, intrinsic water use efficiency; Ψ_dawn_, leaf water potential at dawn; Ψ_noon_, leaf water potential at noon; Chl, chlorophyll content; LL, leaf life-span; LA, leaf area; SDWT, seed mass; HTDBH, ratio of total height and diameter at breast height; CDDBH, ratio of crown depth and diameter at breast height; CCDBH, ratio of crown cover and diameter at breast height. The “Δ” sign before the trait name represents plasticity.

The above results show that the plasticity in functional traits in the forest fragments is generally influenced by fragment properties, mainly edge distance, and the soil properties important for maximizing soil water availability, such as organic C and fine soil particles. However, for the functional trait plasticity of trees in the LWHD functional type, soil bulk density, total phosphorus, and disturbance index were highly significant. Therefore, we tried to observe the impact of edge distance on the plasticity of functional traits for the three functional types by analyzing multiple regression models. **Figure 6** shows multiple regression plots exhibiting trends of the three functional types for predicting the community weighted means of plasticity in functional traits along the edge distances across the 45 forest fragments. The summary of multiple regression model coefficients is listed in **Supplementary Table S7**. The results showed that the edge distance and functional types significantly explained the plasticity in functional traits for all functional traits, while their impacts were variable. Surprisingly, the trends of functional types explaining plasticity in functional traits along the edge distances were significantly different. For instance, the functional type LWHD exhibited significantly positive correlations with edge distance for explaining ΔWSG and ΔHTDBH, and significantly negative relationships with edge distance for explaining ΔLDMC, ΔLNC, ΔA_max_, ΔLL, ΔSDWT, ΔCDDBH, and ΔCCDBH. The functional type HWMD showed significantly negative relationships with edge distance for explaining ΔQWsat, ΔSLA, ΔRWC, ΔLPC, ΔWUEi, ΔΨ_dawn,_ ΔΨ_noon_, ΔChl, ΔLL, ΔLA, and ΔSDWT, while the functional type HWLD exhibited significantly positive associations with edge distance for explaining ΔCC and ΔGs_max_ and significantly negative relationships with edge distance for explaining ΔA_max_, ΔWUEi, ΔΨ_dawn,_ ΔΨ_noon_, and ΔLA.

### Influence of plasticity in functional traits on vegetation attributes

**Table 5** summarizes the results of the step-wise regressions relating plasticity in functional traits with vegetation attributes across the 45 forest fragments. Among the three functional types, stem density for the LWHD functional type was significantly explained by ΔSLA, ΔLPC, ΔΨ_noon,_ ΔChl, ΔLL, and ΔLA, and the model explained 53% of variations in the data matrix. For the HWMD functional type, ΔWSG, ΔRWC, ΔLPC, and ΔΨ_dawn_ significantly influenced stem density, where the model explained 61% of variations, while the HWLD functional type exhibited the significant impacts of ΔWSG, ΔRWC, ΔLDMC, ΔLPC, and ΔA_max_ in determining stem density, where the model explained 74% of variations. Additionally, we found that the stem density for TDF in general was significantly influenced by ΔWSG, ΔRWC, ΔLDMC, ΔLPC, ΔA_max_, ΔWUEi, ΔΨ_noon_, and ΔLA, and the model accounted for 79% of variance in the stem density.

We observed that the species richness of the LWHD functional type was better explained by the model containing ΔQWsat, ΔSLA, ΔRWC, ΔΨ_noon_, and ΔChl, where the model significantly accounted for 66% of variations (**Table 5**). For the species in the HWMD functional type, the model containing ΔWSG, ΔRWC, ΔΨ_dawn_, and ΔChl significantly explained 59% of variations in the data matrix, while for the HWLD functional type, the model exhibited the significant impacts of ΔWSG, ΔRWC, ΔLDMC, ΔLPC, ΔA_max_, and ΔCDDBH in determining species richness, and the model explained 73% of variations. Additionally, the TDF species in general were significantly affected by ΔSLA, ΔRWC, ΔChl, and ΔCDDBH, where the model explained 76% of variations.

**Table 5:** Summary of step-wise regressions relating to stem density, species richness, and biomass accumulation per unit basal area (BIOBA) with community weighted means of plasticity in functional traits across the tree species in 45 forest fragments (TDF) and for the species in the three functional types (viz., LWHD, low wood density and high deciduous; HWMD, high wood density and medium deciduous; HWLD, high wood density and low deciduous).

Vegetation attribute	Functional type	ANOVA	Variable	Estimate	Std. Error	*t*-value	*P*-value
Stem density	LWHD	RSE = 10.01, df = 26, *R^2 = ^* 0.677, Adj. *R^2 = ^* 0.528, *F* = 4.546, *P* = 0.000***, AIC = 189.89	Intercept	21.755	5.1370	4.235	0.000***
			ΔCC	1.865	1.0386	1.795	0.084ns
			ΔSLA	9.258	2.5239	3.669	0.001**
			ΔLNC	-0.725	0.3799	-1.908	0.068ns
			ΔLPC	-0.648	0.3098	-2.092	0.046*
			ΔΨ_dawn_	-0.254	0.1588	-1.602	0.121ns
			ΔΨ_noon_	0.373	0.1769	2.108	0.045*
			ΔChl	-1.414	0.3061	-4.620	0.000***
			ΔLL	3.026	0.9805	3.086	0.005**
			ΔLA	-0.814	0.3514	-2.317	0.029*
			ΔSDWT	-0.877	0.4401	-1.993	0.057ns
			ΔHTDBH	-0.460	0.2270	-2.026	0.053ns
			ΔCDDBH	0.523	0.2855	1.831	0.079ns
	HWMD	RSE = 9.045, df = 37, *R^2 = ^* 0.668, Adj. *R^2 = ^* 0.606, *F* = 10.66, *P* = 0.000***, AIC = 205.39	Intercept	37.531	5.9525	6.305	0.000***
			ΔWSG	1.302	0.3686	3.532	0.001**
			ΔCC	-1.197	0.6564	-1.824	0.076ns
			ΔRWC	-3.304	0.4876	-6.776	0.000***
			ΔLPC	0.550	0.2056	2.675	0.011*
			ΔWUEi	-0.090	0.0692	-1.302	0.201ns
			ΔΨ_dawn_	-0.266	0.0869	-3.054	0.004**
			ΔSDWT	0.316	0.1573	2.011	0.052ns
	HWLD	RSE = 7.304, df = 33, *R^2 = ^* 0.807, Adj. *R^2 = ^* 0.743, *F* = 12.56, *P* = 0.000***, AIC = 189	Intercept	27.330	4.1824	6.535	0.000***
			ΔWSG	1.330	0.3987	3.337	0.002**
			ΔQWsat	1.027	0.5946	1.728	0.093ns
			ΔSLA	-1.496	1.1075	-1.351	0.186ns
			ΔRWC	-2.341	0.5131	-4.561	0.000***
			ΔLDMC	2.426	0.7097	3.418	0.002**
			ΔLPC	0.504	0.2179	2.314	0.027*
			ΔA_max_	-0.582	0.1797	-3.239	0.003**
			ΔWUEi	-0.110	0.0674	-1.633	0.112ns
			ΔLA	-0.249	0.1536	-1.623	0.114ns
			ΔHTDBH	0.129	0.0821	1.567	0.127ns
			ΔCDDBH	-0.147	0.0916	-1.608	0.117ns
	TDF	RSE = 6.632, df = 35, *R^2 = ^* 0.831, Adj. *R^2 = ^* 0.788, *F* = 19.18, *P* = 0.000***, AIC = 178.96	Intercept	23.698	6.4034	3.701	0.000***
			ΔWSG	1.648	0.5422	3.039	0.004**
			ΔRWC	-3.365	0.6859	-4.906	0.000***
			ΔLDMC	3.043	0.8237	3.694	0.000***
			ΔLPC	1.104	0.2693	4.098	0.000***
			ΔA_max_	-0.547	0.2079	-2.631	0.013*
			ΔWUEi	-0.242	0.0851	-2.842	0.007**
			ΔΨ_dawn_	-0.265	0.1446	-1.832	0.076ns
			ΔΨ_noon_	0.354	0.1602	2.210	0.034*
			ΔLA	-0.757	0.2715	-2.788	0.009**
Species richness	LWHD	RSE = 2.672, df = 26, *R^2 = ^* 0.765, Adj. *R^2 = ^* 0.657, *F* = 7.054, *P* = 0.000***, AIC = 86.84	Intercept	6.895	1.4185	4.860	0.000***
			ΔWSG	0.324	0.1674	1.935	0.064ns
			ΔQWsat	-1.103	0.4967	-2.221	0.035*
			ΔSLA	4.107	0.7541	5.446	0.000***
			ΔRWC	-1.042	0.2952	-3.532	0.002**
			ΔLNC	-0.146	0.0998	-1.464	0.155ns
			ΔΨ_noon_	0.147	0.0456	3.231	0.003**
			ΔChl	-0.309	0.0817	-3.780	0.000***
			ΔLL	0.492	0.3351	1.469	0.154ns
			ΔLA	-0.147	0.0957	-1.536	0.137ns
			ΔSDWT	-0.165	0.1159	-1.426	0.166ns
			ΔHTDBH	-0.087	0.0622	-1.398	0.174ns
			ΔCDDBH	0.152	0.0747	2.035	0.052ns
	HWMD	RSE = 2.879, df = 38, *R^2 = ^* 0.643, Adj. *R^2 = ^* 0.586, *F* = 11.4, *P* = 0.000***, AIC = 101.58	Intercept	14.064	1.8883	7.448	0.000***
			ΔWSG	0.316	0.0971	3.257	0.002**
			ΔRWC	-0.774	0.1455	-5.319	0.000***
			ΔΨ_dawn_	-0.126	0.0266	-4.720	0.000***
			ΔChl	-0.083	0.0410	-2.027	0.049*
			ΔSDWT	0.078	0.0461	1.690	0.099ns
			ΔCCDBH	-0.035	0.0215	-1.629	0.112ns
	HWLD	RSE = 2.337, df = 34, *R^2 = ^* 0.789, Adj. *R^2 = ^* 0.727, *F* = 12.75, *P* = 0.000***, AIC = 85.8	Intercept	12.260	1.3369	9.170	0.000***
			ΔWSG	0.536	0.1271	4.216	0.000***
			ΔSLA	-0.662	0.3521	-1.881	0.069ns
			ΔRWC	-0.579	0.1628	-3.557	0.001**
			ΔLDMC	0.692	0.2250	3.077	0.004**
			ΔLPC	0.249	0.0599	4.160	0.000***
			ΔA_max_	-0.179	0.0551	-3.259	0.003**
			ΔWUEi	-0.038	0.0215	-1.774	0.085ns
			ΔLA	-0.079	0.0488	-1.637	0.111ns
			ΔHTDBH	0.051	0.0262	1.928	0.062ns
			ΔCDDBH	-0.071	0.0291	-2.453	0.019*
	TDF	RSE = 2.2, df = 35, *R^2 = ^* 0.807, Adj. *R^2 = ^* 0.758, *F* = 16.36, *P* = 0.000***, AIC = 79.66	Intercept	16.289	2.1117	7.714	0.000***
			ΔSLA	1.986	0.4214	4.715	0.000***
			ΔRWC	-1.007	0.2185	-4.611	0.000***
			ΔLNC	0.129	0.0906	1.427	0.163ns
			ΔA_max_	-0.109	0.0700	-1.570	0.125ns
			ΔWUEi	-0.053	0.0309	-1.731	0.092ns
			ΔΨ_dawn_	-0.092	0.0460	-2.005	0.053ns
			ΔChl	-0.259	0.0722	-3.587	0.001**
			ΔLA	-0.108	0.0852	-1.272	0.212ns
			ΔCDDBH	-0.072	0.0312	-2.311	0.027*
BIOBA	LWHD	RSE = 0.035, df = 31, *R^2 = ^* 0.415, Adj. *R^2 = ^* 0.283, *F* = 3.147, *P* = 0.012*, AIC = 254.85	Intercept	0.044	0.0158	2.798	0.008**
			ΔSLA	0.027	0.0064	4.243	0.000***
			ΔRWC	-0.006	0.0034	-1.866	0.071ns
			ΔA_max_	-0.002	0.0011	-1.860	0.072ns
			ΔChl	-0.002	0.0010	-2.453	0.020*
			ΔSDWT	-0.003	0.0012	-2.119	0.042*
			ΔHTDBH	-0.001	0.0006	-1.767	0.087ns
			ΔCDDBH	0.001	0.0008	1.667	0.106ns
	HWMD	RSE = 0.015, df = 36, *R^2 = ^* 0.419, Adj. *R^2 = ^* 0.291, *F* = 3.252, *P* = 0.007**, AIC = 371.49	Intercept	0.053	0.0104	5.164	0.000***
			ΔWSG	0.001	0.0006	2.341	0.025*
			ΔQWsat	0.002	0.0015	1.284	0.207ns
			ΔCC	-0.002	0.0013	-1.523	0.136ns
			ΔRWC	-0.003	0.0009	-2.742	0.009**
			ΔGs_max_	-0.0003	0.0002	-1.405	0.168ns
			ΔΨ_dawn_	-0.0002	0.0001	-1.759	0.087ns
			ΔSDWT	0.001	0.0002	2.203	0.034*
			ΔCDDBH	-0.0002	0.0001	-1.529	0.135ns
	HWLD	RSE = 0.011, df = 34, *R^2 = ^* 0.690, Adj. *R^2 = ^* 0.599, *F* = 7.572, *P* = 0.000***, AIC = 400.28	Intercept	0.035	0.0061	5.586	0.000***
			ΔWSG	0.002	0.0005	2.808	0.008**
			ΔQWsat	-0.003	0.0008	-3.423	0.002**
			ΔLNC	0.001	0.0003	1.420	0.165ns
			ΔLPC	0.001	0.0003	4.129	0.000***
			ΔChl	-0.001	0.0002	-2.180	0.036*
			ΔLL	-0.001	0.0006	-1.816	0.078ns
			ΔSDWT	0.001	0.0002	1.849	0.073ns
			ΔHTDBH	0.0002	0.0001	1.803	0.080ns
			ΔCDDBH	-0.0002	0.0001	-1.698	0.099ns
			ΔCCDBH	-0.0004	0.0001	-3.381	0.002**
	TDF	RSE = 0.012, df = 38, *R^2 = ^* 0.594, Adj. *R^2 = ^* 0.529, *F* = 9.261, *P* = 0.000***, AIC = 388.89	Intercept	0.046	0.0112	4.135	0.000***
			ΔQWsat	-0.003	0.0016	-2.084	0.044*
			ΔSLA	0.004	0.0028	1.465	0.151ns
			ΔRWC	-0.005	0.0011	-4.462	0.000***
			ΔLDMC	0.003	0.0016	1.968	0.056ns
			ΔLPC	0.001	0.0005	1.608	0.116ns
			ΔLL	-0.003	0.0011	-2.299	0.027*

WSG, wood specific gravity (g cm^-3^); QWsat, stem water storage capacity (%); CC, canopy cover intensity (%); SLA, specific leaf area (cm^2^ g^-1^); RWC, relative water content (%); LDMC, leaf dry matter content (%); LNC, leaf nitrogen content (% dry weight); LPC, leaf phosphorus content (% dry weight); Gs_max_, maximum saturated stomatal conductance (mol m^-2^ s^-1^); A_max_, maximum saturated photosynthesis (µmol m^-2^ s^-1^); WUEi, intrinsic water use efficiency (µmol mol^-1^); Ψ_dawn_, leaf water potential at dawn (MPa); Ψ_noon_, leaf water potential at noon (MPa); Chl, chlorophyll content (mg g^-1^ fresh weight); LL, leaf life-span (days); LA, leaf area (cm^2^); SDWT, seed mass (g); HTDBH, ratio of total height and diameter at breast height; CDDBH, ratio of crown depth and diameter at breast height; CCDBH, ratio of crown cover and diameter at breast height. The “Δ” sign represents plasticity. nsP > 0.05, *P< 0.05, **P< 0.01, ***P< 0.001.

Biomass accumulation capacity for the species belonging to the LWHD functional type accounted for the significant contribution of ΔSLA, ΔChl, and ΔSDWT in registering 28% of the variations in the data matrix, while HWMD showed the significant contributions of ΔWSG, ΔRWC, and ΔSDWT in explaining 29% of the variations in biomass accumulation capacity. The HWLD functional type exhibited the significant influences of ΔWSG, ΔQWsat, ΔLPC, ΔChl, and ΔCCDBH for accounting for 60% of the variations in biomass accumulation capacity, while the TDF species in general were significantly affected by ΔQWsat, ΔRWC, and ΔLL, where the model explained 53% variations in the data matrix (**Table 5**).

## Discussion

### Variations in vegetation properties

Our forest fragments exhibited significant variations in soil properties and vegetation structure, where edge distance was the dominant factor determining these variations. Similar studies in Amazonian forest fragments have reported a wide array of ecological changes over a timespan of more than three decades, and the main driver of forest fragment dynamics in these studies was also the edge distance (see Laurance et al., 2011). Based on the synthesis of studies in Amazonian forest fragments, Laurance et al. (2011) suggested that the fragments are highly sensitive to external disturbances, and even a small change in external land-management activities may shift the fragment ecosystems in different directions. In our study, the forest fragments were dominated by important timber trees (*viz.*, *Shorea robusta*, *Tectona grandis*, and *Terminalia tomentosa*), as well as the trees commercially utilized for non-timber forest products (*viz.*, *Acacia catechu*, *Buchanania cochinchinensis*, and *Lagerstroemia parviflora*). Therefore, these fragments were continuously exposed to anthropogenic disturbances of various types by the local human population (see Chaturvedi et al., 2012; Chaturvedi et al., 2017), leading to significant alterations in the structure and function of vegetation.

### Variations in functional traits

We observed significant differences among tree species for SMC measured under their crown, while the variations for 20 functional traits selected for this study were also significant across species and fragments. However, across the fragments, functional trait plasticity exhibited significant differences for less than half of the selected traits. This result was surprising, as we had plenty of reasons for getting significant species-wise differences in plasticity of functional traits, for the selected traits. For instance, among the probable reasons, our study sites have been reported to exhibit heterogeneous resource conditions and experience dry periods with very little precipitation for a major part of the year (Chaturvedi et al., 2011a; Chaturvedi et al., 2021). During dry periods, SMC under the tree crown is mainly determined by composition of fine soil particles and soil organic carbon on the upper layers of soil. Moreover, tropical trees differ in root biomass, rooting depth, rates of fine root production, symbiotic relationships, and aboveground attributes such as the rate of transpiration and leaf phenology, which also regulate the availability of soil water under the canopy (Chitra-Tarak et al., 2021). According to Roy and Singh (1994), the heterogeneity of water availability at our study sites also increases due to the presence of occasional topographic depressions, which accumulate litter biomass and organic carbon, acting as a sink for SMC and nutrients.

Hofhansl et al. (2021) suggested that in tropical forests, the mechanism responsible for functional trait variations are associated with multiple environmental drivers, and these variations potentially shift across latitudinal and altitudinal gradients. At the broad spatial scale, abiotic factors, for instance, temperature and precipitation, determine variations in functional traits (Taylor et al., 2017), while at the small spatial scale, variations are mainly due to competition among the co-existing species (Fauset et al., 2012). In our study, apart from the significant variations across forest fragments, the 20 functional traits were also strongly correlated across the fragments. He et al. (2019) suggested that functional traits are related to productivity and exhibit adaptation strategies to environmental conditions. Additionally, the functional traits also contain information about different mechanisms and processes, including phylogenetic signals, associations with physiological processes, and environmental constraints, at different scales (Kraft et al., 2007). The strong associations of functional traits across our forest fragments exhibit mutual co-ordination for minimizing the effects of drought and for increasing their efficiency to resist disturbances. We observed strong positive correlations of Qwsat with WUEi and Ψ_dawn_, while WUEi was positively related with LDMC, and Ψ_dawn_ was positively correlated with LNC, LPC, Chl, HTDBH, and CCDBH. These relationships suggest that the trees with high Qwsat have adapted strategies for conserving water in order to minimize drought stress. These species are generally shallow rooted and unable to acquire ground water at greater depth, and therefore they exhibit high deciduousness and fast growth during a short favorable wet season in a year. These trees also have low WSG, conferring rapid water transport and storage (Poorter et al., 2019), although they are also at the risk of cavitation and mortality during severe drought. Further, we found significant positive correlations of WSG with CC, SLA, and LL. The associations of CC, SLA, and LL were also positive with RWC, while both SLA and RWC showed positive correlations with LNC, LPC, Gs_max_, A_max_, and Chl. These relationships indicate the characteristics of trees possessing long-lived leaves and better water transport system. Such kinds of associations exhibit strategies for increasing drought tolerance in tropical dry systems. Moreover, these species have high CC and greater transpiration rates; therefore, for maintaining growth and high productivity, they also need to have strong root systems with greater depth and wider horizontal expansion (Markesteijn et al., 2011; Méndez-Alonzo et al., 2012; Pérez-Ramos et al., 2013; Pineda-García et al., 2013; Poorter et al., 2019; Chitra-Tarak et al., 2021).

### Variations in functional types

Earlier studies have shown that a combination of functional traits can better explain the ecosystem process compared to a single trait (Swenson and Weiser, 2010; Kraft et al., 2015; Solé-Medina et al., 2022). Therefore, we categorized the tree species across the forest fragments into three functional types based on their dominant functional traits. The results showed differences across the three functional types regarding trait combinations, for instance, the functional type LWHD was represented by the functional traits Qwsat, Ψ_dawn_, LA, and CCDBH. HWMD was mostly associated with WSG, SLA, RWC, LNC, LPC, Gs_max_, A_max_, Chl, HTDBH, and CDDBH, and HWLD was represented by the functional traits CC, LDMC, WUEi, Ψ_noon_, LL, and SDWT. The assemblage of these functional traits suggests that the functional type LWHD adopts a drought-avoiding strategy, while HWLD and HWMD exhibit drought-tolerant strategies in these forest regions. Interestingly, the relative species richness of trees exhibiting a drought-tolerant strategy across the forest fragments was 80.9% (HWMD, 46.8%; HWLD, 34.1%), while only 19.1% (LWHD) of tree species accounted for drought-avoiding strategies. Additionally, the IVI data showed that 91.3% (HWMD, 43.4%; HWLD, 47.9%) of the tree cover at our study sites was accounted for by species with drought-tolerant strategies, while only 8.7% (LWHD) of the tree cover was represented by species exhibiting drought-avoiding strategies. Further, we compared the relative composition of tree cover or tree density exhibiting drought-avoiding vs drought-tolerant strategies in large (> 40 ha), medium (20 – 40 ha), and small (< 20 ha) sized fragments, and surprisingly, we observed a considerable decline in the relative tree cover of the functional type with drought-avoiding strategies with declining fragment size (large, 11.6% > medium, 9.1% > small, 5.4%), while there was a gradual increase in the relative tree cover of functional types exhibiting drought-tolerant strategies with the declining size of fragments (large, 88.4%< medium, 90.9%< small, 94.6%). Moreover, among the important habitat factors, we recorded a gradual decline in the average SMC (large, 15.7% > medium, 13.8% > small, 9.1%) and elevation in the disturbance index (large, 3.3< medium, 2.8< small, 5.8) due to the reduction of fragment size. Consequently, overall, there was extensive decline in species richness and tree density along the declining fragment size. Since the LWHD functional type generally possess a shallow root system and are at a risk of xylem embolism related mortality, it is very difficult for these species to survive in very low soil water conditions. Additionally, due to having lighter wood, these plants are highly favored as fuel wood and are extensively harvested by the local residents. On the other hand, trees belonging to the HWMD and HWLD functional types are generally large sized trees with deep root systems allowing them to acquire belowground water even during prolonged drought periods. Moreover, these groups contain valuable timber trees and are highly protected from illegal harvesting. These factors might be favoring the increasing dominance of the HWMD and HWLD functional types in smaller fragments as well.

Based on these observations, we suspected the confounding role of environmental factors on the dynamics of the composition of functional types and tried to do a more rigorous investigation about the impact of environmental factors on the variations in functional traits in the forest fragments. In a recent synthesis, Zambrano et al. (2019) also expressed a dire need to understand the functional trait variations related to biotic and abiotic stresses in the fragmented landscape and argued that the changes in abiotic factors in a fragmented landscape may shift the structure of competition in plant communities and may favor the development of more stress-tolerant species with greater phenotypic plasticity. In our study, the increasing tree cover of the functional types exhibiting drought-tolerant strategies indicates that these functional types are more stress-tolerant and exhibit higher variations in functional traits. In the following sections, we will discuss the important factors influencing plasticity in functional traits and the role of plasticity in functional traits in determining the structure and productivity of trees in the forest fragments.

(a) Influence of SMC on variations in functional traits.

During a study of the influence of variations in functional traits on species co-existence across contrasting climatic conditions, Pérez-Ramos et al. (2019) suggested that the variations in functional traits of individuals for acquiring light and soil resources is significantly associated with the differences in average fitness between species, while the trend of correlations may shift between different climatic conditions. We found significant associations of the variations in functional traits with variations in SMC measured under the tree crown for the three functional types across the forest fragments. However, these relationships were highly complex, since the associations were better represented by the quadratic models compared to the linear models. Generally, the linear model lacks flexibility for dealing with incomplete data sets and has less efficiency in capturing non-linear responses among the predictors; therefore, for exploring the complex effects of trait fitness among different individuals, linear models have not been suggested as the suitable approach (Pistón et al., 2019). Moreover, a few associations between linear and quadratic models exhibited differences in their trends, where the significantly positive relationships depicted by linear models were significantly negative by quadratic models, and vice-versa (**Table 4**).

According to Geber and Griffen (2003), the coefficients of linear models represent a quantity of directional selection and show whether the selection is favoring larger or smaller values of traits, while the coefficients of quadratic models are a measure of curvature in the trait–fitness association. In other words, within the range of phenotypic plasticity, given that the data show maximum fitness at the intermediate trait value, the negative quadratic coefficients exhibit a decelerating relationship between fitness and trait value, leading to a stabilizing selection for extreme trait plasticity, whereas the positive quadratic coefficients show an accelerating association, leading to a disruptive selection for the extreme trait plasticity (see Geber and Griffen, 2003). In our study, significantly negative quadratic coefficients were observed for the variations in Qwsat, CC, RWC, Gs_max_, Chl, LL, HTDBH, CDDBH, and CCDBH, whereas positive coefficients accounted for the variations in WSG, SLA, LDMC, LNC, LPC, A_max_, WUEi, Ψ_dawn_, Ψ_noon_, LA, and SDWT. These findings indicate that the increasing variations in Qwsat, CC, RWC, Gs_max_, Chl, LL, HTDBH, CDDBH, and CCDBH with elevation in SMC will lead to a stabilizing selection, whereas increasing variations in WSG, SLA, LDMC, LNC, LPC, A_max_, WUEi, Ψ_dawn_, Ψ_noon_, LA, and SDWT will lead to a disruptive selection for the tree species at our study sites. Our observations could be supported by earlier research findings where increasing variation in WUEi has been linked with a decline in soil water availability, leading to lower carbon assimilation and productivity (Donovan and Ehleringer, 1994; Miller et al., 2001), while CC, RWC, Gs_max_, Chl, LL, HTDBH, CDDBH, and CCDBH have been reported as the important indicators of the higher availability of soil water and nutrients, and increases in these trait values show increasing productivity (Chaturvedi et al., 2011a; Zhang et al., 2012). Moreover, increasing variations in WSG, SLA, LDMC, LNC, LPC, A_max_, Ψ_dawn_, Ψ_noon_, LA, and SDWT may require large investment of water and resources, which is not affordable in nutrient-poor soils with limited water availability.

(b) Influence of soil physico-chemical properties and disturbances on plasticity of functional traits.

Based on the RDA and step-wise regression results, we found that the plasticity in functional traits across forest fragments is generally determined by those environmental factors which are linked with the plant strategy for acquiring soil water; for instance, among soil properties, the dominant factors were particle size and soil organic carbon, although edge distance was also an important factor common for all functional types, except for the trees in LWHD functional type, where soil phosphorus and mortality index were stronger determining factors. Studies have reported that comparatively high deciduous species require more phosphorus, since it is required during the frequent turnover of leaves (Givnish, 2002; Condit et al., 2013). Our study sites have been reported to exhibit nutrient poor soil with high phosphorus deficiency (Roy and Singh, 1994; Chaturvedi, 2010), and the disturbance tends to increase with reducing fragment size. This might be the reason for the declining tree cover and species richness for the LWHD functional type in the smaller fragments. We also observed that ΔSLA, ΔQWsat, ΔLDMC, ΔCCDBH, and ΔWUEi for the LWHD functional type were more influenced by soil bulk density, total P, and disturbance index. Therefore, we can argue that the analysis of relationships of the plasticity of these functional traits with the observed ranges of phosphorus availability and drought stress could help in understanding the population dynamics of trees in the LWHD functional type at our study sites (see Condit et al., 2013).

The differences in plasticity of functional traits among species and populations generally indicate differences in selective pressure and limiting factors influencing variations in plasticity (van Kleunen and Fischer, 2005; Valladares et al., 2007). In our study, the multiple regression results exhibited significant influence of edge distance on the community weighted means of plasticity in functional traits. For the functional type exhibiting drought-avoiding strategy (i.e., LWHD), the plasticity in functional traits generally exhibited a significantly negative association with edge distance for the traits linked with the conservation of resources. However, for the functional types exhibiting drought-tolerant strategies (i.e., HWMD and HWLD), the plasticity in functional traits were negatively associated with edge distance, mostly for the traits associated with increasing productivity and the efficient use of resources. Moreover, generally for all three functional types, we observed significantly negative relationships of plasticity in functional traits with edge distance for a greater number of traits compared to the number of traits exhibiting significantly positive associations. These results supported our hypothesis, where we expected increasing plasticity in functional traits with increasing fragmentation. It is generally believed that greater genotypic or phenotypic plasticity helps a species or populations to better adapt to altered habitat conditions. In our study, the smaller sized fragments showed poor soil resources and higher disturbance indexes; therefore, in such conditions, the functional types with greater plasticity in functional traits will adapt better compared to the functional types with lower plasticity. However, the LWHD functional type, being not so efficient in acquiring resources, exhibited greater plasticity in functional traits linked with the conservation of resources compared to the HWMD and HWLD functional types which were able to exploit resources, even in unfavorable conditions, as indicated by their plasticity in functional traits. This was clearly supported by the relative abundance of functional types in smaller fragments compared to the larger fragments. We observed declining species richness and tree cover for all three functional types in smaller fragments, while the decline was greater for the LWHD functional type compared to the HWMD and HWLD functional types. The small fragments in our study region exhibited extremely unfavorable conditions, where the plants need specific strategies for their survival. For the smaller fragments, our observations are supported by Reich (2014), who suggested that under harsher conditions with resource limitations, the strategy of resource conservation and stress tolerance would be more advantageous, whereas under milder conditions with an abundance of resources, the strategy of faster growth with a greater ability to capture resources than competitors would be more advantageous. However, this pattern of resource use is not always common, and there could also be variations in trait co-ordinations in different natural populations (see Niinemets, 2015; Galán Díaz et al., 2021; Solé-Medina et al., 2022).

(c) Role of plasticity in functional traits in determining the structure and productivity of vegetation.

Pistón et al. (2019) suggested that vegetation structure and plant productivity depend on some specific trait combinations, and they argued that all traits are not equally important for determining plant fitness; therefore, it is essential to consider traits of different ecological dimensions for the selection of most important trait combinations. In our study, for explaining the vegetation structure and productivity among the three functional types, step-wise regression results exhibited very different combinations of functional traits. For instance, considering the two main groups of trees with different resource use strategies, i.e., drought-avoiding (LWHD) and drought-tolerant (HWMD and HWLD), we observed that for the drought-avoiding group, the vegetation attributes were mainly explained by the plasticity in functional traits important for nutrient economy, growth, and the conservation of water (i.e., ΔSLA, ΔLPC, and ΔQWsat), whereas for the drought-tolerant group, stem density, species richness, and biomass accumulation capacity were predominantly explained by the plasticity in functional traits important for stability and for acquiring water (i.e., ΔWSG, ΔRWC, and ΔQWsat). The community structure and productivity of tropical trees is influenced by several environmental factors acting together, and their impact varies due to changes in soil water availability. For dry regions, studies have reported that high deciduous species are more susceptible to drought-induced embolism due to lower WSG, and therefore they are mostly killed by drought stress, compared to low deciduous trees (Sobrado, 1997; Markesteijn and Poorter, 2009; Sevanto et al., 2014). Meanwhile, studies in tropical wet regions argue that the less deciduous trees have large sapwood areas and require more water due to greater peak transpiration, which makes their crown conductance more sensitive to vapor pressure deficits (e.g., Siddiq et al., 2017). Surprisingly, the plastic variations in SDWT exhibited very little influence in determining the structure and productivity of vegetation across the forest fragments, although for explaining biomass accumulation capacity in the LWHD and HWMD functional types, SDWT exhibited significant influence. Across the tree species in our forest fragments, 64% of trees showed very small seed mass (< 0.2 g), which indicated the dominance of small size seeds. According to Khurana and Singh (2001), the majority of tropical dry forest species possess orthodox seeds with high dormancy, while small-seeded species exhibit rapid germination, a high rate of seedling growth, and successful colonization in disturbed forest fragments. Further, we found that the variations in the plasticity of functional traits selected by the step-wise regressions were able to explain more than 50% of the variations in stem density and species richness generally for all three functional types. However, for explaining the variations in biomass accumulation capacity, we observed large differences among the functional types, where the variations in the plasticity of functional traits could explain less than 30% of the variations for the LWHD and HWMD functional types and 60% of the variations in biomass accumulation capacity for the HWLD functional type. These results indicate that for modulating productivity of high deciduous species, other environmental factors are playing a major role, compared to the own fitness of the tree species.

Our study showed that the variations in functional trait plasticity and the structural attributes of tree species in three functional types exhibit contrasting affinity with soil water and nutrient contents and disturbances, although the LWHD functional type was comparatively more influenced by soil resources and disturbances compared to the HWMD and HWLD functional types. Moreover, along the declining SMC and edge distance gradients, plasticity in functional traits for the LWHD functional type exhibited greater increase for the traits associated with the conservation of water and resources, whereas for HWMD and HWLD functional types, the traits exhibiting greater plasticity with increasing fragmentation were linked with higher productivity and water transport. The cumulative response of SMC, soil nutrients, and disturbances by plasticity in functional traits was also visible in the relative abundance of functional types in large and small sized fragments. Our analysis furthermore revealed the critical differences in the responses of functional trait plasticity for the coexisting tree species in tropical dry forests, which suggest that the important deciduous endemic species with drought-avoiding strategies might be prone to strategic exclusion under the expected increase in anthropogenic disturbances, habitat fragmentation, and resource limitations.

## Data availability statement

The original contributions presented in the study are included in the article/**Supplementary Material**. Further inquiries can be directed to the corresponding author.

## Author contributions

Conception and design of study: RC, SP, AT, and AR. Acquisition of data: RC, SP, and AT. Analysis and/or interpretation of data: RC and SP. Drafting the manuscript: RC, SP, and AT. Revising the manuscript critically for important intellectual content: RC, SP, AT, LG, AR and JS. All authors contributed to the article and approved the submitted version.

## Glossary

**Table:**

A_max_	maximum saturated photosynthetic rate
CC	canopy cover intensity
CCDBH	ratio of crown cover and diameter at breast height
CDDBH	ratio of crown depth and diameter at breast height
Chl	leaf chlorophyll content
FT	functional type
Gs_max_	maximum saturated stomatal conductance
HCPC	hierarchical clustering on principal components
HWLD	high wood density low deciduous
HWMD	high wood density medium deciduous
LA	leaf size or leaf area
LDMC	leaf dry matter content
LL	leaf lifespan
LNC	leaf nitrogen content
LPC	leaf phosphorus content
LWHD	low wood density high deciduous
QWsat	saturated stem water content
RWC	relative water content
SDWT	seed mass
SLA	specific leaf area
SMC	soil moisture content
TDF	tropical dry forest
HTDBH	ratio of total height and diameter at breast height
WSG	wood specific gravity
WUEi	intrinsic water use efficiency
Ψ_dawn_	leaf water potential at dawn time
Ψ_noon_	leaf water potential at noon time
