# Supplementary material for: Variations in the plasticity of functional traits indicate the differential impacts of abiotic and biotic factors on the structure and growth of trees in tropical dry forest fragments
Source: Front Plant Sci. 2024 Jan 25;14:1181293. doi: 10.3389/fpls.2023.1181293 (PMC10851170; doi:10.3389/fpls.2023.1181293)
Supplement: Supplementary file 1 [file DataSheet_1.docx]

Table S1. Summary of details about the 45 forest fragments (study site), including latitude, longitude, altitude, patch size, patch perimeter, edge distance, species richness, stem density, dominant species, recruitment, mortality and mortality index of tree species. Buchanania sp. (*Buchanania cochinchinensis*), Lagerstroemia sp. (*Lagerstroemia parviflora*), Terminalia sp. (*Terminalia tomentosa*).

| Study site | Latitude | Longitude | Altitude (m) | Patch size (ha) | Patch perimeter (km) | Edge distance (m) | Species richness | Stem density (0.1 ha) | Dominant species based on importance value index (IVI) | Recruitment per yr | Mortality per yr | Mortality Index |
| --- | --- | --- | --- | --- | --- | --- | --- | --- | --- | --- | --- | --- |
| S1 | 24°17'48.05"N | 83° 6'9.86"E | 300.53 | 76.80 | 3.45 | 398 | 21 | 68 | Shorea robusta (20.51) | 2 | 3 | 4.41 |
| S2 | 24°17'26.25"N | 83° 6'34.38"E | 299.62 | 22.50 | 2.28 | 381 | 20 | 71 | Tectona grandis (16.42) | 3 | 2 | 2.82 |
| S3 | 24°17'5.25"N | 83° 6'9.92"E | 299.31 | 24.00 | 2.56 | 357 | 19 | 56 | Shorea robusta (15.73) | 1 | 0 | 0.00 |
| S4 | 24°18'17.84"N | 83° 6'24.12"E | 279.50 | 56.00 | 2.72 | 343 | 14 | 53 | Tectona grandis (26.86) | 0 | 1 | 1.89 |
| S5 | 24°18'21.12"N | 83° 7'44.77"E | 308.46 | 36.19 | 2.79 | 315 | 15 | 45 | Tectona grandis (17.06) | 2 | 1 | 2.22 |
| S6 | 24°17'44.32"N | 83° 7'44.71"E | 309.37 | 29.47 | 2.43 | 308 | 13 | 42 | Tectona grandis (24.79) | 3 | 2 | 4.76 |
| S7 | 24°19'9.56"N | 83° 9'14.17"E | 263.04 | 45.50 | 2.04 | 342 | 16 | 54 | Tectona grandis (20.84) | 2 | 3 | 5.56 |
| S8 | 24°18'28.62"N | 83° 9'23.78"E | 269.44 | 64.60 | 3.24 | 299 | 14 | 47 | Shorea robusta (18.93) | 1 | 0 | 0.00 |
| S9 | 24°17'32.45"N | 83° 8'44.14"E | 284.38 | 92.40 | 3.99 | 337 | 18 | 52 | Shorea robusta (19.85) | 1 | 2 | 3.85 |
| S10 | 24°18'33.23"N | 83°10'58.73"E | 262.74 | 63.00 | 3.06 | 408 | 20 | 65 | Shorea robusta (29.02) | 3 | 1 | 1.54 |
| S11 | 24°17'57.50"N | 83°11'7.47"E | 231.34 | 51.20 | 2.25 | 393 | 19 | 62 | Shorea robusta (23.14) | 2 | 2 | 3.23 |
| S12 | 24°17'33.13"N | 83°10'58.73"E | 246.89 | 25.00 | 2.52 | 345 | 18 | 59 | Shorea robusta (20.20) | 1 | 0 | 0.00 |
| S13 | 24°16'51.60"N | 83°11'30.63"E | 297.79 | 16.00 | 1.56 | 287 | 16 | 50 | Shorea robusta (27.63) | 2 | 0 | 0.00 |
| S14 | 24°16'27.96"N | 83°10'42.82"E | 309.37 | 22.86 | 1.96 | 306 | 17 | 46 | Acacia catechu (23.45) | 0 | 1 | 2.17 |
| S15 | 24°15'33.03"N | 83° 9'48.69"E | 349.61 | 31.58 | 2.62 | 284 | 15 | 39 | Shorea robusta (21.48) | 1 | 0 | 0.00 |
| S16 | 24°16'30.23"N | 83°13'12.78"E | 280.11 | 59.50 | 2.72 | 278 | 15 | 43 | Shorea robusta (19.11) | 3 | 2 | 4.65 |
| S17 | 24°15'56.11"N | 83°14'10.42"E | 302.36 | 63.00 | 2.94 | 256 | 15 | 45 | Shorea robusta (19.89) | 0 | 1 | 2.22 |
| S18 | 24°14'54.12"N | 83°12'57.29"E | 307.54 | 72.20 | 3.78 | 355 | 19 | 56 | Shorea robusta (31.42) | 2 | 3 | 5.36 |
| S19 | 24°24'32.48"N | 83°18'11.54"E | 325.83 | 29.40 | 2.31 | 161 | 8 | 40 | Buchanania sp. (21.70) | 2 | 0 | 0.00 |
| S20 | 24°24'25.08"N | 83°17'53.95"E | 305.41 | 28.00 | 2.04 | 158 | 9 | 37 | Shorea robusta (27.95) | 1 | 0 | 0.00 |
| S21 | 24°24'42.51"N | 83°17'51.48"E | 301.45 | 22.80 | 1.98 | 182 | 9 | 34 | Shorea robusta (23.43) | 0 | 1 | 2.94 |
| S22 | 24°24'29.18"N | 83°17'25.21"E | 286.51 | 18.95 | 1.96 | 186 | 9 | 30 | Buchanania sp. (23.48) | 2 | 0 | 0.00 |
| S23 | 24°24'44.08"N | 83°17'15.63"E | 289.26 | 6.25 | 0.96 | 245 | 10 | 35 | Lagerstroemia sp. (22.58) | 1 | 1 | 2.86 |
| S24 | 24°24'44.42"N | 83°16'53.49"E | 285.6 | 4.57 | 0.79 | 193 | 9 | 28 | Shorea robusta (22.41) | 2 | 0 | 0.00 |
| S25 | 24°24'31.69"N | 83°16'44.92"E | 276.45 | 9.52 | 1.25 | 267 | 11 | 33 | Terminalia sp. (23.75) | 0 | 2 | 6.06 |
| S26 | 24°24'37.27"N | 83°16'29.46"E | 285.6 | 19.20 | 1.35 | 216 | 10 | 31 | Anogeissus latifolia (20.88) | 2 | 1 | 3.23 |
| S27 | 24°24'31.21"N | 83°16'13.36"E | 274.62 | 28.00 | 1.92 | 222 | 10 | 38 | Lagerstroemia sp. (24.33) | 1 | 2 | 5.26 |
| S28 | 24°16'31.14"N | 83°22'48.82"E | 293.83 | 21.00 | 1.44 | 168 | 9 | 36 | Buchanania sp. (20.50) | 2 | 2 | 5.56 |
| S29 | 24°16'38.67"N | 83°22'37.52"E | 290.78 | 21.00 | 1.68 | 164 | 10 | 38 | Lagerstroemia sp. (20.08) | 0 | 1 | 2.63 |
| S30 | 24°16'40.74"N | 83°22'56.43"E | 301.75 | 21.00 | 1.53 | 155 | 8 | 36 | Shorea robusta (33.12) | 1 | 4 | 11.11 |
| S31 | 24°16'54.02"N | 83°22'46.47"E | 287.12 | 15.20 | 1.26 | 183 | 10 | 33 | Buchanania sp. (21.42) | 1 | 0 | 0.00 |
| S32 | 24°16'57.99"N | 83°23'2.20"E | 307.85 | 5.00 | 0.96 | 176 | 10 | 29 | Shorea robusta (21.37) | 1 | 3 | 10.34 |
| S33 | 24°17'8.33"N | 83°23'17.27"E | 300.23 | 8.00 | 1.35 | 167 | 9 | 27 | Shorea robusta (37.18) | 0 | 0 | 0.00 |
| S34 | 24°17'11.51"N | 83°22'50.15"E | 298.7 | 10.53 | 1.31 | 235 | 11 | 39 | Buchanania sp. (20.56) | 2 | 4 | 10.26 |
| S35 | 24°17'36.11"N | 83°22'55.68"E | 308.46 | 12.80 | 1.05 | 186 | 10 | 34 | Shorea robusta (35.05) | 0 | 3 | 8.82 |
| S36 | 24°17'47.11"N | 83°23'9.08"E | 340.77 | 13.33 | 1.52 | 197 | 11 | 30 | Terminalia sp. (28.66) | 1 | 2 | 6.67 |
| S37 | 24°15'42.39"N | 83° 4'15.54"E | 310.90 | 4.57 | 0.79 | 121 | 8 | 19 | Acacia catechu (29.34) | 0 | 0 | 0.00 |
| S38 | 24°16'5.39"N | 83° 5'11.31"E | 306.63 | 3.80 | 0.72 | 103 | 8 | 23 | Terminalia sp. (22.73) | 1 | 3 | 13.04 |
| S39 | 24°16'22.77"N | 83° 3'45.50"E | 273.41 | 3.50 | 0.64 | 96 | 8 | 16 | Lagerstroemia sp. (31.57) | 0 | 2 | 12.50 |
| S40 | 24°16'43.67"N | 83° 4'27.46"E | 299.62 | 1.71 | 0.55 | 68 | 5 | 12 | Acacia catechu (44.90) | 1 | 0 | 0.00 |
| S41 | 24°17'17.28"N | 83° 5'6.99"E | 297.79 | 1.47 | 0.49 | 71 | 6 | 17 | Acacia catechu (24.30) | 0 | 2 | 11.76 |
| S42 | 24°17'27.48"N | 83° 4'9.54"E | 286.21 | 1.50 | 0.46 | 66 | 6 | 16 | Acacia catechu (32.69) | 1 | 3 | 18.75 |
| S43 | 24°17'58.81"N | 83° 5'11.68"E | 298.40 | 1.75 | 0.51 | 79 | 7 | 31 | Lagerstroemia sp. (30.32) | 0 | 2 | 6.45 |
| S44 | 24°18'10.58"N | 83° 4'21.01"E | 280.72 | 1.92 | 0.45 | 92 | 8 | 25 | Lagerstroemia sp. (18.99) | 0 | 3 | 12.00 |
| S45 | 24°18'38.70"N | 83° 4'39.37"E | 292.00 | 12.60 | 1.05 | 83 | 7 | 24 | Acacia catechu (31.37) | 1 | 0 | 0.00 |

Table S2. Average values of edaphic properties (±SE) for the 45 study sites, including soil moisture content (SMC), soil organic carbon (SOC), total nitrogen (N), total phosphorus (P), clay, silt, sand, and bulk density.

| Study site | SMC (%) | SOC (%) | Total N (%) | Total P (%) | Clay (%) | Silt (%) | Sand (%) | Bulk density (g cm^-3^) |
| --- | --- | --- | --- | --- | --- | --- | --- | --- |
| S1 | 15.302±0.548 | 2.135±0.050 | 0.166±0.002 | 0.062±0.001 | 12.167±0.260 | 25.633±0.727 | 62.200±0.651 | 1.443±0.023 |
| S2 | 18.460±0.524 | 2.058±0.018 | 0.177±0.001 | 0.064±0.001 | 12.700±0.608 | 26.033±0.328 | 61.267±0.318 | 1.347±0.020 |
| S3 | 18.968±0.400 | 2.150±0.021 | 0.157±0.003 | 0.065±0.001 | 13.133±0.433 | 24.800±0.321 | 62.067±0.754 | 1.423±0.015 |
| S4 | 13.744±0.474 | 2.643±0.012 | 0.184±0.002 | 0.074±0.001 | 12.167±0.376 | 27.267±0.318 | 60.567±0.694 | 1.327±0.015 |
| S5 | 14.753±0.264 | 2.517±0.010 | 0.179±0.001 | 0.074±0.001 | 11.700±0.529 | 28.667±0.384 | 59.633±0.895 | 1.420±0.012 |
| S6 | 13.779±0.402 | 2.353±0.015 | 0.171±0.000 | 0.082±0.001 | 12.100±0.404 | 26.833±0.318 | 61.067±0.722 | 1.360±0.010 |
| S7 | 16.250±0.439 | 1.882±0.009 | 0.136±0.002 | 0.036±0.002 | 11.700±0.321 | 29.247±0.260 | 59.053±0.566 | 1.330±0.015 |
| S8 | 17.761±0.260 | 2.137±0.015 | 0.147±0.002 | 0.042±0.001 | 11.700±0.416 | 28.567±0.203 | 59.733±0.612 | 1.347±0.020 |
| S9 | 16.658±0.321 | 2.278±0.012 | 0.149±0.001 | 0.042±0.001 | 11.367±0.463 | 27.267±0.318 | 61.367±0.780 | 1.357±0.015 |
| S10 | 15.770±0.221 | 2.042±0.022 | 0.149±0.001 | 0.048±0.001 | 13.500±0.404 | 19.433±0.233 | 67.067±0.536 | 1.383±0.018 |
| S11 | 16.949±0.243 | 1.966±0.017 | 0.145±0.002 | 0.044±0.001 | 12.067±0.406 | 22.167±0.273 | 65.767±0.674 | 1.380±0.017 |
| S12 | 16.930±0.118 | 1.792±0.015 | 0.148±0.001 | 0.050±0.002 | 12.817±0.377 | 20.367±0.296 | 66.817±0.665 | 1.430±0.012 |
| S13 | 14.638±0.402 | 1.868±0.009 | 0.143±0.002 | 0.047±0.001 | 9.600±0.379 | 23.323±0.338 | 67.077±0.232 | 1.370±0.006 |
| S14 | 13.851±0.213 | 2.062±0.021 | 0.144±0.002 | 0.042±0.001 | 8.470±0.430 | 25.027±0.208 | 66.503±0.636 | 1.327±0.012 |
| S15 | 14.611±0.272 | 2.237±0.022 | 0.146±0.002 | 0.049±0.001 | 7.303±0.408 | 26.347±0.260 | 66.350±0.229 | 1.353±0.015 |
| S16 | 15.917±0.178 | 3.049±0.019 | 0.166±0.002 | 0.056±0.001 | 10.447±0.451 | 23.233±0.426 | 66.320±0.869 | 1.383±0.018 |
| S17 | 15.883±0.263 | 2.482±0.014 | 0.168±0.002 | 0.068±0.001 | 11.467±0.500 | 24.317±0.239 | 64.217±0.738 | 1.413±0.018 |
| S18 | 14.667±0.253 | 2.060±0.018 | 0.174±0.002 | 0.083±0.001 | 10.873±0.271 | 22.093±0.241 | 67.033±0.237 | 1.447±0.015 |
| S19 | 10.494±0.136 | 1.565±0.006 | 0.157±0.002 | 0.054±0.001 | 9.103±0.234 | 32.400±0.321 | 58.497±0.546 | 1.300±0.023 |
| S20 | 13.582±0.094 | 1.575±0.007 | 0.146±0.002 | 0.045±0.002 | 8.240±0.315 | 28.783±0.232 | 62.977±0.085 | 1.397±0.015 |
| S21 | 13.907±0.177 | 1.563±0.005 | 0.134±0.002 | 0.035±0.001 | 7.510±0.211 | 31.367±0.203 | 61.123±0.226 | 1.450±0.017 |
| S22 | 8.049±0.045 | 1.483±0.006 | 0.164±0.002 | 0.024±0.001 | 6.193±0.326 | 35.167±0.203 | 58.640±0.433 | 1.347±0.020 |
| S23 | 9.359±0.067 | 1.483±0.006 | 0.158±0.002 | 0.023±0.001 | 4.373±0.329 | 35.267±0.338 | 60.360±0.666 | 1.303±0.015 |
| S24 | 8.604±0.081 | 1.443±0.003 | 0.166±0.002 | 0.022±0.001 | 5.327±0.277 | 33.217±0.232 | 61.457±0.506 | 1.413±0.019 |
| S25 | 11.544±0.179 | 1.609±0.002 | 0.091±0.002 | 0.037±0.002 | 7.373±0.279 | 32.400±0.265 | 60.227±0.543 | 1.347±0.015 |
| S26 | 12.559±0.219 | 1.610±0.002 | 0.091±0.002 | 0.036±0.002 | 8.223±0.277 | 30.317±0.148 | 61.460±0.422 | 1.450±0.017 |
| S27 | 11.551±0.179 | 1.682±0.005 | 0.096±0.002 | 0.026±0.002 | 7.537±0.229 | 29.333±0.328 | 63.130±0.111 | 1.407±0.012 |
| S28 | 10.506±0.186 | 1.528±0.004 | 0.129±0.012 | 0.035±0.002 | 4.533±0.180 | 24.967±0.203 | 70.500±0.023 | 1.297±0.020 |
| S29 | 11.630±0.172 | 1.717±0.004 | 0.107±0.004 | 0.043±0.001 | 6.440±0.276 | 25.100±0.208 | 68.460±0.272 | 1.347±0.015 |
| S30 | 11.650±0.152 | 1.479±0.005 | 0.118±0.008 | 0.024±0.002 | 5.547±0.364 | 27.217±0.232 | 67.237±0.166 | 1.447±0.020 |
| S31 | 9.339±0.122 | 1.692±0.005 | 0.122±0.006 | 0.024±0.001 | 4.657±0.511 | 26.433±0.291 | 68.910±0.221 | 1.400±0.017 |
| S32 | 8.517±0.145 | 1.533±0.004 | 0.133±0.001 | 0.016±0.001 | 3.830±0.223 | 25.817±0.262 | 70.353±0.084 | 1.453±0.009 |
| S33 | 9.436±0.110 | 1.513±0.006 | 0.140±0.002 | 0.033±0.001 | 4.360±0.258 | 26.400±0.265 | 69.240±0.522 | 1.343±0.012 |
| S34 | 10.590±0.175 | 1.406±0.004 | 0.154±0.002 | 0.027±0.001 | 6.140±0.171 | 25.000±0.173 | 68.860±0.336 | 1.303±0.020 |
| S35 | 10.460±0.131 | 1.479±0.010 | 0.145±0.002 | 0.032±0.001 | 4.360±0.314 | 27.933±0.176 | 67.707±0.490 | 1.447±0.015 |
| S36 | 9.297±0.096 | 1.583±0.010 | 0.135±0.002 | 0.023±0.001 | 4.503±0.159 | 26.400±0.265 | 69.097±0.421 | 1.357±0.012 |
| S37 | 8.349±0.106 | 1.353±0.007 | 0.117±0.010 | 0.031±0.001 | 3.540±0.334 | 26.500±0.173 | 69.960±0.458 | 1.403±0.015 |
| S38 | 8.240±0.061 | 1.387±0.005 | 0.126±0.009 | 0.033±0.001 | 3.807±0.167 | 24.217±0.232 | 71.977±0.396 | 1.313±0.019 |
| S39 | 8.582±0.124 | 1.339±0.003 | 0.113±0.006 | 0.022±0.001 | 3.200±0.136 | 27.967±0.203 | 68.833±0.306 | 1.303±0.015 |
| S40 | 4.423±0.091 | 1.368±0.004 | 0.134±0.006 | 0.022±0.001 | 2.373±0.187 | 28.617±0.213 | 69.010±0.378 | 1.247±0.015 |
| S41 | 5.434±0.105 | 1.325±0.006 | 0.146±0.001 | 0.022±0.001 | 1.493±0.144 | 28.033±0.260 | 70.473±0.404 | 1.203±0.026 |
| S42 | 6.487±0.105 | 1.297±0.006 | 0.128±0.008 | 0.032±0.001 | 2.367±0.153 | 30.150±0.236 | 67.483±0.090 | 1.340±0.015 |
| S43 | 9.542±0.115 | 1.468±0.010 | 0.096±0.003 | 0.018±0.001 | 3.813±0.179 | 24.217±0.232 | 71.970±0.407 | 1.340±0.015 |
| S44 | 9.363±0.093 | 1.497±0.004 | 0.11±0.007 | 0.021±0.001 | 4.517±0.176 | 25.033±0.203 | 70.450±0.379 | 1.397±0.015 |
| S45 | 9.618±0.148 | 1.576±0.007 | 0.098±0.003 | 0.019±0.001 | 3.040±0.112 | 26.400±0.265 | 70.560±0.371 | 1.350±0.023 |

Table S3. Average value of soil moisture content (SMC), canopy cover intensity (%), and wood and leaf traits of tree species across the 45 forest fragments. FT, functional type; HWMD, high wood density medium deciduous; HWLD, high wood density low deciduous; LWHD, low wood density high deciduous; WSG, wood specific gravity (g cm^-3^); QWsat, stem water storage capacity (%); CC, canopy cover intensity (%); SLA, specific leaf area (cm^2^ g^-1^); RWC, relative water content (%); LDMC, leaf dry matter content (%); LNC, leaf nitrogen content (% dry weight); LPC, leaf phosphorus content (% dry weight); Gs_max_, maximum saturated stomatal conductance (mol m^-2^ s^-1^); A_max_, maximum saturated photosynthesis (µmol m^-2^ s^-1^); WUEi, intrinsic water use efficiency (µmol mol^-1^); Ψ_dawn_, leaf water potential at dawn (MPa); Ψ_noon_, leaf water potential at noon (MPa); Chl, chlorophyll content (mg g^-1^ fresh weight); LL, leaf life-span (days); LA, leaf area (cm^2^); SDWT, seed mass (g); HTDBH, ratio of total height and diameter at breast height; CDDBH, ratio of crown depth and diameter at breast height; CCDBH, ratio of crown cover and diameter at breast height.

| Species | Family | FT | SMC | WSG | QWsat | CC | SLA | RWC | LDMC | LNC | LPC | Gs_max_ | A_max_ | WUEi | Ψ_dawn_ | Ψ_noon_ | Chl | LL | LA | SDWT | HTDBH | CDDBH | CCDBH |
| --- | --- | --- | --- | --- | --- | --- | --- | --- | --- | --- | --- | --- | --- | --- | --- | --- | --- | --- | --- | --- | --- | --- | --- |
| Acacia catechu | Fabaceae | HWMD | 10.90 | 0.71 | 41.06 | 63.13 | 152.41 | 95.48 | 35.59 | 1.78 | 0.22 | 0.34 | 13.63 | 45.66 | -1.24 | -2.27 | 1.50 | 220.48 | 44.39 | 0.02 | 5.02 | 0.21 | 0.73 |
| Adina cordifolia | Rubiaceae | LWHD | 11.60 | 0.47 | 170.23 | 46.59 | 84.68 | 87.15 | 33.09 | 1.93 | 0.23 | 0.47 | 14.95 | 32.02 | -1.19 | -2.06 | 1.22 | 176.21 | 626.58 | 0.00 | 6.12 | 0.22 | 0.82 |
| Aegle marmelos | Rutaceae | HWMD | 10.50 | 0.71 | 53.45 | 63.33 | 124.45 | 98.25 | 33.76 | 2.08 | 0.28 | 0.45 | 14.46 | 32.33 | -0.99 | -2.38 | 2.15 | 204.17 | 56.31 | 0.17 | 4.45 | 0.26 | 0.83 |
| Albizia odoratissima | Fabaceae | HWMD | 12.96 | 0.67 | 49.84 | 75.28 | 131.71 | 97.03 | 33.52 | 2.20 | 0.24 | 0.40 | 13.94 | 34.49 | -1.57 | -2.72 | 1.25 | 265.00 | 435.57 | 0.05 | 4.60 | 0.38 | 0.82 |
| Anogeissus latifolia | Combretaceae | HWMD | 13.84 | 0.71 | 48.58 | 70.53 | 120.26 | 96.51 | 33.37 | 1.69 | 0.22 | 0.36 | 14.63 | 43.25 | -1.37 | -2.37 | 1.08 | 218.91 | 40.12 | 0.01 | 6.17 | 0.19 | 0.59 |
| Azadirachta indica | Meliaceae | HWLD | 10.80 | 0.63 | 51.50 | 83.03 | 147.71 | 98.15 | 35.14 | 1.77 | 0.24 | 0.41 | 12.30 | 29.82 | -1.25 | -2.16 | 1.27 | 318.17 | 569.28 | 0.16 | 8.35 | 0.12 | 0.44 |
| Bauhinia racemosa | Fabaceae | HWMD | 8.56 | 0.58 | 62.11 | 81.73 | 118.88 | 98.28 | 35.46 | 2.40 | 0.22 | 0.34 | 14.91 | 44.37 | -1.25 | -2.28 | 1.29 | 261.62 | 37.77 | 0.10 | 4.40 | 0.25 | 1.14 |
| Boswellia serrata | Burseraceae | LWHD | 8.15 | 0.44 | 194.15 | 41.98 | 82.95 | 97.49 | 33.54 | 1.77 | 0.26 | 0.23 | 10.50 | 45.56 | -0.91 | -2.90 | 1.03 | 172.88 | 452.46 | 0.09 | 7.57 | 0.14 | 0.56 |
| Bridelia retusa | Phyllanthaceae | HWMD | 12.60 | 0.58 | 43.11 | 77.88 | 110.04 | 88.74 | 34.42 | 1.94 | 0.27 | 0.42 | 14.11 | 33.99 | -1.20 | -1.83 | 1.28 | 242.53 | 133.50 | 0.06 | 7.42 | 0.23 | 0.84 |
| Buchanania cochinchinensis | Anacardiaceae | HWMD | 10.30 | 0.59 | 48.25 | 68.48 | 85.11 | 92.59 | 35.66 | 1.72 | 0.19 | 0.32 | 12.22 | 40.04 | -1.08 | -1.64 | 1.04 | 228.80 | 142.63 | 0.68 | 5.80 | 0.20 | 0.86 |
| Butea monosperma | Fabaceae | LWHD | 6.39 | 0.63 | 54.43 | 53.47 | 65.37 | 76.16 | 38.62 | 1.31 | 0.15 | 0.24 | 10.60 | 43.64 | -1.21 | -2.05 | 0.87 | 210.33 | 857.47 | 1.77 | 4.92 | 0.24 | 0.77 |
| Carissa spinarum | Apocynaceae | HWLD | 8.14 | 0.59 | 45.82 | 82.43 | 119.03 | 96.32 | 32.27 | 1.87 | 0.25 | 0.38 | 14.74 | 38.95 | -1.13 | -2.11 | 1.82 | 259.44 | 7.47 | 0.03 | 3.32 | 0.21 | 0.65 |
| Casearia elliptica | Salicaceae | HWMD | 10.34 | 0.61 | 47.34 | 71.62 | 135.00 | 98.04 | 32.44 | 1.91 | 0.27 | 0.48 | 16.93 | 35.52 | -1.13 | -2.12 | 1.84 | 238.00 | 67.09 | 0.55 | 4.90 | 0.25 | 0.97 |
| Cassia fistula | Fabaceae | HWMD | 9.85 | 0.61 | 53.11 | 73.64 | 136.32 | 96.77 | 33.78 | 2.62 | 0.28 | 0.39 | 15.05 | 38.55 | -0.94 | -2.24 | 1.32 | 220.67 | 77.99 | 0.21 | 5.95 | 0.25 | 0.91 |
| Cassine glauca | Celastraceae | HWLD | 10.85 | 0.67 | 43.28 | 67.70 | 140.99 | 96.11 | 36.61 | 1.66 | 0.24 | 0.34 | 14.22 | 42.26 | -1.10 | -2.39 | 1.11 | 231.92 | 57.28 | 1.11 | 6.33 | 0.20 | 0.71 |
| Ceriscoides turgida | Rubiaceae | LWHD | 8.73 | 0.64 | 43.64 | 60.09 | 84.72 | 86.83 | 32.80 | 1.95 | 0.19 | 0.17 | 5.85 | 34.97 | -1.11 | -2.35 | 0.90 | 224.00 | 25.75 | 0.10 | 4.10 | 0.23 | 1.15 |
| Chloroxylon swietenia | Rutaceae | HWMD | 8.46 | 0.61 | 52.32 | 69.10 | 114.94 | 97.32 | 33.68 | 1.82 | 0.26 | 0.38 | 12.50 | 33.20 | -1.11 | -2.99 | 0.95 | 212.67 | 500.09 | 0.80 | 4.98 | 0.30 | 1.01 |
| Cordia myxa | Boraginaceae | HWMD | 10.52 | 0.62 | 65.89 | 59.30 | 105.19 | 97.35 | 34.59 | 1.73 | 0.24 | 0.44 | 13.81 | 31.27 | -1.09 | -1.97 | 1.72 | 247.50 | 154.90 | 0.17 | 5.73 | 0.24 | 0.96 |
| Dalbergia latifolia | Fabaceae | HWMD | 10.68 | 0.66 | 44.74 | 68.34 | 125.40 | 97.58 | 34.97 | 1.81 | 0.27 | 0.46 | 14.98 | 32.59 | -1.21 | -2.40 | 1.92 | 267.27 | 179.08 | 0.05 | 5.48 | 0.21 | 0.59 |
| Desmodium oojeinense | Fabaceae | LWHD | 8.52 | 0.63 | 46.39 | 59.53 | 75.96 | 79.35 | 36.75 | 1.81 | 0.23 | 0.29 | 11.71 | 40.39 | -1.15 | -1.84 | 1.11 | 217.11 | 722.51 | 0.03 | 4.67 | 0.23 | 0.87 |
| Diospyros melanoxylon | Ebenaceae | HWLD | 10.82 | 0.64 | 44.01 | 78.18 | 85.91 | 91.93 | 35.58 | 1.70 | 0.23 | 0.28 | 11.65 | 45.44 | -1.37 | -2.24 | 1.32 | 238.12 | 63.92 | 1.00 | 4.90 | 0.14 | 0.59 |
| Eugenia jambolana | Myrtaceae | HWLD | 7.75 | 0.65 | 44.18 | 85.85 | 102.40 | 97.27 | 35.67 | 1.85 | 0.25 | 0.38 | 14.83 | 38.87 | -1.15 | -2.40 | 1.80 | 349.67 | 65.56 | 1.57 | 5.28 | 0.31 | 1.05 |
| Flacourtia indica | Salicaceae | HWLD | 11.26 | 0.54 | 49.31 | 65.59 | 109.48 | 97.61 | 35.24 | 1.82 | 0.24 | 0.29 | 13.31 | 47.25 | -1.02 | -2.34 | 1.08 | 226.54 | 43.25 | 0.18 | 3.83 | 0.19 | 1.01 |
| Gardenia latifolia | Rubiaceae | HWLD | 8.24 | 0.66 | 126.51 | 55.84 | 159.19 | 95.22 | 35.98 | 1.86 | 0.24 | 0.34 | 14.68 | 42.74 | -0.96 | -2.00 | 1.24 | 197.72 | 209.34 | 0.01 | 5.61 | 0.26 | 0.85 |
| Grewia serrulata | Tiliaceae | HWMD | 12.08 | 0.63 | 46.59 | 68.24 | 133.51 | 97.13 | 34.51 | 2.32 | 0.26 | 0.37 | 14.88 | 40.31 | -1.20 | -2.05 | 1.44 | 229.17 | 51.21 | 0.17 | 4.05 | 0.19 | 0.79 |
| Hardwickia binata | Fabaceae | HWLD | 9.02 | 0.76 | 43.04 | 84.62 | 118.41 | 95.08 | 36.45 | 1.95 | 0.24 | 0.26 | 11.33 | 46.38 | -0.97 | -1.52 | 1.00 | 340.76 | 13.81 | 0.26 | 7.40 | 0.27 | 0.84 |
| Holarrhena pubescens | Apocynaceae | HWMD | 10.3 | 0.59 | 122.72 | 63.46 | 157.49 | 97.82 | 34.05 | 2.31 | 0.29 | 0.43 | 16.88 | 39.91 | -1.06 | -2.79 | 1.87 | 209.13 | 47.76 | 0.02 | 4.88 | 0.28 | 0.72 |
| Holoptelea integrifolia | Ulmaceae | HWLD | 11.10 | 0.65 | 96.63 | 71.07 | 133.59 | 95.67 | 34.67 | 2.10 | 0.25 | 0.35 | 12.99 | 38.06 | -1.07 | -2.24 | 1.34 | 219.75 | 55.11 | 0.03 | 4.88 | 0.24 | 0.62 |
| Lagerstroemia parviflora | Lythraceae | HWLD | 10.60 | 0.63 | 88.48 | 61.26 | 162.38 | 81.07 | 34.49 | 1.95 | 0.25 | 0.30 | 14.12 | 48.79 | -1.14 | -2.52 | 1.37 | 200.30 | 112.11 | 0.03 | 5.47 | 0.18 | 0.61 |
| Lannea coromandelica | Anacardiaceae | LWHD | 10.85 | 0.62 | 201.37 | 55.69 | 143.67 | 94.98 | 32.83 | 2.29 | 0.23 | 0.36 | 15.42 | 43.35 | -0.86 | -2.76 | 1.19 | 177.51 | 180.74 | 0.12 | 6.25 | 0.19 | 0.88 |
| Madhuca latifolia | Sapotaceae | LWHD | 8.63 | 0.61 | 76.41 | 69.19 | 91.93 | 90.19 | 34.80 | 1.74 | 0.22 | 0.24 | 12.82 | 52.99 | -1.19 | -1.96 | 1.05 | 231.70 | 234.77 | 2.19 | 5.67 | 0.18 | 0.88 |
| Mitragyna parvifolia | Rubiaceae | HWMD | 10.64 | 0.59 | 66.20 | 69.60 | 162.96 | 95.95 | 35.43 | 2.04 | 0.25 | 0.35 | 14.86 | 42.51 | -1.00 | -2.10 | 1.47 | 220.33 | 73.21 | 0.00 | 5.82 | 0.27 | 1.30 |
| Nyctanthes arbor-tristis | Oleaceae | LWHD | 6.57 | 0.59 | 157.16 | 54.07 | 127.29 | 92.62 | 36.90 | 1.44 | 0.14 | 0.17 | 10.89 | 65.34 | -1.37 | -2.40 | 0.82 | 206.67 | 54.70 | 0.06 | 2.70 | 0.11 | 0.64 |
| Phyllanthus emblica | Phyllanthaceae | HWLD | 9.72 | 0.64 | 119.12 | 59.78 | 120.99 | 96.31 | 34.20 | 2.02 | 0.23 | 0.28 | 12.88 | 47.47 | -1.26 | -2.71 | 0.85 | 216.70 | 220.67 | 0.03 | 8.96 | 0.15 | 0.50 |
| Pterocarpus marsupium | Fabaceae | HWMD | 10.70 | 0.74 | 48.78 | 65.01 | 152.13 | 97.90 | 33.74 | 2.33 | 0.28 | 0.37 | 15.21 | 40.99 | -1.05 | -2.23 | 1.35 | 228.80 | 44.17 | 0.49 | 8.01 | 0.22 | 0.69 |
| Schleichera oleosa | Sapindaceae | HWMD | 12.85 | 0.62 | 52.50 | 63.10 | 139.17 | 98.30 | 34.70 | 2.40 | 0.30 | 0.41 | 15.04 | 36.76 | -1.13 | -2.25 | 1.63 | 233.50 | 82.33 | 0.63 | 4.40 | 0.34 | 0.99 |
| Schrebera swietenioides | Oleaceae | HWMD | 11.20 | 0.61 | 49.87 | 69.17 | 152.07 | 98.37 | 34.61 | 2.13 | 0.27 | 0.38 | 13.51 | 35.89 | -1.15 | -2.27 | 1.40 | 234.67 | 616.30 | 0.12 | 5.28 | 0.23 | 0.86 |
| Semecarpus anacardium | Anacardiaceae | LWHD | 11.65 | 0.59 | 159.72 | 51.14 | 87.52 | 94.30 | 33.40 | 1.81 | 0.25 | 0.36 | 15.68 | 43.76 | -0.93 | -1.94 | 1.75 | 188.38 | 239.07 | 1.06 | 5.72 | 0.31 | 1.10 |
| Senna siamea | Fabaceae | HWLD | 9.38 | 0.62 | 47.27 | 81.20 | 145.52 | 96.15 | 34.82 | 1.75 | 0.21 | 0.26 | 11.29 | 43.28 | -1.44 | -2.24 | 1.07 | 333.08 | 433.47 | 0.02 | 5.17 | 0.13 | 0.52 |
| Shorea robusta | Dipterocarpaceae | HWLD | 14.67 | 0.77 | 38.95 | 87.43 | 135.71 | 98.16 | 34.77 | 2.12 | 0.22 | 0.31 | 13.82 | 44.47 | -1.19 | -1.60 | 1.28 | 347.50 | 136.03 | 1.27 | 6.76 | 0.16 | 0.55 |
| Soymida febrifuga | Meliaceae | HWLD | 11.56 | 0.64 | 73.47 | 69.56 | 117.42 | 96.92 | 34.12 | 1.95 | 0.24 | 0.31 | 13.43 | 46.47 | -1.09 | -1.73 | 1.13 | 223.47 | 50.50 | 0.10 | 5.32 | 0.18 | 0.72 |
| Tectona grandis | Lamiaceae | HWMD | 12.83 | 0.72 | 67.81 | 57.12 | 136.17 | 95.30 | 35.80 | 1.78 | 0.26 | 0.41 | 15.64 | 38.38 | -1.15 | -2.13 | 1.65 | 196.44 | 980.73 | 0.09 | 7.05 | 0.26 | 0.88 |
| Terminalia chebula | Combretaceae | HWMD | 12.46 | 0.64 | 72.66 | 58.61 | 158.25 | 97.76 | 33.22 | 2.39 | 0.25 | 0.40 | 15.93 | 39.80 | -0.77 | -1.41 | 1.91 | 196.33 | 209.13 | 1.06 | 8.80 | 0.29 | 1.00 |
| Terminalia tomentosa | Combretaceae | HWMD | 12.59 | 0.74 | 63.61 | 51.22 | 149.96 | 96.16 | 33.70 | 2.17 | 0.27 | 0.32 | 15.13 | 51.13 | -0.96 | -1.90 | 1.70 | 180.90 | 183.02 | 2.23 | 6.70 | 0.18 | 0.70 |
| Uvaria tomentosa | Annonaceae | HWLD | 10.48 | 0.63 | 54.79 | 67.11 | 95.24 | 97.64 | 34.15 | 2.03 | 0.24 | 0.32 | 13.93 | 43.08 | -1.05 | -2.17 | 1.27 | 228.64 | 46.84 | 0.74 | 6.89 | 0.24 | 0.76 |
| Ziziphus glaberrima | Rhamnaceae | HWMD | 9.53 | 0.56 | 54.83 | 66.45 | 149.50 | 98.64 | 33.10 | 2.28 | 0.31 | 0.42 | 16.16 | 38.69 | -1.12 | -2.21 | 1.58 | 241.58 | 8.10 | 1.85 | 4.56 | 0.28 | 1.02 |
| Ziziphus nummularia | Rhamnaceae | HWLD | 9.81 | 0.58 | 52.72 | 67.25 | 148.87 | 97.45 | 34.87 | 1.56 | 0.25 | 0.32 | 13.34 | 41.81 | -0.97 | -2.00 | 1.16 | 231.00 | 8.60 | 1.48 | 5.15 | 0.18 | 0.83 |
| Average |  |  | 10.43 | 0.63 | 71.87 | 66.68 | 124.21 | 94.75 | 34.59 | 1.95 | 0.24 | 0.35 | 13.74 | 41.21 | -1.12 | -2.20 | 1.35 | 233.32 | 205.38 | 0.49 | 5.65 | 0.22 | 0.81 |

Table S4. Summary of Pearson’s correlations among the functional traits, including WSG, wood specific gravity (g cm^-3^); QWsat, stem water storage capacity (%); CC, canopy cover intensity (%); SLA, specific leaf area (cm^2^ g^-1^); RWC, relative water content (%); LDMC, leaf dry matter content (%); LNC, leaf nitrogen content (% dry weight); LPC, leaf phosphorus content (% dry weight); Gs_max_, maximum saturated stomatal conductance (mol m^-2^ s^-1^); A_max_, maximum saturated photosynthesis (µmol m^-2^ s^-1^); WUEi, intrinsic water use efficiency (µmol mol^-1^); Ψ_dawn_, leaf water potential at dawn (MPa); Ψ_noon_, leaf water potential at noon (MPa); Chl, chlorophyll content (mg g^-1^ fresh weight); LL, leaf life-span (days); LA, leaf area (cm^2^); SDWT, seed mass (g); HTDBH, ratio of total height and diameter at breast height; CDDBH, ratio of crown depth and diameter at breast height; CCDBH, ratio of crown cover and diameter at breast height. nsP > 0.05, *P < 0.05, **P < 0.01, ***P < 0.001. N = 47.

|  | WSG | QWsat | CC | SLA | RWC | LDMC | LNC | LPC | Gs_max_ | A_max_ | WUEi | Ψ_dawn_ | Ψ_noon_ | Chl | LL | LA | SDWT | HTDBH | CDDBH |
| --- | --- | --- | --- | --- | --- | --- | --- | --- | --- | --- | --- | --- | --- | --- | --- | --- | --- | --- | --- |
| QWsat | **-0.48***** |  |  |  |  |  |  |  |  |  |  |  |  |  |  |  |  |  |  |
| CC | **0.33*** | **-0.65***** |  |  |  |  |  |  |  |  |  |  |  |  |  |  |  |  |  |
| SLA | **0.32*** | -0.08ns | 0.15ns |  |  |  |  |  |  |  |  |  |  |  |  |  |  |  |  |
| RWC | 0.12ns | -0.07ns | **0.30*** | **0.48***** |  |  |  |  |  |  |  |  |  |  |  |  |  |  |  |
| LDMC | 0.18ns | -0.19ns | 0.07ns | -0.16ns | **-0.38**** |  |  |  |  |  |  |  |  |  |  |  |  |  |  |
| LNC | 0.08ns | -0.01ns | 0.13ns | **0.44**** | **0.40**** | **-0.49***** |  |  |  |  |  |  |  |  |  |  |  |  |  |
| LPC | -0.01ns | -0.11ns | 0.07ns | **0.43**** | **0.50***** | **-0.50***** | **0.59***** |  |  |  |  |  |  |  |  |  |  |  |  |
| Gs_max_ | 0.01ns | -0.12ns | 0.11ns | **0.33*** | **0.39**** | **-0.39**** | **0.39**** | **0.66***** |  |  |  |  |  |  |  |  |  |  |  |
| A_max_ | 0.06ns | 0.06ns | 0.03ns | **0.50***** | **0.43**** | **-0.31*** | **0.48***** | **0.63***** | **0.78***** |  |  |  |  |  |  |  |  |  |  |
| WUEi | 0.06ns | **0.29*** | -0.18ns | 0.01ns | -0.15ns | **0.33*** | -0.23ns | **-0.45**** | **-0.72***** | -0.18ns |  |  |  |  |  |  |  |  |  |
| Ψ_dawn_ | -0.09ns | **0.28*** | **-0.36*** | 0.13ns | 0.15ns | -0.21ns | **0.29*** | **0.38**** | 0.11ns | 0.24ns | -0.04ns |  |  |  |  |  |  |  |  |
| Ψ_noon_ | 0.25ns | **-0.32*** | 0.18ns | -0.08ns | -0.14ns | 0.21ns | -0.04ns | -0.12ns | -0.01ns | 0.05ns | 0.05ns | 0.22ns |  |  |  |  |  |  |  |
| Chl | 0.15ns | -0.13ns | 0.04ns | **0.33*** | **0.37**** | **-0.34*** | **0.37*** | **0.62***** | **0.72***** | **0.68***** | **-0.40**** | **0.28*** | 0.10ns |  |  |  |  |  |  |
| LL | **0.33*** | **-0.54***** | **0.84***** | 0.05ns | 0.24ns | 0.21ns | -0.04ns | -0.06ns | 0.00ns | -0.13ns | -0.15ns | **-0.38**** | 0.22ns | 0.00ns |  |  |  |  |  |
| LA | -0.12ns | 0.14ns | **-0.30*** | -0.25ns | **-0.40**** | 0.26ns | **-0.29*** | -0.19ns | 0.02ns | -0.17ns | -0.22ns | -0.18ns | -0.05ns | -0.18ns | -0.13ns |  |  |  |  |
| SDWT | 0.12ns | -0.19ns | 0.00ns | -0.13ns | -0.05ns | 0.10ns | -0.12ns | 0.03ns | -0.12ns | 0.08ns | 0.22ns | 0.17ns | **0.27*** | 0.06ns | 0.07ns | -0.03ns |  |  |  |
| HTDBH | 0.20ns | 0.12ns | -0.06ns | 0.12ns | 0.13ns | -0.09ns | 0.13ns | 0.17ns | 0.11ns | 0.12ns | -0.09ns | **0.29*** | 0.20ns | -0.05ns | 0.03ns | 0.20ns | 0.07ns |  |  |
| CDDBH | 0.08ns | -0.15ns | 0.03ns | 0.05ns | 0.10ns | -0.15ns | **0.40**** | **0.42**** | **0.49***** | **0.39**** | **-0.46**** | 0.18ns | -0.01ns | **0.42**** | -0.02ns | 0.03ns | 0.05ns | -0.13ns |  |
| CCDBH | -0.21ns | -0.08ns | -0.08ns | -0.09ns | 0.01ns | -0.08ns | 0.24ns | 0.16ns | 0.15ns | 0.15ns | -0.20ns | **0.36*** | 0.13ns | 0.19ns | -0.15ns | -0.10ns | 0.14ns | -0.26ns | **0.65***** |

Table S5. Description of the three clusters of 47 tropical dry forest tree species by quantitative variables. WUEi, intrinsic water use efficiency; LDMC, leaf dry matter content; Gs_max_, maximum saturated stomatal conductance; A_max_, maximum saturated photosynthetic rate; Chl, chlorophyll content; LPC, leaf phosphorus content; LNC, leaf nitrogen content; RWC, relative water content; QWsat, stem water storage capacity. nsP > 0.05, *P < 0.05, **P < 0.01, ***P < 0.001.

| Cluster 1 | V-test | Mean in category | Overall mean | SD in category | Overall SD | P value |
| --- | --- | --- | --- | --- | --- | --- |
| QWsat | 3.682 | 156.0 | 82.11 | 53.00 | 49.36 | 0.0002*** |
|  |  |  |  |  |  |  |
| Cluster 2 | V-test | Mean in category | Overall mean | SD in category | Overall SD | P value |
| Gs_max_ | 4.930 | 0.388 | 0.345 | 0.041 | 0.066 | 0.0000*** |
| A_max_ | 4.325 | 14.67 | 13.79 | 1.037 | 1.544 | 0.0000*** |
| Chl | 4.106 | 1.505 | 1.337 | 0.280 | 0.311 | 0.0000*** |
| LPC | 3.683 | 0.258 | 0.243 | 0.022 | 0.032 | 0.0002*** |
| LNC | 3.597 | 2.066 | 1.939 | 0.243 | 0.269 | 0.0003*** |
| RWC | 2.301 | 96.26 | 94.74 | 2.731 | 5.059 | 0.0214* |
|  |  |  |  |  |  |  |
| Cluster 3 | V-test | Mean in category | Overall mean | SD in category | Overall SD | P value |
| WUEi | 3.808 | 38.460 | 41.674 | 4.953 | 6.446 | 0.0001*** |
| LDMC | 2.751 | 34.299 | 34.741 | 0.958 | 1.226 | 0.0059** |

Table S6. Summary of step-wise regressions relating community weighted means of plasticity in functional traits with soil physicochemical properties and disturbances, across 45 forest fragments. LWHD, low wood density high deciduous functional type; HWMD, high wood density medium deciduous functional type; HWLD, high wood density low deciduous functional type; TDF, total species combined; WSG, wood specific gravity (g cm^-3^); QWsat, stem water storage capacity (%); CC, canopy cover intensity (%); SLA, specific leaf area (cm^2^ g^-1^); RWC, relative water content (%); LDMC, leaf dry matter content (%); LNC, leaf nitrogen content (% dry weight); LPC, leaf phosphorus content (% dry weight); Gs_max_, maximum saturated stomatal conductance (mol m^-2^ s^-1^); A_max_, maximum saturated photosynthesis (µmol m^-2^ s^-1^); WUEi, intrinsic water use efficiency (µmol mol^-1^); Ψ_dawn_, leaf water potential at dawn (MPa); Ψ_noon_, leaf water potential at noon (MPa); Chl, chlorophyll content (mg g^-1^ fresh weight); LL, leaf life-span (days); LA, leaf area (cm^2^); SDWT, seed mass (g); HTDBH, ratio of total height and diameter at breast height; CDDBH, ratio of crown depth and diameter at breast height; CCDBH, ratio of crown cover and diameter at breast height. The “Δ” sign represents plasticity. nsP > 0.05, *P < 0.05, **P < 0.01, ***P < 0.001.

| **Functional type** | **Functional trait** | **ANOVA** | **Variable** | **Estimate** | **Std. Error** | ***t*-value** | ***P*-value** |
| --- | --- | --- | --- | --- | --- | --- | --- |
| LWHD | ΔWSG | RSE = 4.987, df = 32, *R^2^* = 0.403, Adj. *R^2^* = 0.291, *F* = 3.603, *P* = 0.008**, AIC = 131.61 | Intercept | -45.343 | 24.165 | -1.876 | 0.070ns |
|  |  |  | Organic C | 6.507 | 2.082 | 3.125 | 0.004** |
|  |  |  | Total P | -204.642 | 83.570 | -2.449 | 0.020* |
|  |  |  | Clay | 1.174 | 0.508 | 2.311 | 0.027* |
|  |  |  | Bulk density | 37.064 | 16.637 | 2.228 | 0.033* |
|  |  |  | Patch size | 0.217 | 0.104 | 2.093 | 0.044* |
|  |  |  | Patch perimeter | -6.208 | 3.043 | -2.040 | 0.050ns |
|  | ΔQWsat | RSE = 4.664, df = 34, *R^2^* = 0.362, Adj. *R^2^* = 0.287, *F* = 4.827, *P* = 0.003**, AIC = 172.57 | Intercept | -50.295 | 21.480 | -2.341 | 0.025* |
|  |  |  | Organic C | 5.286 | 1.712 | 3.088 | 0.004** |
|  |  |  | Total P | -173.364 | 73.906 | -2.346 | 0.025* |
|  |  |  | Bulk density | 40.786 | 14.902 | 2.737 | 0.010* |
|  |  |  | Patch size | 0.093 | 0.045 | 2.068 | 0.046* |
|  | ΔCC | RSE = 5.024, df = 34, *R^2^* = 0.303, Adj. *R^2^* = 0.221, *F* = 3.692, *P* = 0.013*, AIC = 130.55 | Intercept | -42.361 | 23.137 | -1.831 | 0.076ns |
|  |  |  | Organic C | 4.109 | 1.844 | 2.229 | 0.033* |
|  |  |  | Total P | -180.508 | 79.608 | -2.267 | 0.030* |
|  |  |  | Bulk density | 38.138 | 16.051 | 2.376 | 0.023* |
|  |  |  | Patch size | 0.103 | 0.049 | 2.109 | 0.042* |
|  | ΔSLA | RSE = 4.032, df = 33, *R^2^* = 0.449, Adj. *R^2^* = 0.346, *F* = 4.348, *P* = 0.003**, AIC = 115.04 | Intercept | -62.516 | 21.424 | -2.918 | 0.006** |
|  |  |  | Organic C | 6.777 | 1.960 | 3.458 | 0.002** |
|  |  |  | Total P | -176.440 | 66.012 | -2.673 | 0.012* |
|  |  |  | Bulk density | 45.375 | 13.899 | 3.265 | 0.003** |
|  |  |  | Patch size | 0.192 | 0.087 | 2.207 | 0.035* |
|  |  |  | Patch perimeter | -4.111 | 2.622 | -1.568 | 0.127ns |
|  | ΔRWC | RSE = 3.891, df = 34, *R^2^* = 0.465, Adj. *R^2^* = 0.402, *F* = 7.393, *P* = 0.000***, AIC = 110.63 | Intercept | -44.359 | 17.922 | -2.475 | 0.018* |
|  |  |  | Organic C | 5.884 | 1.428 | 4.120 | 0.000*** |
|  |  |  | Total P | -194.058 | 61.663 | -3.147 | 0.003** |
|  |  |  | Bulk density | 35.279 | 12.433 | 2.838 | 0.008** |
|  |  |  | Patch size | 0.102 | 0.038 | 2.715 | 0.010* |
|  | ΔLDMC | RSE = 5.165, df = 32, *R^2^* = 0.462, Adj. *R^2^* = 0.341, *F* = 3.803, *P* = 0.004**, AIC = 135.11 | Intercept | -67.378 | 27.469 | -2.453 | 0.020* |
|  |  |  | Organic C | 6.676 | 2.515 | 2.655 | 0.012* |
|  |  |  | Total P | -237.022 | 87.039 | -2.723 | 0.011* |
|  |  |  | Bulk density | 54.769 | 17.831 | 3.072 | 0.004** |
|  |  |  | Patch size | 0.303 | 0.114 | 2.667 | 0.012* |
|  |  |  | Patch perimeter | -7.249 | 3.533 | -2.052 | 0.049* |
|  |  |  | Mortality index | -0.355 | 0.251 | -1.412 | 0.168ns |
|  | ΔLNC | RSE = 12.500, df = 33, *R^2^* = 0.445, Adj. *R^2^* = 0.340, *F* = 4.269, *P* = 0.003**, AIC = 203.30 | Intercept | -181.201 | 66.418 | -2.728 | 0.010* |
|  |  |  | Organic C | 20.450 | 6.077 | 3.365 | 0.002** |
|  |  |  | Total P | -581.646 | 204.648 | -2.842 | 0.008** |
|  |  |  | Bulk density | 134.745 | 43.089 | 3.127 | 0.004** |
|  |  |  | Patch size | 0.635 | 0.270 | 2.353 | 0.025* |
|  |  |  | Patch perimeter | -12.659 | 8.129 | -1.557 | 0.129ns |
|  | ΔLPC | RSE = 15.900, df = 34, *R^2^* = 0.329, Adj. *R^2^* = 0.250, *F* = 4.160, *P* = 0.008**, AIC = 220.40 | Intercept | -144.589 | 73.213 | -1.975 | 0.056ns |
|  |  |  | Organic C | 16.753 | 5.835 | 2.871 | 0.007** |
|  |  |  | Total P | -602.049 | 251.901 | -2.390 | 0.023* |
|  |  |  | Bulk density | 118.922 | 50.791 | 2.341 | 0.025* |
|  |  |  | Patch size | 0.295 | 0.154 | 1.919 | 0.063ns |
|  | ΔGs_max_ | RSE = 15.510, df = 34, *R^2^* = 0.357, Adj. *R^2^* = 0.282, *F* = 4.727, *P* = 0.004**, AIC = 218.51 | Intercept | -163.491 | 71.452 | -2.288 | 0.028* |
|  |  |  | Organic C | 18.193 | 5.694 | 3.195 | 0.003** |
|  |  |  | Total P | -552.514 | 245.841 | -2.247 | 0.031* |
|  |  |  | Bulk density | 128.269 | 49.569 | 2.588 | 0.014* |
|  |  |  | Patch size | 0.337 | 0.150 | 2.246 | 0.031* |
|  | ΔA_max_ | RSE = 13.590, df = 33, *R^2^* = 0.421, Adj. *R^2^* = 0.312, *F* = 3.877, *P* = 0.005**, AIC = 209.83 | Intercept | -186.691 | 72.220 | -2.585 | 0.015* |
|  |  |  | Organic C | 25.667 | 6.607 | 3.885 | 0.000*** |
|  |  |  | Total P | -543.267 | 222.524 | -2.441 | 0.020* |
|  |  |  | Bulk density | 127.811 | 46.852 | 2.728 | 0.010* |
|  |  |  | Patch size | 0.601 | 0.294 | 2.048 | 0.049* |
|  |  |  | Patch perimeter | -14.947 | 8.839 | -1.691 | 0.101* |
|  | ΔWUEi | RSE = 25.200, df = 33, *R^2^* = 0.385, Adj. *R^2^* = 0.292, *F* = 4.14, *P* = 0.005**, AIC = 257.18 | Intercept | -196.414 | 115.758 | -1.697 | 0.099ns |
|  |  |  | Organic C | 34.457 | 10.129 | 3.402 | 0.002** |
|  |  |  | Total P | -544.808 | 328.001 | -1.661 | 0.106ns |
|  |  |  | Clay | 2.347 | 1.586 | 1.480 | 0.148ns |
|  |  |  | Bulk density | 143.403 | 79.721 | 1.799 | 0.081ns |
|  |  |  | Mortality index | -1.658 | 1.138 | -1.456 | 0.155ns |
|  | ΔΨ_dawn_ | RSE = 21.360, df = 35, *R^2^* = 0.216, Adj. *R^2^* = 0.149, *F* = 3.21, *P* = 0.035*, AIC = 242.58 | Intercept | 204.487 | 96.711 | 2.114 | 0.042* |
|  |  |  | Organic C | -17.291 | 8.497 | -2.035 | 0.049* |
|  |  |  | Clay | -2.034 | 1.143 | -1.780 | 0.084ns |
|  |  |  | Bulk density | -151.431 | 67.427 | -2.246 | 0.031* |
|  | ΔΨ_noon_ | RSE = 18.520, df = 31, *R^2^* = 0.451, Adj. *R^2^* = 0.305, *F* = 3.086, *P* = 0.012*, AIC = 235.45 | Intercept | 213.556 | 99.868 | 2.138 | 0.041* |
|  |  |  | Organic C | -26.031 | 9.345 | -2.786 | 0.009** |
|  |  |  | Total P | 942.587 | 312.293 | 3.018 | 0.005** |
|  |  |  | Clay | -3.925 | 2.498 | -1.571 | 0.127ns |
|  |  |  | Bulk density | -149.671 | 64.243 | -2.330 | 0.027* |
|  |  |  | Patch size | -0.876 | 0.404 | -2.167 | 0.038* |
|  |  |  | Patch perimeter | 20.372 | 12.841 | 1.587 | 0.123ns |
|  |  |  | Edge distance | 0.255 | 0.088 | 2.895 | 0.007** |
|  | ΔChl | RSE = 14.960, df = 32, *R^2^* = 0.476, Adj. *R^2^* = 0.358, *F* = 4.03, *P* = 0.003**, AIC = 218.09 | Intercept | -254.970 | 79.581 | -3.204 | 0.003** |
|  |  |  | Organic C | 27.308 | 7.286 | 3.748 | 0.000*** |
|  |  |  | Total P | -481.994 | 252.166 | -1.911 | 0.065ns |
|  |  |  | Bulk density | 188.627 | 51.660 | 3.651 | 0.000*** |
|  |  |  | Patch size | 0.763 | 0.329 | 2.320 | 0.027* |
|  |  |  | Patch perimeter | -21.303 | 10.235 | -2.081 | 0.046* |
|  |  |  | Mortality index | -0.935 | 0.728 | -1.285 | 0.208ns |
|  | ΔLL | RSE = 4.659, df = 33, *R^2^* = 0.425, Adj. *R^2^* = 0.318, *F* = 3.948, *P* = 0.005**, AIC = 126.30 | Intercept | -51.492 | 24.752 | -2.080 | 0.046* |
|  |  |  | Organic C | 6.780 | 2.265 | 2.994 | 0.005** |
|  |  |  | Total P | -231.450 | 76.266 | -3.035 | 0.005** |
|  |  |  | Bulk density | 37.720 | 16.058 | 2.349 | 0.025* |
|  |  |  | Patch size | 0.280 | 0.101 | 2.786 | 0.009** |
|  |  |  | Patch perimeter | -5.818 | 3.029 | -1.920 | 0.064ns |
|  | ΔLA | RSE = 12.532, df = 34, *R^2^* = 0.241, Adj. *R^2^* = 0.151, *F* = 2.691, *P* = 0.047*, AIC = 201.85 | Intercept | -77.604 | 57.714 | -1.345 | 0.188ns |
|  |  |  | Organic C | 9.385 | 4.599 | 2.040 | 0.049* |
|  |  |  | Total P | -402.911 | 198.575 | -2.029 | 0.050ns |
|  |  |  | Bulk density | 72.472 | 40.039 | 1.810 | 0.079ns |
|  |  |  | Patch size | 0.240 | 0.121 | 1.980 | 0.056ns |
|  | ΔSDWT | RSE = 10.153, df = 32, *R^2^* = 0.472, Adj. *R^2^* = 0.353, *F* = 3.927, *P* = 0.004**, AIC = 187.79 | Intercept | -138.894 | 53.966 | -2.574 | 0.015* |
|  |  |  | Organic C | 14.233 | 4.941 | 2.881 | 0.007** |
|  |  |  | Total P | -399.274 | 171.000 | -2.335 | 0.026* |
|  |  |  | Bulk density | 109.900 | 35.032 | 3.137 | 0.004** |
|  |  |  | Patch size | 0.661 | 0.223 | 2.960 | 0.006** |
|  |  |  | Patch perimeter | -19.564 | 6.941 | -2.819 | 0.008** |
|  |  |  | Mortality index | -0.940 | 0.494 | -1.904 | 0.066ns |
|  | ΔHTDBH | RSE = 19.071, df = 34, *R^2^* = 0.356, Adj. *R^2^* = 0.282, *F* = 4.695, *P* = 0.004**, AIC = 234.59 | Intercept | -174.329 | 87.821 | -1.985 | 0.055ns |
|  |  |  | Organic C | 19.391 | 6.999 | 2.771 | 0.009** |
|  |  |  | Total P | -835.091 | 302.161 | -2.764 | 0.009** |
|  |  |  | Bulk density | 146.310 | 60.925 | 2.401 | 0.022* |
|  |  |  | Patch size | 0.432 | 0.185 | 2.340 | 0.025* |
|  | ΔCDDBH | RSE = 15.973, df = 34, *R^2^* = 0.396, Adj. *R^2^* = 0.325, *F* = 5.565, *P* = 0.001**, AIC = 220.76 | Intercept | -169.952 | 73.545 | -2.311 | 0.027* |
|  |  |  | Organic C | 14.198 | 5.861 | 2.422 | 0.021* |
|  |  |  | Total P | -766.002 | 253.045 | -3.027 | 0.005** |
|  |  |  | Bulk density | 150.922 | 51.022 | 2.958 | 0.006** |
|  |  |  | Patch size | 0.344 | 0.155 | 2.227 | 0.033* |
|  | ΔCCDBH | RSE = 17.742, df = 32, *R^2^* = 0.403, Adj. *R^2^* = 0.269, *F* = 2.994, *P* = 0.016*, AIC = 231.37 | Intercept | -303.873 | 94.636 | -3.211 | 0.003** |
|  |  |  | Organic C | 25.391 | 8.675 | 2.927 | 0.006** |
|  |  |  | Total P | -611.785 | 295.524 | -2.070 | 0.047* |
|  |  |  | Bulk density | 222.713 | 61.224 | 3.638 | 0.000*** |
|  |  |  | Patch size | 0.783 | 0.387 | 2.023 | 0.052ns |
|  |  |  | Patch perimeter | -20.701 | 11.551 | -1.792 | 0.083ns |
|  |  |  | Edge distance | -0.092 | 0.070 | -1.316 | 0.198ns |
| HWMD | ΔWSG | RSE = 7.105, df = 42, *R^2^* = 0.180, Adj. *R^2^* = 0.120, *F* = 2.99, *P* = 0.042*, AIC = 180.28 | Intercept | 2.586 | 8.320 | 0.311 | 0.758ns |
|  |  |  | Organic C | 5.576 | 2.357 | 2.366 | 0.023* |
|  |  |  | Patch size | -0.100 | 0.069 | -1.446 | 0.156ns |
|  | ΔQWsat | RSE = 6.521, df = 42, *R^2^* = 0.168, Adj. *R^2^* = 0.117, *F* = 2.76, *P* = 0.044*, AIC = 172.57 | Intercept | 15.984 | 7.555 | 2.116 | 0.041* |
|  |  |  | Organic C | 4.001 | 2.173 | 1.9841 | 0.047* |
|  |  |  | Edge distance | -0.035 | 0.021 | -1.647 | 0.107ns |
|  | ΔCC | RSE = 6.813, df = 43, *R^2^* = 0.178, Adj. *R^2^* = 0.114, *F* = 1.785, *P* = 0.048*, AIC = 175.59 | Intercept | 23.951 | 4.793 | 4.997 | 0.000*** |
|  |  |  | Edge distance | -0.041 | 0.022 | -1.987 | 0.048* |
|  | ΔSLA | RSE = 5.963, df = 43, *R^2^* = 0.180, Adj. *R^2^* = 0.113, *F* = 2.258, *P* = 0.040*, AIC = 162.66 | Intercept | 19.272 | 3.182 | 6.057 | 0.000*** |
|  |  |  | Organic C | 2.512 | 1.671 | 1.950 | 0.048* |
|  | ΔRWC | RSE = 5.715, df = 42, *R^2^* = 0.231, Adj. *R^2^* = 0.195, *F* = 6.312, *P* = 0.004**, AIC = 159.78 | Intercept | 19.154 | 5.068 | 3.779 | 0.000*** |
|  |  |  | Organic C | 3.794 | 1.878 | 2.021 | 0.049* |
|  |  |  | Edge distance | -0.014 | 0.010 | -1.440 | 0.157ns |
|  | ΔLDMC | RSE = 7.197, df = 41, *R^2^* = 0.161, Adj. *R^2^* = 0.117, *F* = 1.92, *P* = 0.046*, AIC = 182.33 | Intercept | 22.802 | 10.221 | 2.231 | 0.031* |
|  |  |  | Organic C | 6.636 | 2.920 | 2.273 | 0.029* |
|  |  |  | Silt | -0.578 | 0.427 | -1.353 | 0.184ns |
|  |  |  | Edge distance | -0.047 | 0.026 | -1.801 | 0.079ns |
|  | ΔLNC | RSE = 18.113, df = 43, *R^2^* = 0.111, Adj. *R^2^* = 0.090, *F* = 5.345, *P* = 0.030*, AIC = 262.62 | Intercept | 51.123 | 9.662 | 5.291 | 0.000*** |
|  |  |  | Organic C | 11.734 | 5.075 | 2.312 | 0.026* |
|  | ΔLPC | RSE = 17.531, df = 43, *R^2^* = 0.154, Adj. *R^2^* = 0.113, *F* = 2.458, *P* = 0.042*, AIC = 259.69 | Intercept | 55.531 | 9.352 | 5.938 | 0.000*** |
|  |  |  | Organic C | 7.701 | 4.913 | 1.968 | 0.042* |
|  | ΔGs_max_ | RSE = 21.722, df = 43, *R^2^* = 0.165, Adj. *R^2^* = 0.113, *F* = 2.981, *P* = 0.049*, AIC = 278.99 | Intercept | 50.988 | 11.589 | 4.400 | 0.000*** |
|  |  |  | Organic C | 10.511 | 6.088 | 1.987 | 0.049* |
|  | ΔA_max_ | RSE = 20.902, df = 41, *R^2^* = 0.285, Adj. *R^2^* = 0.213, *F* = 3.982, *P* = 0.008**, AIC = 278.29 | Intercept | 70.163 | 29.687 | 2.363 | 0.023* |
|  |  |  | Organic C | 28.246 | 8.481 | 3.331 | 0.002** |
|  |  |  | Silt | -2.444 | 1.240 | -1.972 | 0.056ns |
|  |  |  | Edge distance | -0.135 | 0.076 | -1.778 | 0.083ns |
|  | ΔWUEi | RSE = 32.124, df = 41, *R^2^* = 0.246, Adj. *R^2^* = 0.170, *F* = 3.26, *P* = 0.021*, AIC = 316.96 | Intercept | 100.222 | 45.623 | 2.197 | 0.034* |
|  |  |  | Organic C | 28.333 | 13.033 | 2.174 | 0.036* |
|  |  |  | Silt | -3.292 | 1.905 | -1.728 | 0.092ns |
|  |  |  | Edge distance | -0.321 | 0.117 | -2.744 | 0.009** |
|  | ΔΨ_dawn_ | RSE = 26.781, df = 41, *R^2^* = 0.182, Adj. *R^2^* = 0.115, *F* = 1.515, *P* = 0.042*, AIC = 300.59 | Intercept | -79.937 | 37.779 | -2.116 | 0.041* |
|  |  |  | Organic C | -21.531 | 10.772 | -1.999 | 0.045* |
|  |  |  | Silt | 2.599 | 1.484 | 1.751 | 0.088ns |
|  |  |  | Patch size | 0.385 | 0.276 | 1.397 | 0.170ns |
|  | ΔΨ_noon_ | RSE = 22.741, df = 43, *R^2^* = 0.152, Adj. *R^2^* = 0.112, *F* = 2.973, *P* = 0.042*, AIC = 284.06 | Intercept | -62.840 | 15.999 | -3.928 | 0.000*** |
|  |  |  | Edge distance | 0.175 | 0.073 | 2.413 | 0.020* |
|  | ΔChl | RSE = 21.851, df = 42, *R^2^* = 0.250, Adj. *R^2^* = 0.195, *F* = 4.559, *P* = 0.008*, AIC = 281.43 | Intercept | 14.106 | 25.315 | 0.557 | 0.580ns |
|  |  |  | Organic C | 19.399 | 7.282 | 2.664 | 0.011ns |
|  |  |  | Edge distance | -0.146 | 0.070 | -2.070 | 0.045* |
|  | ΔLL | RSE = 6.886, df = 43, *R^2^* = 0.156, Adj. *R^2^* = 0.113, *F* = 2.557, *P* = 0.047*, AIC = 175.61 | Intercept | 20.949 | 3.674 | 5.701 | 0.000*** |
|  |  |  | Organic C | 3.086 | 1.930 | 1.999 | 0.047* |
|  | ΔLA | RSE = 16.573, df = 43, *R^2^* = 0.100, Adj. *R^2^* = 0.079, *F* = 4.784, *P* = 0.034*, AIC = 254.62 | Intercept | 48.975 | 8.840 | 5.540 | 0.000*** |
|  |  |  | Organic C | 10.156 | 4.644 | 2.187 | 0.034* |
|  | ΔSDWT | RSE = 17.432, df = 44, AIC = 258.21 | Intercept | 68.118 | 2.598 | 26.220 | 0.000*** |
|  |  |  | NA | NA | NA | NA | NA |
|  | ΔHTDBH | RSE = 26.281, df = 43, *R^2^* = 0.063, Adj. *R^2^* = 0.019, *F* = 1.421, *P* = 0.045*, AIC = 297.13 | Intercept | 72.151 | 18.493 | 3.901 | 0.000*** |
|  |  |  | Edge distance | -0.141 | 0.084 | -1.986 | 0.049* |
|  | ΔCDDBH | RSE = 27.812, df = 43, *R^2^* = 0.055, Adj. *R^2^* = 0.033, *F* = 2.492, *P* = 0.122ns, AIC = 301.23 | Intercept | 102.924 | 6.267 | 16.425 | 0.000*** |
|  |  |  | Patch size | -0.283 | 0.179 | -1.579 | 0.122ns |
|  | ΔCCDBH | RSE = 32.512, df = 44, AIC = 314.33 | Intercept | 93.640 | 4.847 | 19.320 | 0.000*** |
|  |  |  | NA | NA | NA | NA | NA |
| HWLD | ΔWSG | RSE = 7.784, df = 41, *R^2^* = 0.186, Adj. *R^2^* = 0.126, *F* = 3.115, *P* = 0.036*, AIC = 188.49 | Intercept | -1.353 | 11.094 | -0.122 | 0.904ns |
|  |  |  | Total P | 152.650 | 96.419 | 1.583 | 0.121ns |
|  |  |  | Clay | 0.700 | 0.350 | 2.004 | 0.042* |
|  |  |  | Silt | 0.557 | 0.354 | 1.576 | 0.123ns |
|  | ΔQWsat | RSE = 6.879, df = 40, *R^2^* = 0.248, Adj. *R^2^* = 0.173, *F* = 3.294, *P* = 0.020*, AIC = 178.27 | Intercept | 7.112 | 9.550 | 0.745 | 0.461ns |
|  |  |  | Clay | 1.118 | 0.754 | 1.482 | 0.146ns |
|  |  |  | Silt | 0.521 | 0.313 | 1.662 | 0.104ns |
|  |  |  | Patch size | 0.137 | 0.071 | 1.996 | 0.041* |
|  |  |  | Edge distance | -0.041 | 0.025 | -1.641 | 0.109ns |
|  | ΔCC | RSE = 6.997, df = 41, *R^2^* = 0.208, Adj. *R^2^* = 0.150, *F* = 3.597, *P* = 0.021*, AIC = 178.93 | Intercept | 5.449 | 9.713 | 0.561 | 0.578ns |
|  |  |  | Clay | 1.875 | 0.704 | 2.663 | 0.011* |
|  |  |  | Silt | 0.541 | 0.317 | 1.705 | 0.096ns |
|  |  |  | Edge distance | -0.044 | 0.025 | -1.737 | 0.099ns |
|  | ΔSLA | RSE = 5.654, df = 41, *R^2^* = 0.273, Adj. *R^2^* = 0.220, *F* = 5.133, *P* = 0.004**, AIC = 159.73 | Intercept | 0.945 | 7.849 | 0.120 | 0.905ns |
|  |  |  | Clay | 1.644 | 0.569 | 2.890 | 0.006** |
|  |  |  | Silt | 0.576 | 0.256 | 2.246 | 0.030* |
|  |  |  | Edge distance | -0.034 | 0.021 | -1.661 | 0.104ns |
|  | ΔRWC | RSE = 5.479, df = 42, *R^2^* = 0.196, Adj. *R^2^* = 0.153, *F* = 2.24, *P* = 0.032*, AIC = 155.98 | Intercept | 4.906 | 7.074 | 0.693 | 0.492ns |
|  |  |  | Silt | 0.458 | 0.247 | 1.955 | 0.048* |
|  |  |  | Patch size | 0.057 | 0.037 | 1.528 | 0.134ns |
|  | ΔLDMC | RSE = 7.323, df = 40, *R^2^* = 0.319, Adj. *R^2^* = 0.251, *F* = 4.687, *P* = 0.003**, AIC = 183.85 | Intercept | 0.265 | 10.161 | 0.026 | 0.979ns |
|  |  |  | Clay | 1.567 | 0.803 | 1.982 | 0.048* |
|  |  |  | Silt | 0.777 | 0.333 | 2.329 | 0.025* |
|  |  |  | Patch size | 0.108 | 0.076 | 1.420 | 0.163ns |
|  |  |  | Edge distance | -0.037 | 0.027 | -1.387 | 0.173ns |
|  | ΔLNC | RSE = 17.491, df = 40, *R^2^* = 0.246, Adj. *R^2^* = 0.171, *F* = 3.267, *P* = 0.021*, AIC = 262.26 | Intercept | 16.834 | 24.283 | 0.693 | 0.492ns |
|  |  |  | Clay | 3.052 | 1.918 | 1.591 | 0.119ns |
|  |  |  | Silt | 1.406 | 0.797 | 1.964 | 0.045* |
|  |  |  | Patch size | 0.297 | 0.181 | 1.638 | 0.109ns |
|  |  |  | Edge distance | -0.097 | 0.064 | -1.513 | 0.138ns |
|  | ΔLPC | RSE = 16.752, df = 40, *R^2^* = 0.310, Adj. *R^2^* = 0.241, *F* = 4.49, *P* = 0.004**, AIC = 258.37 | Intercept | 13.141 | 23.255 | 0.565 | 0.575ns |
|  |  |  | Clay | 3.876 | 1.837 | 2.110 | 0.041* |
|  |  |  | Silt | 1.296 | 0.763 | 1.698 | 0.097ns |
|  |  |  | Patch size | 0.246 | 0.174 | 1.417 | 0.164ns |
|  |  |  | Edge distance | -0.097 | 0.061 | -1.584 | 0.121ns |
|  | ΔGs_max_ | RSE = 17.491, df = 42, *R^2^* = 0.137, Adj. *R^2^* = 0.096, *F* = 3.335, *P* = 0.045*, AIC = 260.44 | Intercept | 9.105 | 23.884 | 0.381 | 0.705ns |
|  |  |  | Clay | 1.775 | 0.756 | 2.348 | 0.023* |
|  |  |  | Silt | 1.381 | 0.790 | 1.747 | 0.088ns |
|  | ΔA_max_ | RSE = 20.412, df = 41, *R^2^* = 0.216, Adj. *R^2^* = 0.159, *F* = 3.767, *P* = 0.018*, AIC = 275.26 | Intercept | 3.262 | 28.335 | 0.115 | 0.909ns |
|  |  |  | Clay | 5.236 | 2.054 | 2.549 | 0.015* |
|  |  |  | Silt | 2.103 | 0.926 | 2.272 | 0.028* |
|  |  |  | Edge distance | -0.135 | 0.074 | -1.815 | 0.077ns |
|  | ΔWUEi | RSE = 27.483, df = 41, *R^2^* = 0.151, Adj. *R^2^* = 0.119, *F* = 2.432, *P* = 0.049*, AIC = 302.03 | Intercept | 30.168 | 38.149 | 0.791 | 0.434ns |
|  |  |  | Clay | 6.039 | 2.765 | 2.184 | 0.035* |
|  |  |  | Silt | 1.959 | 1.246 | 1.572 | 0.124ns |
|  |  |  | Edge distance | -0.203 | 0.100 | -2.035 | 0.048* |
|  | ΔΨ_dawn_ | RSE = 28.271, df = 41, *R^2^* = 0.236, Adj. *R^2^* = 0.180, *F* = 4.215, *P* = 0.011*, AIC = 304.58 | Intercept | -3.333 | 39.248 | -0.085 | 0.933ns |
|  |  |  | Clay | -8.105 | 2.845 | -2.849 | 0.007** |
|  |  |  | Silt | -2.382 | 1.282 | -1.857 | 0.070ns |
|  |  |  | Edge distance | 0.187 | 0.103 | 1.823 | 0.076ns |
|  | ΔΨ_noon_ | RSE = 23.952, df = 41, *R^2^* = 0.285, Adj. *R^2^* = 0.233, *F* = 5.448, *P* = 0.003**, AIC = 289.65 | Intercept | 17.088 | 34.137 | 0.501 | 0.619ns |
|  |  |  | Total P | -517.259 | 296.697 | -1.743 | 0.089ns |
|  |  |  | Clay | -3.198 | 1.075 | -2.974 | 0.005** |
|  |  |  | Silt | -2.118 | 1.088 | -1.947 | 0.058ns |
|  | ΔChl | RSE = 18.682, df = 40, *R^2^* = 0.263, Adj. *R^2^* = 0.189, *F* = 3.564, *P* = 0.014*, AIC = 268.19 | Intercept | 19.605 | 25.935 | 0.756 | 0.454ns |
|  |  |  | Clay | 4.623 | 2.049 | 2.257 | 0.029* |
|  |  |  | Silt | 1.548 | 0.851 | 1.819 | 0.076ns. |
|  |  |  | Patch size | 0.264 | 0.194 | 1.366 | 0.180ns |
|  |  |  | Edge distance | -0.156 | 0.068 | -2.292 | 0.027* |
|  | ΔLL | RSE = 6.667, df = 40, *R^2^* = 0.321, Adj. *R^2^* = 0.253, *F* = 4.729, *P* = 0.003**, AIC = 175.44 | Intercept | -3.836 | 9.255 | -0.414 | 0.681ns |
|  |  |  | Clay | 1.307 | 0.731 | 1.788 | 0.081ns |
|  |  |  | Silt | 0.817 | 0.304 | 2.689 | 0.010* |
|  |  |  | Patch size | 0.119 | 0.069 | 1.720 | 0.093ns |
|  |  |  | Edge distance | -0.037 | 0.024 | -1.525 | 0.135ns |
|  | ΔLA | RSE = 19.312, df = 41, *R^2^* = 0.224, Adj. *R^2^* = 0.167, *F* = 3.94, *P* = 0.015*, AIC = 270.27 | Intercept | 13.820 | 26.900 | 0.514 | 0.610ns |
|  |  |  | Total N | -251.938 | 156.200 | -1.613 | 0.114ns |
|  |  |  | Silt | 2.559 | 0.927 | 2.761 | 0.009** |
|  |  |  | Patch size | 0.443 | 0.148 | 2.999 | 0.005** |
|  | ΔSDWT | RSE = 26.283, df = 41, *R^2^* = 0.258, Adj. *R^2^* = 0.204, *F* = 4.755, *P* = 0.006**, AIC = 261.83 | Intercept | 2.686 | 24.406 | 0.110 | 0.913ns |
|  |  |  | Clay | 5.341 | 1.769 | 3.019 | 0.004** |
|  |  |  | Silt | 1.627 | 0.797 | 2.039 | 0.048* |
|  |  |  | Edge distance | -0.125 | 0.064 | -1.953 | 0.058ns |
|  | ΔHTDBH | RSE = 30.741, df = 42, *R^2^* = 0.144, Adj. *R^2^* = 0.104, *F* = 3.541, *P* = 0.038*, AIC = 311.18 | Intercept | -1.517 | 41.974 | -0.036 | 0.971ns |
|  |  |  | Clay | 3.346 | 1.329 | 2.519 | 0.016* |
|  |  |  | Silt | 2.213 | 1.389 | 1.593 | 0.119ns |
|  | ΔCDDBH | RSE = 29.622, df = 43, *R^2^* = 0.055, Adj. *R^2^* = 0.033, *F* = 2.502, *P* = 0.121ns, AIC = 306.91 | Intercept | 33.455 | 34.700 | 0.964 | 0.340ns |
|  |  |  | Silt | 2.014 | 1.273 | 1.582 | 0.121ns |
|  | ΔCCDBH | RSE = 24.432, df = 44, AIC = 288.61 | Intercept | 77.782 | 3.641 | 21.36 | 0.000*** |
|  |  |  | NA | NA | NA | NA | NA |
| TDF | ΔWSG | RSE = 4.387, df = 43, *R^2^* = 0.162, Adj. *R^2^* = 0.122, *F* = 4.048, *P* = 0.025*, AIC = 135.97 | Intercept | 8.211 | 5.340 | 1.538 | 0.132ns |
|  |  |  | Organic C | 3.694 | 1.605 | 2.302 | 0.026* |
|  | ΔQWsat | RSE = 4.133, df = 41, *R^2^* = 0.193, Adj. *R^2^* = 0.134, *F* = 3.264, *P* = 0.031*, AIC = 131.51 | Intercept | 17.491 | 3.819 | 4.580 | 0.000*** |
|  |  |  | Organic C | 3.068 | 1.424 | 2.155 | 0.037* |
|  |  |  | Clay | 1.113 | 0.435 | 2.556 | 0.014* |
|  |  |  | Edge distance | -0.035 | 0.015 | -2.319 | 0.026* |
|  | ΔCC | RSE = 3.958, df = 41, *R^2^* = 0.189, Adj. *R^2^* = 0.130, *F* = 3.185, *P* = 0.034*, AIC = 127.63 | Intercept | 18.605 | 3.657 | 5.087 | 0.000*** |
|  |  |  | Organic C | 2.415 | 1.364 | 1.771 | 0.084ns |
|  |  |  | Clay | 1.219 | 0.417 | 2.923 | 0.006** |
|  |  |  | Edge distance | -0.036 | 0.014 | -2.484 | 0.017* |
|  | ΔSLA | RSE = 3.458, df = 41, *R^2^* = 0.213, Adj. *R^2^* = 0.155, *F* = 3.695, *P* = 0.019*, AIC = 115.46 | Intercept | 10.518 | 4.800 | 2.191 | 0.034* |
|  |  |  | Clay | 0.904 | 0.348 | 2.598 | 0.013* |
|  |  |  | Silt | 0.348 | 0.157 | 2.218 | 0.032* |
|  |  |  | Edge distance | -0.026 | 0.013 | -2.032 | 0.048* |
|  | ΔRWC | RSE = 3.245, df = 42, *R^2^* = 0.245, Adj. *R^2^* = 0.209, *F* = 6.801, *P* = 0.003**, AIC = 108.85 | Intercept | 13.691 | 4.498 | 3.044 | 0.004** |
|  |  |  | Silt | 0.305 | 0.147 | 2.075 | 0.044* |
|  |  |  | Edge distance | -0.011 | 0.005 | -2.241 | 0.030* |
|  | ΔLDMC | RSE = 4.491, df = 41, *R^2^* = 0.209, Adj. *R^2^* = 0.152, *F* = 3.619, *P* = 0.021*, AIC = 139.00 | Intercept | 13.067 | 6.234 | 2.096 | 0.042* |
|  |  |  | Clay | 1.127 | 0.452 | 2.495 | 0.017* |
|  |  |  | Silt | 0.451 | 0.204 | 2.212 | 0.033* |
|  |  |  | Edge distance | -0.029 | 0.016 | -1.746 | 0.088ns |
|  | ΔLNC | RSE = 10.612, df = 42, *R^2^* = 0.176, Adj. *R^2^* = 0.115, *F* = 2.913, *P* = 0.046*, AIC = 216.28 | Intercept | 12.770 | 17.474 | 0.731 | 0.469ns |
|  |  |  | Organic C | 6.064 | 4.259 | 1.424 | 0.162ns |
|  |  |  | Silt | 0.934 | 0.541 | 1.986 | 0.049* |
|  | ΔLPC | RSE = 10.992, df = 41, *R^2^* = 0.163, Adj. *R^2^* = 0.112, *F* = 2.658, *P* = 0.041*, AIC = 219.52 | Intercept | 45.481 | 10.153 | 4.480 | 0.000*** |
|  |  |  | Organic C | 6.452 | 3.786 | 1.704 | 0.096ns |
|  |  |  | Clay | 3.080 | 1.158 | 2.660 | 0.011* |
|  |  |  | Edge distance | -0.084 | 0.040 | -2.115 | 0.041* |
|  | ΔGs_max_ | RSE = 12.173, df = 42, *R^2^* = 0.185, Adj. *R^2^* = 0.122, *F* = 2.135, *P* = 0.041*, AIC = 228.73 | Intercept | 31.934 | 15.155 | 2.107 | 0.041* |
|  |  |  | Organic C | 8.522 | 4.462 | 1.990 | 0.048* |
|  |  |  | Edge distance | -0.062 | 0.035 | -1.779 | 0.083ns |
|  | ΔA_max_ | RSE = 13.321, df = 41, *R^2^* = 0.231, Adj. *R^2^* = 0.175, *F* = 4.101, *P* = 0.012*, AIC = 236.84 | Intercept | 44.039 | 12.307 | 3.578 | 0.000*** |
|  |  |  | Organic C | 12.476 | 4.589 | 2.719 | 0.009** |
|  |  |  | Clay | 3.278 | 1.404 | 2.336 | 0.024* |
|  |  |  | Edge distance | -0.104 | 0.048 | -2.153 | 0.037* |
|  | ΔWUEi | RSE = 19.232, df = 39, *R^2^* = 0.313, Adj. *R^2^* = 0.225, *F* = 3.553, *P* = 0.009**, AIC = 271.65 | Intercept | -25.816 | 76.382 | -0.338 | 0.737ns |
|  |  |  | Organic C | 14.220 | 6.696 | 2.124 | 0.040* |
|  |  |  | Clay | 4.437 | 2.062 | 2.151 | 0.037* |
|  |  |  | Bulk density | 75.239 | 53.879 | 1.396 | 0.170ns |
|  |  |  | Edge distance | -0.201 | 0.070 | -2.887 | 0.006** |
|  |  |  | Mortality index | -1.142 | 0.700 | -1.633 | 0.111ns |
|  | ΔΨ_dawn_ | RSE = 16.271, df = 40, *R^2^* = 0.224, Adj. *R^2^* = 0.147, *F* = 2.89, *P* = 0.034*, AIC = 255.74 | Intercept | -54.603 | 15.642 | -3.491 | 0.001** |
|  |  |  | Organic C | -12.427 | 5.611 | -2.215 | 0.032* |
|  |  |  | Clay | -3.969 | 1.738 | -2.284 | 0.028* |
|  |  |  | Edge distance | 0.087 | 0.059 | 1.470 | 0.149ns |
|  |  |  | Mortality index | 0.829 | 0.592 | 1.400 | 0.169ns |
|  | ΔΨ_noon_ | RSE = 13.342, df = 41, *R^2^* = 0.272, Adj. *R^2^* = 0.219, *F* = 5.113, *P* = 0.004**, AIC = 237.01 | Intercept | -54.531 | 12.330 | -4.422 | 0.000*** |
|  |  |  | Organic C | -8.499 | 4.598 | -1.849 | 0.072ns |
|  |  |  | Clay | -5.368 | 1.406 | -3.817 | 0.000*** |
|  |  |  | Edge distance | 0.144 | 0.048 | 2.970 | 0.004** |
|  | ΔChl | RSE = 10.301, df = 40, *R^2^* = 0.394, Adj. *R^2^* = 0.333, *F* = 6.488, *P* = 0.000***, AIC = 214.56 | Intercept | 49.902 | 9.899 | 5.041 | 0.000*** |
|  |  |  | Organic C | 11.575 | 3.551 | 3.260 | 0.002** |
|  |  |  | Clay | 4.115 | 1.100 | 3.742 | 0.000*** |
|  |  |  | Edge distance | -0.140 | 0.037 | -3.745 | 0.000*** |
|  |  |  | Mortality index | -0.569 | 0.375 | -1.519 | 0.136ns |
|  | ΔLL | RSE = 4.511, df = 41, *R^2^* = 0.165, Adj. *R^2^* = 0.114, *F* = 2.7, *P* = 0.048*, AIC = 139.19 | Intercept | 10.124 | 6.247 | 1.621 | 0.113ns |
|  |  |  | Clay | 0.938 | 0.453 | 2.071 | 0.045* |
|  |  |  | Silt | 0.416 | 0.204 | 2.039 | 0.048* |
|  |  |  | Edge distance | -0.024 | 0.016 | -1.491 | 0.144ns |
|  | ΔLA | RSE = 10.512, df = 42, *R^2^* = 0.248, Adj. *R^2^* = 0.193, *F* = 4.517, *P* = 0.008**, AIC = 215.54 | Intercept | 4.894 | 16.544 | 0.296 | 0.769ns |
|  |  |  | Silt | 1.469 | 0.489 | 3.007 | 0.004** |
|  |  |  | Edge distance | -0.060 | 0.030 | -2.014 | 0.051ns |
|  | ΔSDWT | RSE = 10.821, df = 41, *R^2^* = 0.181, Adj. *R^2^* = 0.121, *F* = 3.026, *P* = 0.040*, AIC = 217.94 | Intercept | 32.056 | 14.987 | 2.139 | 0.038* |
|  |  |  | Clay | 2.678 | 1.086 | 2.466 | 0.018* |
|  |  |  | Silt | 0.909 | 0.490 | 1.856 | 0.071ns |
|  |  |  | Edge distance | -0.081 | 0.039 | -2.051 | 0.047* |
|  | ΔHTDBH | RSE = 19.982, df = 43, *R^2^* = 0.062, Adj. *R^2^* = 0.040, *F* = 2.823, *P* = 0.100ns, AIC = 271.49 | Intercept | 40.657 | 23.411 | 1.737 | 0.089ns |
|  |  |  | Silt | 1.443 | 0.859 | 1.680 | 0.100ns |
|  | ΔCDDBH | RSE = 18.622, df = 43, *R^2^* = 0.086, Adj. *R^2^* = 0.065, *F* = 4.042, *P* = 0.051ns, AIC = 265.14 | Intercept | 41.885 | 21.816 | 1.920 | 0.062ns |
|  |  |  | Silt | 1.609 | 0.800 | 2.011 | 0.051ns |
|  | ΔCCDBH | RSE = 16.961, df = 43, *R^2^* = 0.076, Adj. *R^2^* = 0.054, *F* = 3.53, *P* = 0.067ns, AIC = 256.73 | Intercept | 63.107 | 9.049 | 6.974 | 0.000*** |
|  |  |  | Organic C | 8.931 | 4.754 | 1.879 | 0.067ns |

Table S7. Summary of multiple regression model coefficients for predicting community weighted means of plasticity in functional traits of tree species categorized into three functional types (viz., LWHD, low wood density and high deciduous; HWMD, high wood density and medium deciduous; high wood density and low deciduous) along the edge distance gradient across the 45 forest fragments. WSG, wood specific gravity (g cm^-3^); QWsat, stem water storage capacity (%); CC, canopy cover intensity (%); SLA, specific leaf area (cm^2^ g^-1^); RWC, relative water content (%); LDMC, leaf dry matter content (%); LNC, leaf nitrogen content (% dry weight); LPC, leaf phosphorus content (% dry weight); Gs_max_, maximum saturated stomatal conductance (mol m^-2^ s^-1^); A_max_, maximum saturated photosynthesis (µmol m^-2^ s^-1^); WUEi, intrinsic water use efficiency (µmol mol^-1^); Ψ_dawn_, leaf water potential at dawn (MPa); Ψ_noon_, leaf water potential at noon (MPa); Chl, chlorophyll content (mg g^-1^ fresh weight); LL, leaf life-span (days); LA, leaf area (cm^2^); SDWT, seed mass (g); HTDBH, ratio of total height and diameter at breast height; CDDBH, ratio of crown depth and diameter at breast height; CCDBH, ratio of crown cover and diameter at breast height. The “Δ” sign represents plasticity. nsP > 0.05, *P < 0.05, **P < 0.01, ***P < 0.001.

| **Functional Trait** | **ANOVA/Correlation** | **Factor** | **Estimate** | **Std. Error** | ***t*-value** | ***P*-value** |
| --- | --- | --- | --- | --- | --- | --- |
| ΔWSG | RSE = 0.789; R^2^ = 0.035; Adj.R^2^ = 0.026; F_5,567_ = 4.105; *P* = 0.001**  ***Correlation with edge distance:***  LWHD (R = 0.12; *P* = 0.049*)  HWMD (R = -0.068; *P* = 0.28ns)  HWLD (R = 0.023; *P* = 0.72ns) | (Intercept) | 2.7676 | 0.1413 | 19.583 | 0.000*** |
|  |  | Edge distance | 0.0002 | 0.0005 | 0.374 | 0.709ns |
|  |  | LWHD | -0.4866 | 0.3070 | -1.585 | 0.114ns |
|  |  | HWMD | 0.3024 | 0.2026 | 1.492 | 0.136ns |
|  |  | Edge distance × LWHD | 0.0006 | 0.0011 | 0.525 | 0.600ns |
|  |  | Edge distance × HWMD | -0.0008 | 0.0007 | -1.029 | 0.304ns |
| ΔQWsat | RSE = 0.758; R^2^ = 0.088; Adj.R^2^ = 0.080; F_5,567_ = 10.94; *P* = 0.000***  ***Correlation with edge distance:***  LWHD (R = 0.039; *P* = 0.73ns)  HWMD (R = - 0.17; *P* = 0.009**)  HWLD (R = - 0.042; *P* = 0.51ns) | (Intercept) | 2.9423 | 0.1358 | 21.674 | 0.000*** |
|  |  | Edge distance | -0.0003 | 0.0005 | -0.677 | 0.499ns |
|  |  | LWHD | -0.6865 | 0.2949 | -2.328 | 0.020* |
|  |  | HWMD | 0.3824 | 0.1947 | 1.965 | 0.049* |
|  |  | Edge distance × LWHD | 0.0006 | 0.0010 | 0.566 | 0.572ns |
|  |  | Edge distance × HWMD | -0.0010 | 0.0007 | -1.410 | 0.159ns |
| ΔCC | RSE = 0.741; R^2^ = 0.051; Adj.R^2^ = 0.043; F_5,567_ = 6.092; *P* = 0.000***  ***Correlation with edge distance:***  LWHD (R = 0.064; *P* = 0.56ns)  HWMD (R = 0.0002; *P* = 1.00ns)  HWLD (R = 0.14; *P* = 0.034*) | (Intercept) | 2.8739 | 0.1327 | 21.653 | 0.000*** |
|  |  | Edge distance | 0.0000 | 0.0005 | 0.002 | 0.998ns |
|  |  | LWHD | -0.4473 | 0.2883 | -1.551 | 0.121ns |
|  |  | HWMD | 0.4074 | 0.1903 | 2.141 | 0.033* |
|  |  | Edge distance × LWHD | 3.64e-04 | 9.85e-04 | 0.370 | 0.712ns |
|  |  | Edge distance × HWMD | -1.05e-03 | 6.87e-04 | -1.522 | 0.129ns |
| ΔSLA | RSE = 0.741; R^2^ = 0.052; Adj.R^2^ = 0.043; F_5,567_ = 6.158; *P* = 0.000***  ***Correlation with edge distance:***  LWHD (R = -0.11; *P* = 0.33ns)  HWMD (R = -0.13; *P* = 0.041*)  HWLD (R = 0.01; *P* = 0.88ns) | (Intercept) | 2.7788 | 0.1328 | 20.931 | 0.000*** |
|  |  | Edge distance | -0.0001 | 0.0005 | -0.154 | 0.878ns |
|  |  | LWHD | -0.5769 | 0.2884 | -2.000 | 0.045* |
|  |  | HWMD | 0.3438 | 0.1904 | 1.806 | 0.072ns |
|  |  | Edge distance × LWHD | 6.94e-04 | 9.85e-04 | 0.704 | 0.482ns |
|  |  | Edge distance × HWMD | -9.50e-04 | 6.87e-04 | -1.383 | 0.167ns |
| ΔRWC | RSE = 0.767; R^2^ = 0.076; Adj.R^2^ = 0.068; F_5,567_ = 9.324; *P* = 0.000***  ***Correlation with edge distance:***  LWHD (R = 0.008; *P* = 0.94ns)  HWMD (R = -0.24; *P* = 0.000***)  HWLD (R = -0.077; *P* = 0.23ns) | (Intercept) | 2.7806 | 0.1375 | 20.221 | 0.000*** |
|  |  | Edge distance | -0.0006 | 0.0005 | -1.220 | 0.223ns |
|  |  | LWHD | -0.5811 | 0.2987 | -1.945 | 0.052ns |
|  |  | HWMD | 0.4837 | 0.1972 | 2.453 | 0.015* |
|  |  | Edge distance × LWHD | 0.0007 | 0.0010 | 0.663 | 0.508ns |
|  |  | Edge distance × HWMD | -0.0013 | 0.0007 | -1.897 | 0.058ns |
| ΔLDMC | RSE = 0.742; R^2^ = 0.060; Adj.R^2^ = 0.052; F_5,567_ = 7.229; *P* = 0.000***  ***Correlation with edge distance:***  LWHD (R = -0.12; *P* = 0.045*)  HWMD (R = 0.019; *P* = 0.77ns)  HWLD (R = 0.11; *P* = 0.30ns) | (Intercept) | 2.9865 | 0.1329 | 22.471 | 0.000*** |
|  |  | Edge distance | 0.0002 | 0.0005 | 0.298 | 0.766ns |
|  |  | LWHD | -0.5689 | 0.2887 | -1.971 | 0.049* |
|  |  | HWMD | 0.4015 | 0.1906 | 2.107 | 0.035* |
|  |  | Edge distance × LWHD | 0.0005 | 0.0010 | 0.520 | 0.603ns |
|  |  | Edge distance × HWMD | -0.0011 | 0.0006 | -1.638 | 0.102ns |
| ΔLNC | RSE = 0.759; R^2^ = 0.057; Adj.R^2^ = 0.049; F_5,567_ = 6.863; *P* = 0.000***  ***Correlation with edge distance:***  LWHD (R = -0.14; *P* = 0.024*)  HWMD (R = 0.072; *P* = 0.51ns)  HWLD (R = -0.008; *P* = 0.91ns) | (Intercept) | 3.8623 | 0.1360 | 28.404 | 0.000*** |
|  |  | Edge distance | -0.0001 | 0.0005 | -0.120 | 0.904ns |
|  |  | LWHD | -0.5573 | 0.2954 | -1.887 | 0.059ns |
|  |  | HWMD | 0.3859 | 0.1950 | 1.979 | 0.048* |
|  |  | Edge distance × LWHD | 5.01e-04 | 1.01e-03 | 0.497 | 0.619ns |
|  |  | Edge distance × HWMD | -1.11e-03 | 7.03e-04 | -1.572 | 0.116ns |
| ΔLPC | RSE = 0.765; R^2^ = 0.049; Adj.R^2^ = 0.040; F_5,567_ = 5.785; *P* = 0.000***  ***Correlation with edge distance:***  LWHD (R = 0.016; *P* = 0.89ns)  HWMD (R = -0.14; *P* = 0.029*)  HWLD (R = 0.012; *P* = 0.85ns) | (Intercept) | 3.7813 | 0.1370 | 27.600 | 0.000*** |
|  |  | Edge distance | 0.0001 | 0.0005 | 0.200 | 0.842ns |
|  |  | LWHD | -0.3655 | 0.2976 | -1.228 | 0.219ns |
|  |  | HWMD | 0.4298 | 0.1965 | 2.188 | 0.029* |
|  |  | Edge distance × LWHD | -4.84e-06 | 1.02e-03 | -0.005 | 0.996ns |
|  |  | Edge distance × HWMD | -1.22e-03 | 7.09e-04 | -1.719 | 0.086ns |
| ΔGs_max_ | RSE = 0.751; R^2^ = 0.059; Adj.R^2^ = 0.051; F_5,567_ = 7.110; *P* = 0.000***  ***Correlation with edge distance:***  LWHD (R = 0.078; *P* = 0.48ns)  HWMD (R = -0.02; *P* = 0.76ns)  HWLD (R = 0.17; *P* = 0.006**) | (Intercept) | 3.8312 | 0.1345 | 28.477 | 0.000*** |
|  |  | Edge distance | -0.0002 | 0.0005 | -0.319 | 0.749ns |
|  |  | LWHD | -0.5592 | 0.2923 | -1.913 | 0.056ns |
|  |  | HWMD | 0.4423 | 0.1929 | 2.293 | 0.022* |
|  |  | Edge distance × LWHD | 0.0006 | 0.0010 | 0.650 | 0.515ns |
|  |  | Edge distance × HWMD | -0.0012 | 0.0006 | -1.767 | 0.077ns |
| ΔA_max_ | RSE = 0.745; R^2^ = 0.062; Adj.R^2^ = 0.053; F_5,567_ = 7.429; *P* = 0.000***  ***Correlation with edge distance:***  LWHD (R = -0.16; *P* = 0.041*)  HWMD (R = 0.053; *P* = 0.41ns)  HWLD (R = -0.18; *P* = 0.004**) | (Intercept) | 4.0294 | 0.1336 | 30.167 | 0.000*** |
|  |  | Edge distance | -0.0004 | 0.0005 | -0.845 | 0.398ns |
|  |  | LWHD | -0.5653 | 0.2902 | -1.948 | 0.052ns |
|  |  | HWMD | 0.3493 | 0.1915 | 1.824 | 0.068ns |
|  |  | Edge distance × LWHD | 0.0006 | 0.0010 | 0.578 | 0.563ns |
|  |  | Edge distance × HWMD | -0.0010 | 0.0007 | -1.489 | 0.137ns |
| ΔWUEi | RSE = 0.874; R^2^ = 0.047; Adj.R^2^ = 0.039; F_5,567_ = 5.646; *P* = 0.000***  ***Correlation with edge distance:***  LWHD (R = 0.04; *P* = 0.72ns)  HWMD (R = -0.19; *P* = 0.003**)  HWLD (R = -0.13; *P* = 0.039*) | (Intercept) | 4.3237 | 0.1566 | 27.616 | 0.000*** |
|  |  | Edge distance | -0.0013 | 0.0006 | -2.167 | 0.031* |
|  |  | LWHD | -0.6499 | 0.3401 | -1.911 | 0.056ns |
|  |  | HWMD | 0.2987 | 0.2245 | 1.330 | 0.183ns |
|  |  | Edge distance × LWHD | 0.0016 | 0.0012 | 1.337 | 0.182ns |
|  |  | Edge distance × HWMD | -0.0004 | 0.0008 | -0.551 | 0.582ns |
| ΔΨ_dawn_ | RSE = 0.755; R^2^ = 0.051; Adj.R^2^ = 0.043; F_5,567_ = 6.071; *P* = 0.000***  ***Correlation with edge distance:***  LWHD (R = -0.087; *P* = 0.17ns)  HWMD (R = -0.231; *P* = 0.046*)  HWLD (R = -0.175; *P* = 0.049*) | (Intercept) | 4.13e+00 | 1.35e-01 | 30.504 | 0.000*** |
|  |  | Edge distance | 5.99e-05 | 5.03e-04 | 0.119 | 0.905ns |
|  |  | LWHD | -7.32e-01 | 2.94e-01 | -2.491 | 0.013* |
|  |  | HWMD | 2.92e-01 | 1.94e-01 | 1.506 | 0.133ns |
|  |  | Edge distance × LWHD | 1.18e-03 | 1.00e-03 | 1.178 | 0.239ns |
|  |  | Edge distance × HWMD | -7.51e-04 | 7.00e-04 | -1.073 | 0.284ns |
| ΔΨ_noon_ | RSE = 0.784; R^2^ = 0.055; Adj.R^2^ = 0.047; F_5,567_ = 6.603; *P* = 0.000***  ***Correlation with edge distance:***  LWHD (R = -0.068; *P* = 0.54ns)  HWMD (R = -0.14; *P* = 0.034*)  HWLD (R = -0.12; *P* = 0.047*) | (Intercept) | 3.9985 | 0.1405 | 28.465 | 0.000*** |
|  |  | Edge distance | 0.0002 | 0.0005 | 0.335 | 0.737ns |
|  |  | LWHD | -0.5103 | 0.3052 | -1.672 | 0.095ns |
|  |  | HWMD | 0.4259 | 0.2014 | 2.114 | 0.034* |
|  |  | Edge distance × LWHD | 0.0003 | 0.0010 | 0.247 | 0.805ns |
|  |  | Edge distance × HWMD | -0.0013 | 0.0007 | -1.765 | 0.078ns |
| ΔChl | RSE = 0.756; R^2^ = 0.058; Adj.R^2^ = 0.049; F_5,567_ = 6.972; *P* = 0.000***  ***Correlation with edge distance:***  LWHD (R = -0.0028; *P* = 0.98ns)  HWMD (R = -0.18; *P* = 0.006**)  HWLD (R = 0.028; *P* = 0.66ns) | (Intercept) | 3.9646 | 0.1354 | 29.283 | 0.000*** |
|  |  | Edge distance | -0.0002 | 0.0005 | -0.449 | 0.654ns |
|  |  | LWHD | -0.4502 | 0.2941 | -1.531 | 0.126ns |
|  |  | HWMD | 0.4055 | 0.1941 | 2.089 | 0.037* |
|  |  | Edge distance × LWHD | 0.0002 | 0.0010 | 0.208 | 0.835ns |
|  |  | Edge distance × HWMD | -0.0012 | 0.0007 | -1.677 | 0.094ns |
| ΔLL | RSE = 0.756; R^2^ = 0.057; Adj.R^2^ = 0.049; F_5,567_ = 6.867; *P* = 0.000***  ***Correlation with edge distance:***  LWHD (R = -0.14; *P* = 0.047*)  HWMD (R = -0.12; *P* = 0.045*)  HWLD (R = -0.014; *P* = 0.83ns) | (Intercept) | 2.7999 | 0.1355 | 20.668 | 0.000*** |
|  |  | Edge distance | 0.0001 | 0.0005 | 0.223 | 0.824ns |
|  |  | LWHD | -0.6164 | 0.2943 | -2.094 | 0.037* |
|  |  | HWMD | 0.4139 | 0.1943 | 2.131 | 0.034* |
|  |  | Edge distance × LWHD | 0.0008 | 0.0010 | 0.772 | 0.441ns |
|  |  | Edge distance × HWMD | -0.0011 | 0.0007 | -1.561 | 0.119ns |
| ΔLA | RSE = 0.766; R^2^ = 0.065; Adj.R^2^ = 0.057; F_5,567_ = 7.853; *P* = 0.000***  ***Correlation with edge distance:***  LWHD (R = -0.086; *P* = 0.44ns)  HWMD (R = -0.13; *P* = 0.037*)  HWLD (R = -0.18; *P* = 0.048*) | (Intercept) | 3.81e+00 | 1.37e-01 | 27.792 | 0.000*** |
|  |  | Edge distance | -7.30e-05 | 5.11e-04 | -0.143 | 0.886ns |
|  |  | LWHD | -6.42e-01 | 2.98e-01 | -2.152 | 0.032* |
|  |  | HWMD | 3.49e-01 | 1.97e-01 | 1.773 | 0.077ns |
|  |  | Edge distance × LWHD | 6.03e-04 | 1.02e-03 | 0.592 | 0.554ns |
|  |  | Edge distance × HWMD | -9.73e-04 | 7.10e-04 | -1.371 | 0.171ns |
| ΔSDWT | RSE = 0.740; R^2^ = 0.072; Adj.R^2^ = 0.064; F_5,567_ = 8.778; *P* = 0.000***  ***Correlation with edge distance:***  LWHD (R = -0.13; *P* = 0.049*)  HWMD (R = -0.13; *P* = 0.041*)  HWLD (R = -0.011; *P* = 0.87ns) | (Intercept) | 3.76e+00 | 1.33e-01 | 28.371 | 0.000*** |
|  |  | Edge distance | 8.44e-05 | 4.94e-04 | 0.171 | 0.864ns |
|  |  | LWHD | -6.51e-01 | 2.88e-01 | -2.257 | 0.024* |
|  |  | HWMD | 4.10e-01 | 1.90e-01 | 2.157 | 0.031* |
|  |  | Edge distance × LWHD | 6.45e-04 | 9.84e-04 | 0.655 | 0.513ns |
|  |  | Edge distance × HWMD | -1.12e-03 | 6.86e-04 | -1.634 | 0.103ns |
| ΔHTDBH | RSE = 0.812; R^2^ = 0.043; Adj.R^2^ = 0.035; F_5,567_ = 5.113; *P* = 0.000***  ***Correlation with edge distance:***  LWHD (R = 10.16; *P* = 0.049*)  HWMD (R = -0.093; *P* = 0.14ns)  HWLD (R = 0.0042; *P* = 0.95ns) | (Intercept) | 4.03e+00 | 1.46e-01 | 27.716 | 0.000*** |
|  |  | Edge distance | 3.76e-05 | 5.41e-04 | 0.069 | 0.945ns |
|  |  | LWHD | -4.97e-01 | 3.16e-01 | -1.571 | 0.117ns |
|  |  | HWMD | 3.07e-01 | 2.09e-01 | 1.471 | 0.142ns |
|  |  | Edge distance × LWHD | 3.41e-04 | 1.08e-03 | 0.315 | 0.753ns |
|  |  | Edge distance × HWMD | -8.05e-04 | 7.53e-04 | -1.069 | 0.286ns |
| ΔCDDBH | RSE = 0.775; R^2^ = 0.059; Adj.R^2^ = 0.051; F_5,567_ = 7.194; *P* = 0.000***  ***Correlation with edge distance:***  LWHD (R = -0.18; *P* = 0.004**)  HWMD (R = 0.038; *P* = 0.73ns)  HWLD (R = -0.084; *P* = 0.19ns) | (Intercept) | 4.3140 | 0.1389 | 31.058 | 0.000*** |
|  |  | Edge distance | -0.0007 | 0.0005 | -1.360 | 0.174ns |
|  |  | LWHD | -0.6630 | 0.3018 | -2.197 | 0.028* |
|  |  | HWMD | 0.2833 | 0.1992 | 1.422 | 0.156ns |
|  |  | Edge distance × LWHD | 0.0009 | 0.0010 | 0.899 | 0.369ns |
|  |  | Edge distance × HWMD | -0.0008 | 0.0007 | -1.144 | 0.253ns |
| ΔCCDBH | RSE = 0.829; R^2^ = 0.060; Adj.R^2^ = 0.052; F_5,567_ = 7.291; *P* = 0.000***  ***Correlation with edge distance:***  LWHD (R = -0.14; *P* = 0.027*)  HWMD (R = -0.007; *P* = 0.95ns)  HWLD (R = -0.055; *P* = 0.39ns) | (Intercept) | 4.1460 | 0.1486 | 27.898 | 0.000*** |
|  |  | Edge distance | -0.0005 | 0.0006 | -0.840 | 0.401ns |
|  |  | LWHD | -0.5963 | 0.3228 | -1.847 | 0.065ns |
|  |  | HWMD | 0.2962 | 0.2131 | 1.390 | 0.165ns |
|  |  | Edge distance × LWHD | 0.0004 | 0.0011 | 0.380 | 0.704ns |
|  |  | Edge distance × HWMD | -0.0008 | 0.0008 | -1.076 | 0.283ns |
